# Iron-dependent epigenetic modulation promotes pathogenic T cell differentiation in lupus

**DOI:** 10.1172/JCI152345

**Published:** 2022-05-02

**Authors:** Xiaofei Gao, Yang Song, Jiali Wu, Shuang Lu, Xiaoli Min, Limin Liu, Longyuan Hu, Meiling Zheng, Pei Du, Yaqin Yu, Hai Long, Haijing Wu, Sujie Jia, Di Yu, Qianjin Lu, Ming Zhao

**Affiliations:** 1 Department of Dermatology, Second Xiangya Hospital, Central South University, Hunan Key Laboratory of Medical Epigenomics, Changsha, China.; 2 Research Unit of Key Technologies of Diagnosis and Treatment for Immune-related Skin Diseases, Chinese Academy of Medical Sciences, Changsha, China.; 3 Clinical Medical Research Center of Major Skin Diseases and Skin Health of Hunan Province, Changsha, China.; 4 Clinical Immunology Research Center, Central South University, Changsha, China.; 5 Department of Pharmacy, Third Xiangya Hospital, Central South University, Changsha, China.; 6 Diamantina Institute, The University of Queensland, Faculty of Medicine, The University of Queensland, Brisbane, Queensland, Australia.; 7 Institute of Dermatology, Chinese Academy of Medical Sciences and Peking Union Medical College, Nanjing, China.

**Keywords:** Autoimmunity, Epigenetics, Lupus, T cells

## Abstract

The trace element iron affects immune responses and vaccination, but knowledge of its role in autoimmune diseases is limited. Expansion of pathogenic T cells, especially T follicular helper (Tfh) cells, has great significance to systemic lupus erythematosus (SLE) pathogenesis. Here, we show an important role of iron in regulation of pathogenic T cell differentiation in SLE. We found that iron overload promoted Tfh cell expansion, proinflammatory cytokine secretion, and autoantibody production in lupus-prone mice. Mice treated with a high-iron diet exhibited an increased proportion of Tfh cell and antigen-specific GC response. Iron supplementation contributed to Tfh cell differentiation. In contrast, iron chelation inhibited Tfh cell differentiation. We demonstrated that the miR-21/BDH2 axis drove iron accumulation during Tfh cell differentiation and further promoted Fe^2+^-dependent TET enzyme activity and *BCL6* gene demethylation. Thus, maintaining iron homeostasis might be critical for eliminating pathogenic Th cells and might help improve the management of patients with SLE.

## Introduction

Systemic lupus erythematosus (SLE) is a heterogeneous autoimmune disease characterized by aberrant differentiation of pathogenic T cells and overproduction of autoantibodies (1). T follicular helper (Tfh) cells are a specific subset of CD4^+^ T helper cells that are mainly localized in the germinal centers (GCs) to help B cell maturation and antibody production (2). The aberrant expansion of Tfh cells is closely related to the progression of autoimmune diseases (3). Indeed, circulating Tfh cells are increased in peripheral blood of patients with SLE and correlate closely to the disease activity (4). Aberrant Tfh cell differentiation triggers lupus-like autoimmunity in mice (5, 6), while inhibiting Tfh cell accumulation reduces autoimmune responses and relieves the disease progression of murine lupus (7). These observations indicate a close link between Tfh cells and SLE pathogenesis, yet the precise mechanism causing Tfh cell expansion in SLE remains unclear.

Iron is an essential trace element that is widely involved in biological processes (8). Iron homeostasis is firmly regulated, and both iron deficiency and iron overload can cause various pathological conditions (9, 10). Recently, substantial advances have been achieved in understanding the role of iron in modulating immune cell functions and related human diseases (11). Intracellular iron drives the pathogenic Th cell differentiation by promoting the production of proinflammatory cytokine GM-CSF in neuroinflammatory diseases (12). Iron deficiency impairs B cell proliferation and antibody responses, suggesting the role of iron in regulating humoral immunity and vaccination (13). Knowledge about the role of iron in SLE development remains limited, and studies have reported variable findings. Some studies reported that sufficient iron status plays a protective role against SLE (14, 15). On the contrary, a case report showed that iron dextran supplementation induced SLE-like symptoms in a childbearing-age woman with iron deficiency anemia (16). In lupus mice, renal iron accumulation occurs in lupus nephritis, and iron chelation treatment delays the onset of albuminuria (17, 18). Furthermore, a recent study reported that hepcidin therapy reduces iron accumulation in the kidney and alleviates the disease progression of lupus nephritis in MRL/*lpr* lupus-prone mice (19), suggesting that modulating iron homeostasis may be a promising therapeutic strategy for lupus nephritis.

TET enzymes oxidize 5-methylcytosine to 5-hydroxymethylcytosine in nucleic acids under the presence of Fe^2+^ and 2-oxoglutarate, driving DNA demethylation/hydroxymethylation and gene transcription (20). Modulating intracellular ferrous iron enhances the activity of TET enzymes and alters DNA methylation to regulate gene expression (21, 22). DNA demethylation/hydroxymethylation plays an important role in modulating CD4^+^ T cell differentiation (23, 24). We have reported that intracellular iron accumulation drives DNA hydroxymethylation and demethylation, promoting gene transcription and CD4^+^ T cell overactivation in lupus (25). So far, the role of TET proteins in Tfh cell differentiation and function remains unclear.

Here, we show that a high-iron diet (HID) favored pathogenic T cell differentiation and autoantibody production, accelerating disease progression in MRL/*lpr* lupus-prone mice. We identified iron accumulation in lupus CD4^+^ T cells, which was related to the increase in Tfh cells in SLE. Mechanistically, miR-21 overexpression repressed 3-hydroxybutyrate dehydrogenase-2 (BDH2) to promote iron accumulation and enhanced the activity of Fe^2+^-dependent TET enzymes in Tfh cells, leading to *BCL6* gene hydroxymethylation and Tfh cell differentiation. Overall, our data show that iron overload favors pathogenic T cell differentiation and autoantibody production, providing strong evidence for the important role of iron homeostasis in lupus pathogenesis.

## Results

### Increased intracellular iron in lupus CD4^+^ T cells.

**Figure 1:**
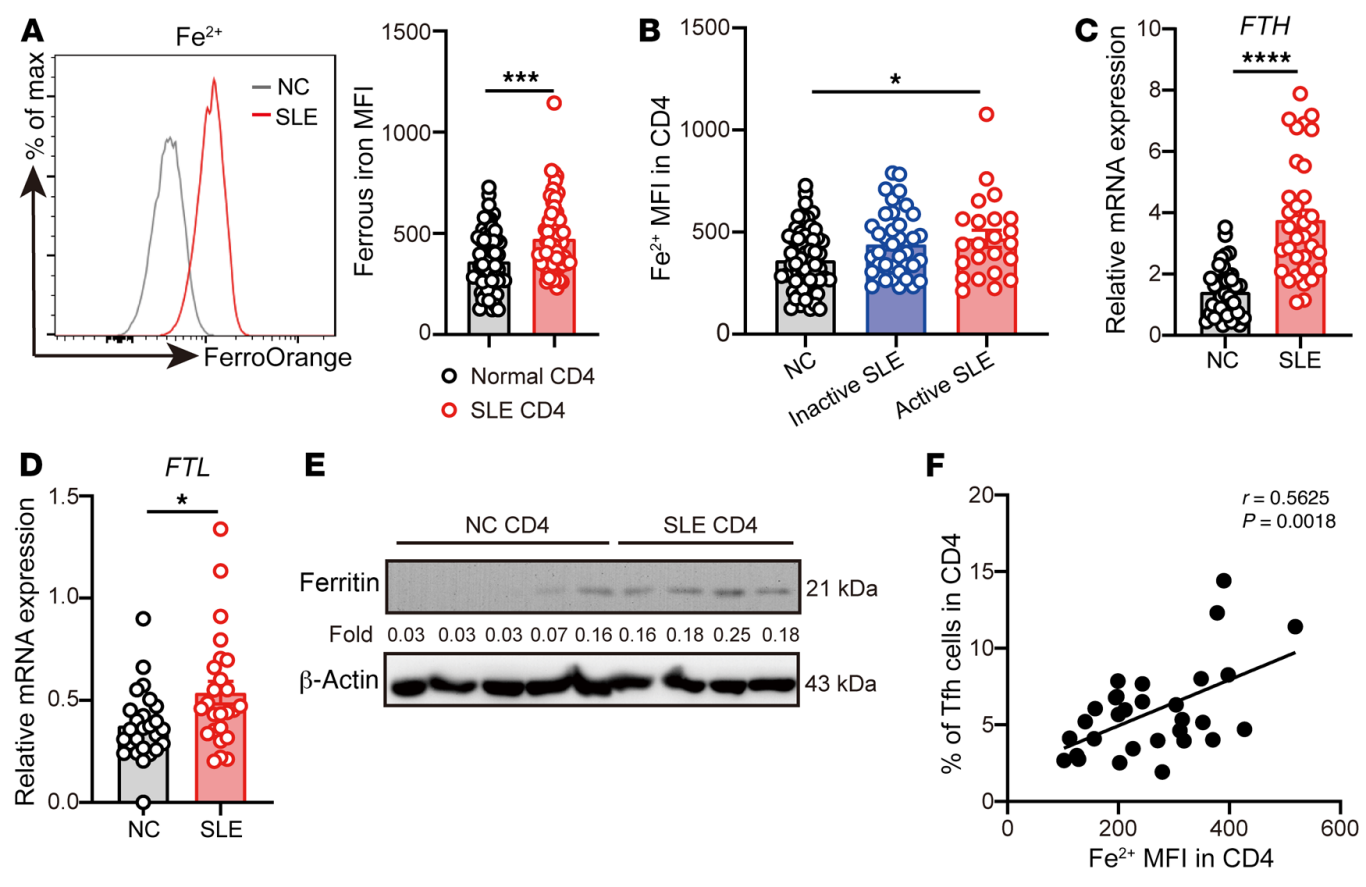
Increased intracellular iron in lupus CD4^+^ T cells. (**A**) Representative flow cytometry and quantification of Fe^2+^ in CD4^+^ T cells from healthy donors or patients with SLE (*n =* 58 for healthy donors, *n =* 61 for patients with SLE). (**B**) Quantification of Fe^2+^ in CD4^+^ T cells from healthy control (*n =* 58), inactive (SLEDAI ≤ 4, *n =* 38), and active (SLEDAI > 4, *n =* 23) patients with SLE. (**C**) qPCR of *FTH* in CD4^+^ T cells from healthy donors (*n =* 37) and patients with SLE (*n =* 34). (**D**) qPCR of *FTH* in CD4^+^ T cells from healthy donors (*n* = 26) and patients with SLE (*n* = 25). (**E**) Western blot of ferritin in CD4^+^ T cells from healthy donors (*n =* 5) and patients with SLE (*n =* 4). (**F**) Correlation between Fe^2+^ and Tfh cell percentage in SLE CD4^+^ T cells (*n =* 28). Data are shown as mean ± SEM. Data are representative of 2 independent experiments. *P < 0.05, ***P < 0.001, ****P < 0.0001 (unpaired 2-tailed Student’s *t* test for **A**, **C**, and **D**; 1-way ANOVA and Tukey’s multiple-comparisons test for **B**; and Pearson’s correlation for **F**).

First, we sought to determine the iron levels in lupus CD4^+^ T cells. Ferrous iron represents the soluble and bioavailable form of iron involved in cell metabolism (8). Therefore, we used a cell-permeable probe, FerroOrange (26, 27), to examine the level of free Fe^2+^ in patients with SLE. We observed that the levels of Fe^2+^ were highly increased in SLE CD4^+^ T cells (Figure 1A), especially in CD4^+^ T cells of active patients with SLE (Figure 1B). Furthermore, the mRNA levels of *FTH* and *FTL*, encoding the heavy chain and light chain of human ferritin, respectively (28), and the protein levels of ferritin were markedly increased in lupus CD4^+^ T cells (Figure 1, C–E).

To confirm whether the increase of iron is a feature of T cell activation in SLE, we compared the levels of iron in naive CD4^+^ T cells and effector T cell subsets between healthy donors and patients with SLE by flow cytometry. We found that not only CD4^+^ effector T cell subsets, including Th1, Th2, Th17, and Tfh cells, but also naive and memory CD4^+^ T cells, have significantly increased levels of Fe^2+^ in patients with SLE compared with healthy donors (Supplemental Figure 1, A and B; supplemental material available online with this article; https://doi.org/10.1172/JCI152345DS1). Although the level of Fe^2+^ in Tfh cells was not higher than that in other CD4^+^ effector T cells in patients with SLE (Supplemental Figure 1C), the percentage of Tfh cells in patients with SLE was positively correlated with the Fe^2+^ level in CD4^+^ T cells (Figure 1F). In addition, we also observed that Tfh cells in draining lymph nodes (dLNs) and spleens of lupus-prone mice have higher levels of Fe^2+^ compared with the activated non-Tfh cells (Supplemental Figure 2, A and B).

Interestingly, we also detected the Fe^2+^ level in peripheral helper T (Tph) cells, which share many B helper–associated functions with Tfh cells and induce B cell differentiation toward antibody-producing cells (29, 30). Our results showed that both the percentage of Tph cells and Fe^2+^ levels were increased in patients with SLE compared with healthy donors, but there was no significant correlation between the Fe^2+^ level in Tph cells and the percentage of Tph cells (Supplemental Figure 1, D–F).

### HID contributes to pathogenic T cell differentiation in lupus mice.

To investigate whether iron overload affects pathogenic T cell differentiation and the progression of lupus, we fed 3-week-old female MRL/*lpr* lupus-prone mice with a HID (500 mg/kg) for 20 weeks; age-matched female MRL/*lpr* mice fed with a normal iron diet (ND, 50 mg/kg) were served as controls. HID reduced the body weight of MRL/*lpr* mice in the last 4 weeks (Supplemental Figure 3A). The serum iron levels were increased in 23-week-old HID-treated MRL/*lpr* mice compared with the ND group (Supplemental Figure 3B). After 20 weeks of HID treatment, the proportion and number of CD4^+^ T cells were increased significantly in spleens but not in dLNs, compared with the ND-fed mice (Supplemental Figure 3, C and D). On the contrary, the proportion and number of CD8^+^ T cells were significantly reduced in HID-fed mice (Supplemental Figure 3D). These results were consistent with those of previous studies that showed that the percentage of CD8^+^ T cells was inversely correlated with iron storage (31). In addition, HID increased the proportion and number of F4/80^+^CD11b^+^ macrophages (Supplemental Figure 3E) but did not affect CD3^+^CD4^–^CD8^–^ double-negative T cells (Supplemental Figure 3D), DCs (Supplemental Figure 3F), and B220^+^ B cells (Supplemental Figure 3G).

**Figure 2:**
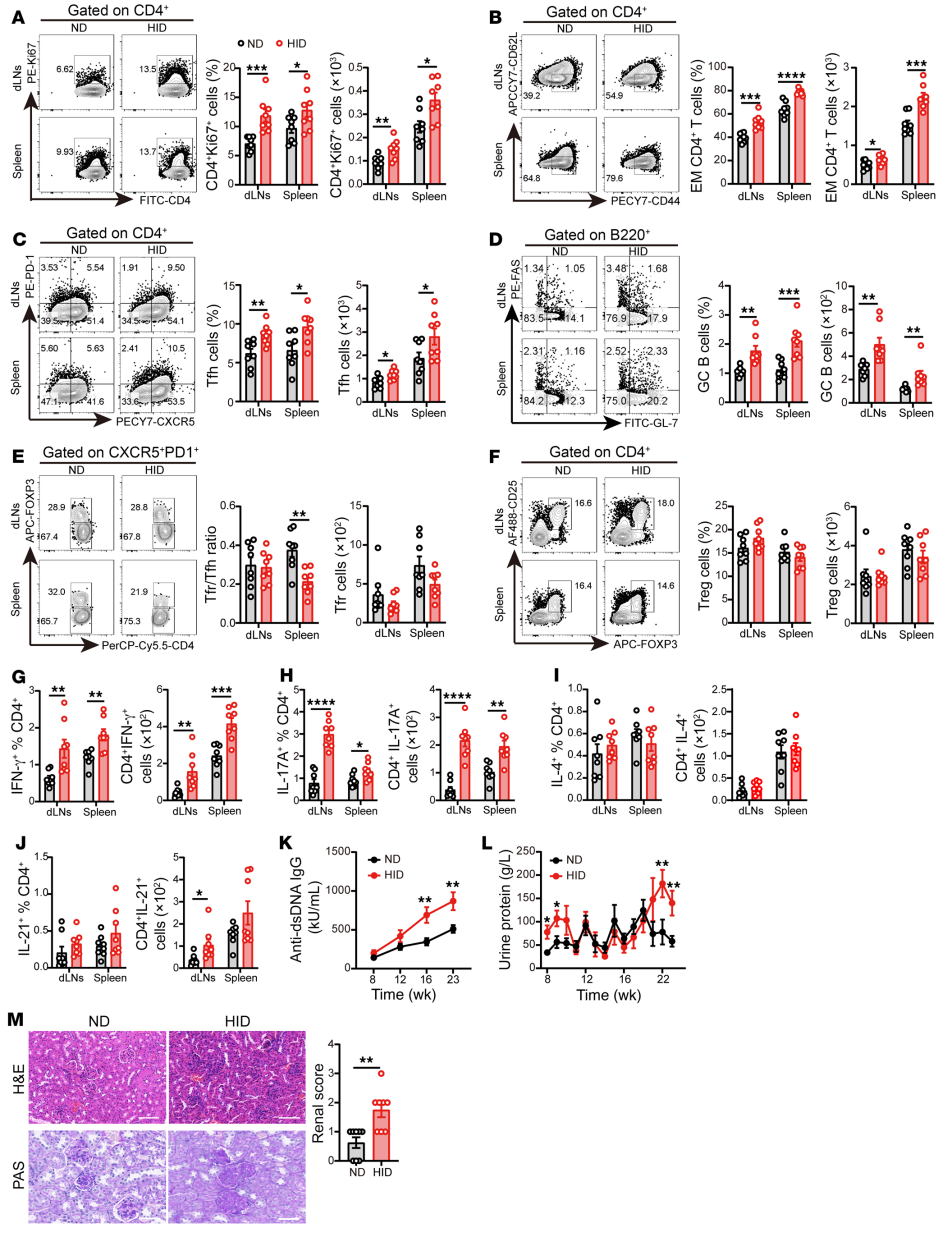
HID contributes to pathogenic T cell differentiation in lupus mice. 3-week-old female MRL/*lpr* mice were fed with a normal iron diet (ND, 50 mg/kg, *n =* 8) or a high-iron diet (HID, 500 mg/kg, *n =* 8) for 20 weeks. (**A**–**F**) Representative flow cytometry and (**A**) quantification of CD4^+^Ki67^+^ cells, (**B**) CD4^+^CD44^+^CD62L^–^ effector memory (EM) cells, (**C**) CD4^+^CXCR5^+^PD-1^+^ Tfh cells, (**D**) B220^+^GL-7^+^FAS^+^ GC B cells, (**E**) CD4^+^CXCR5^+^PD-1^+^FOXP3^+^ Tfr cells, and (**F**) CD4^+^CD25^+^FOXP3^+^ Tregs in MRL/*lpr* mice fed with ND or HID. (**G**–**J**) Quantification of (**G**) CD4^+^IFN-γ^+^ cells, (**H**) CD4^+^IL-17A^+^ cells, (**I**) CD4^+^ IL-4^+^ cells, and (**J**) CD4^+^ IL-21^+^ cells in MRL/*lpr* mice fed with ND or HID. (**K**) Serum levels of anti-dsDNA IgG in MRL/*lpr* mice fed with ND or HID. (**L**) Urine protein of MRL/*lpr* mice fed with ND or HID. (**M**) Representative morphology (by H&E and PAS staining) and histological scoring of kidneys of MRL/*lpr* mice after 20 weeks of ND or HID treatment. Scale bar: 50 μm. Cells were isolated from dLNs and spleens of 23-week-old ND- and HID-treated mice. Data are shown as mean ± SEM. Data are representative of 2 independent experiments. *P < 0.05, **P < 0.01, ***P < 0.001, ****P < 0.0001 (unpaired 2-tailed Student’s *t* test for **A**–**K** and unpaired 2-tailed Mann-Whitney U tests for **L** and **M**).

CD4^+^ T cells in HID-treated mice showed increased proliferation (Figure 2A) as well as obvious expansion of CD44^+^CD62L^–^ effector memory (EM) T cells (Figure 2B), compared with the ND-treated controls. We sought to determine which effector CD4^+^ T cell subsets were affected by HID. The results showed that HID significantly increased the percentages and numbers of Tfh cells and GC B cells in MRL/*lpr* mice (Figure 2, C and D). The size and number of GCs were also significantly increased in MRL/*lpr* mice fed with HID (Supplemental Figure 3H). Moreover, HID reduced the Tfr/Tfh cell ratio in the spleens of MRL/*lpr* mice without a significant difference in Tfr cell number (Figure 2E). The percentage and number of Tregs were not affected by HID (Figure 2F). Effector CD4^+^ T cells exert control on immune responses by cytokine secretion (32). We detected significant increases in the proportions and numbers of IFN-γ^+^CD4^+^ T cells and IL-17A^+^CD4^+^ T cells in dLNs and spleens of HID-fed mice (Figure 2, G and H). However, we did not observe significant change in IL-4 expression in CD4^+^ T cells of HID-fed mice compared with the ND group (Figure 2I and Supplemental Figure 4, A and C). Although the proportion of IL-21^+^CD4^+^ T cells was slightly elevated in the HID group, without significant difference compared with the ND group, the number of IL-21^+^CD4^+^ T cells in dLNs and the mRNA expression of *IL21* in splenic CD4^+^ T cells were increased significantly in HID-fed mice (Figure 2J and Supplemental Figure 4, B and C). HID did not affect the percentage of B220^–^CD138^+^ plasma cells, but the number of plasma cells was elevated in the spleens of HID-fed mice (Supplemental Figure 3I). Furthermore, HID elevated the serum levels of anti-dsDNA IgG in MRL/*lpr* mice (Figure 2K). In the last 3 weeks of treatment, HID significantly increased the urine protein levels in MRL/*lpr* mice (Figure 2L). The histological analysis also exhibited more serious injury and more T cell infiltration in the kidneys of HID-treated mice compared with the ND group (Figure 2M and Supplemental Figure 3J). These results suggest that HID promotes the differentiation of pathogenic Th cells, as well as CD4^+^ T cell proliferation and effector CD4^+^ T cell expansion, contributing to autoimmune responses and disease progression in lupus mice.

### HID promotes exogenous antigen-induced GC response.

To address the role of iron in T cell biology, we fed 3-week-old female C57BL/6 (B6) mice with HID for 7 weeks. HID did not alter the proportions and numbers of total CD4^+^ and CD8^+^ T cells in B6 mice (Supplemental Figure 5A). After 7 weeks of HID treatment, though the proportion and number of CD62L^+^CD44^–^ naive CD4^+^ T cells exhibited a variable response to HID (Supplemental Figure 5B), the percentage and number of EM CD4^+^ T cells were markedly increased in the spleen and dLNs of HID-treated mice (Supplemental Figure 5B), suggesting that HID promotes EM CD4^+^ T cell differentiation. Increased iron involved in ROS production can be harmful to cell viability (33). Therefore, we examined the levels of intracellular ROS in T cells. The results showed that HID increased the level of ROS in naive CD4^+^ T cells of dLNs (Supplemental Figure 5, C and D) but had no influence in EM CD4^+^ T cells (Supplemental Figure 5E).

**Figure 3:**
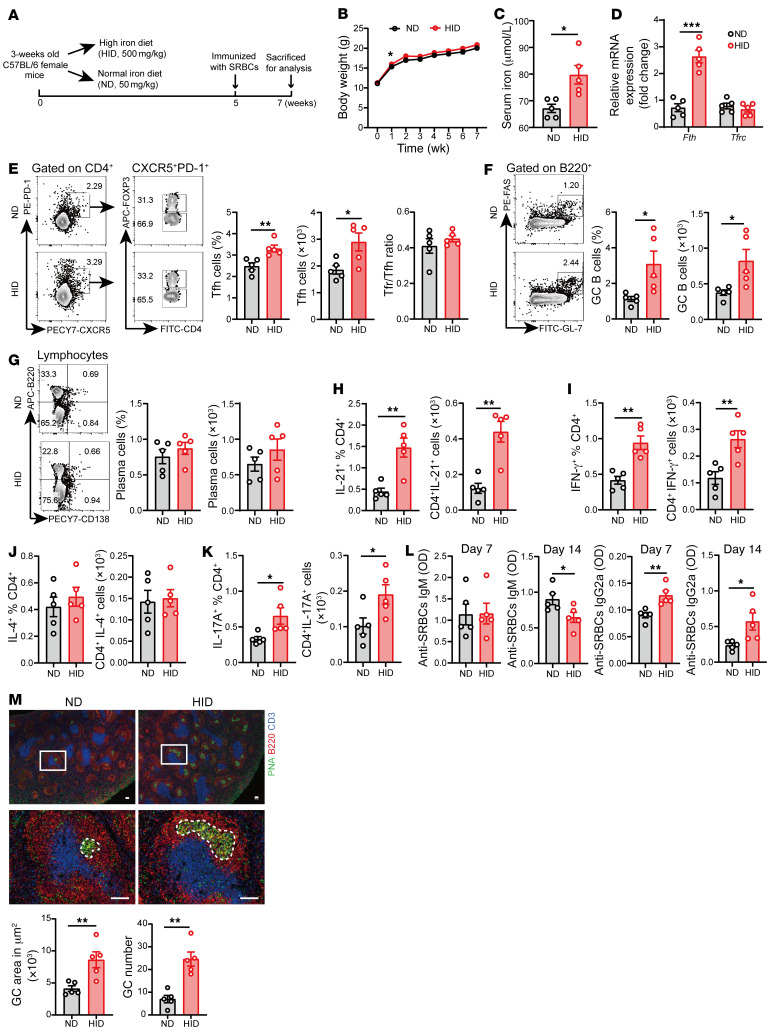
HID promotes exogenous antigen-induced GC response. 3-week-old female B6 mice were treated with a normal iron diet (ND, 50 mg/kg, *n =* 5) and a high-iron diet (HID, 500 mg/kg, *n =* 5) for 5 weeks and immunized with sheep red blood cells (SRBCs) by i.p. injection. Mice were sacrificed for analysis after 2 weeks of immunization. (**A**) Schematic diagram of the HID treatment and SRBC immunization. (**B**) Body weight change of mice treated with ND or HID. (**C**) The level of serum iron in mice treated with ND or HID. (**D**) mRNA expression of *Fth* and *Tfrc* in splenic CD4^+^ T cells of ND- or HID-treated mice. (**E**) Representative flow cytometry and quantification of CD4^+^CXCR5^+^PD-1^+^ Tfh cells and CD4^+^CXCR5^+^PD-1^+^Foxp3^+^ Tfr cells. (**F**) Representative flow cytometry and quantification of B220^+^GL-7^+^FAS^+^ GC B cells. (**G**) Representative flow cytometry and quantification of B220^–^CD138^+^ plasma cells. (**H**–**K**) Quantification of the percentage and numbers of (**H**) CD4^+^IL-21^+^ cells, (**I**) CD4^+^IFN-γ^+^ cells, (**J**) CD4^+^IL-4^+^ cells, and (**K**) CD4^+^IL-17A^+^ cells. (**L**) Serum levels of anti-SRBC IgM and anti-SRBC IgG2a in ND- and HID-treated mice at day 7 and day 14 of SRBC immunization. (**M**) Representative histology and quantification of GCs in the spleen after 2 weeks of SRBC immunization. Blue, CD3; red, B220; green, PNA. Scar bar: 100 μM. Cells were isolated from the spleens of ND- and HID-treated mice immunized with SRBCs. Data are shown as mean ± SEM. Data are representative of 2 independent experiments. *P < 0.05, **P < 0.01, ***P < 0.001 (unpaired 2-tailed Student’s *t* test for **B**–**M**).

To access the requirement of iron for T cell–dependent (TD) humoral response, we immunized the 2 groups of mice with sheep red blood cells (SRBCs) by i.p. injection after 5 weeks of HID treatment and continuously fed them with HID for 2 weeks (Figure 3A). Seven weeks of HID treatment did not affect the body weight (Figure 3B), but the level of serum iron was significantly increased (Figure 3C). We next determined the expression of iron-related genes to evaluate the conditions of intracellular iron in CD4^+^ T cells. The transcription of *Fth*, encoding the heavy chain of ferritin required for intracellular iron storage (34), can be quickly regulated by intracellular iron through the iron-responsive elements/iron-regulatory protein system (9). The expression of *Fth* was markedly elevated in splenic CD4^+^ T cells of mice with 7 weeks of HID, suggesting that the level of intracellular iron was increased in CD4^+^ T cells after 7 weeks of HID treatment (Figure 3D). However, there was no significant difference in expression of *Tfrc*, encoding transferrin responsible for iron uptake, between the HID and ND group (Figure 3D). To clarify how HID affected intracellular iron in CD4^+^ T cells, we detected the expression of *Fth*, *Tfrc*, and *Slc40a1* (encoding the ferroportin responsible for iron export) during the process of HID treatment. The results showed that the expression of *Tfrc*, *Fth* and *Slc40a1* genes was significantly increased after 2 weeks of HID feeding (Supplemental Figure 6). Then, the expression levels of *Tfrc* and *Slc40a1* were gradually reduced, and *Fth* expression still remained at a high level (Supplemental Figure 6). These results suggest that T cells maintain intracellular iron homeostasis in the iron-sufficient environment by promoting iron storage and dynamically regulating the expression of iron transport-related genes.

After 14 days of SRBC immunization, HID-treated mice showed a significant increase in percentage and numbers of Tfh cells (Figure 3E). No significant differences were observed in the Tfr/Tfh ratio (Figure 3E). HID also elevated the frequency and number of B220^+^GL-7^+^Fas^+^ GC B cells in the spleen and dLNs of HID-treated mice (Figure 3F). However, the proportion and number of B220^–^CD138^+^ plasma cells were not affected by HID (Figure 3G). Furthermore, the proportions and numbers of IFN-γ^+^, IL-17A^+^, IL-21^+^ CD4^+^ T cells were significantly increased in the spleens of HID-treated mice immunized with SBRCs, while the frequency and number of IL-4–secreting CD4^+^ T cells was not affected by HID (Figure 3, H–K). These results indicate that iron reshapes the cytokine milieu, by favoring proinflammatory cytokine production, in TD humoral response. We examined the changes of serum antibodies at day 0, day 7, and day 14 after immunization with SRBCs to evaluate the effect of iron on antigen-specific antibody production. HID significantly increased the production of anti-SRBC IgG2a but reduced the levels of anti-SRBC IgM in B6 mice (Figure 3L and Supplemental Figure 7). Increased production of IgG isotypes and reduction of antigen-specific IgM are related to the maturation of GCs (35, 36). Besides, the cytokine milieu also plays a role in the outcome of which IgG isotype gains predominance. IFN-γ promotes a IgG2a-predominant antibody response, whereas IL-4 favors a IgG1-predominant antibody response in mice (37, 38). Therefore, elevated secretion of IFN-γ might promote the production of antigen-specific IgG2a in HID-treated mice. Consistently, histological analysis also showed a stronger GC response in HID-treated mice (Figure 3M).

To confirm whether the increased humoral response from SRBC-immunized mice after HID treatment is T cell dependent, we isolated CD4^+^ T cells from ND- and HID-fed mice and mixed them well with CD19^+^ B cells isolated from ND-fed mice. T/B cell suspensions were injected into the tail veins of Rag2^–/–^ mice. After 7 days of T/B cell transfer, Rag2^–/–^ mice were immunized with SRBCs by i.p. injection. Mice were sacrificed for analysis after 7 days of SRBC immunization (Supplemental Figure 8A). The results showed that Rag2^–/–^ mice receiving HID CD4^+^ T cells had higher percentages of Tfh cells and GC B cells compared with the mice receiving ND CD4^+^ T cells (Supplemental Figure 8, B and C). Furthermore, ELISA showed that the titers of anti-SRBC IgG1, IgG2a, IgG2b, and IgM were increased significantly in the Rag2^–/–^ mice transferred with HID CD4^+^ T cells compared with that in the mice transferred with ND CD4^+^ T cells, suggesting that the increased humoral response from SRBC-immunized mice with HID is T cell dependent (Supplemental Figure 8D).

Together, these results suggest that HID promotes the expansions of Tfh and GC B cells, and the production of proinflammatory cytokines in CD4^+^ T cells, as well as the secretions of antigen-specific IgG isotypes in TD humoral response.

### Intracellular iron promotes human Tfh cell differentiation in vitro.

**Figure 4:**
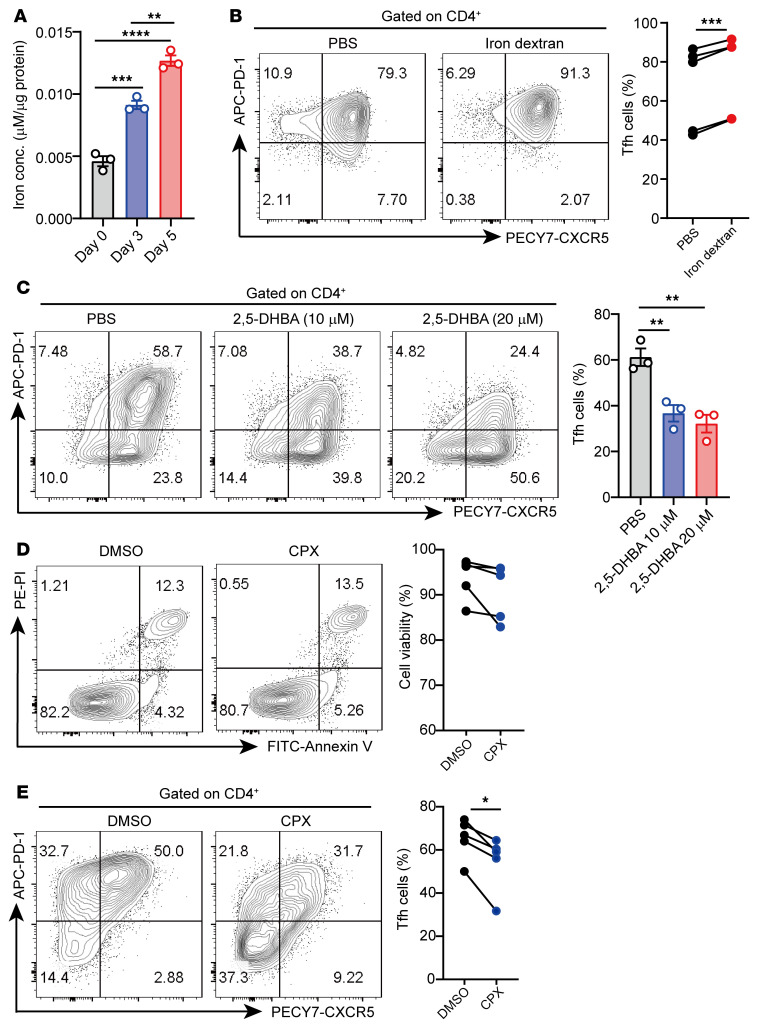
Intracellular iron promotes Tfh cell differentiation in vitro. (**A**) Quantification of intracellular iron in healthy donor naive CD4^+^ T cells cultured under Tfh cell–polarized conditions for different amounts of time (*n =* 3). (**B**) Healthy donor naive T cells were cultured under Tfh cell–polarized conditions in the presence of PBS control or iron dextran (20 μM), and the percentage and quantification of CD4^+^CXCR5^+^PD-1^+^ Tfh cells were determined by flow cytometry 3 days later (*n =* 5). (**C**) Healthy donor naive CD4^+^ T cells were cultured under Tfh cell–polarized conditions in the presence of 2,5-DHBA (10 μM and 20 μM). After 3 days of differentiation, the percentage and quantification of the CD4^+^CXCR5^+^PD-1^+^ Tfh percentage were determined (*n =* 3). (**D** and **E**) Healthy donor naive CD4^+^ T cells were cultured under Tfh cell–polarized conditions for 3 days and treated with DMSO or CPX (20 μM) for the last 4 hours. Representative flow cytometry and quantification of (**D**) viable cells and (**E**) CD4^+^CXCR5^+^PD-1^+^ Tfh cells are shown (*n =* 5). Data are shown as mean ± SEM. Data are representative of 2 independent experiments with 3–5 donors. *P < 0.05, **P < 0.01, ***P < 0.001, ****P < 0.0001 (1-way ANOVA with Tukey’s multiple-comparisons test for **A** and **C**, and paired 2-tailed Student’s *t* test for **B**, **D**, and **E**).

To investigate the role of iron in Tfh cell differentiation, we examined the changes of intracellular iron in the process of Tfh cell differentiation in vitro. We observed that intracellular iron levels were progressively elevated alongside the differentiation of Tfh cells (Figure 4A). Furthermore, iron dextran supplementation significantly enhanced Tfh cell differentiation (Figure 4B). In addition, we found the same changes, in terms of differentiation of Tfh cells, in Th1 and Th17 cells (Supplemental Figure 9, A and B and Supplemental Figure 10A). However, iron dextran supplementation did not affect the effector functions of T cell subsets under neutral conditions (only with anti-CD3/CD28 antibodies; Supplemental Figure 10B).

The mammalian siderophore 2,5-dihydroxybenzoic acid (2,5-DHBA) is a high-affinity iron-binding molecule that traffics iron from the cytoplasm to mitochondria (39). Cells lacking 2,5-DHBA accumulate high levels of cytoplasmic iron (39). We detected intracellular iron level changes in CD4^+^ T cells treated with 2,5-DHBA and an intracellular iron chelator, CPX (40). The results showed an approximately 20% decrease in intracellular iron after treatment (Supplemental Figure 11, A and B). Compared with the PBS control group, 2,5-DHBA significantly inhibited the differentiation of Tfh cells by reducing intracellular iron accumulation in CD4^+^ T cells (Figure 4C). CPX treatment for 4 hours also led to an approximately 15% reduction in Tfh cell percentage without affecting the cell viability (Figure 4, D and E). Collectively, these results indicate that intracellular iron overload enhances Tfh cell differentiation in vitro.

### miR-21 favors iron accumulation in Tfh cells.

We sought to investigate the mechanism causing iron accumulation in lupus Tfh cells. BDH2 serves as the enzyme responsible for 2,5-DHBA synthesis in mammals (39). Both in vivo and in vitro deletion of BDH2 cause intracellular iron accumulation (41, 42). Our previous work demonstrated that miR-21 targets BDH2 to promote iron accumulation in lupus CD4^+^ T cells (25). Therefore, we asked whether the same mechanism also operates in Tfh cell differentiation. We transfected naive CD4^+^ T cells with Agomir-21 to overexpress miR-21 and then cultured them in Tfh cell–polarized conditions in vitro. After 3 days of Tfh polarization, Agomir-21 increased intracellular iron levels in induced Tfh cells (Supplemental Figure 12A). Conversely, cells transfected with Antagomir-21, a specific inhibitor of miR-21, showed a reduced level of iron in induced Tfh cells (Supplemental Figure 12B). To test the role of miR-21 target gene *BDH2* in intracellular iron accumulation of Tfh cells, we transfected Tfh cells with the constructed recombinant plasmid pCMV6-BDH2 to overexpress BDH2. The result showed that pCMV6-BDH2 promoted intracellular iron accumulation in induced Tfh cells (Supplemental Figure 12C). On the contrary, inhibition of BDH2 by siRNA-BDH2 reduced the levels of intracellular iron in induced Tfh cells (Supplemental Figure 12D). These results indicate that miR-21 and BDH2 are involved in iron accumulation during Tfh cell differentiation.

### miR-21 promotes the differentiation and function of Tfh cells.

**Figure 5:**
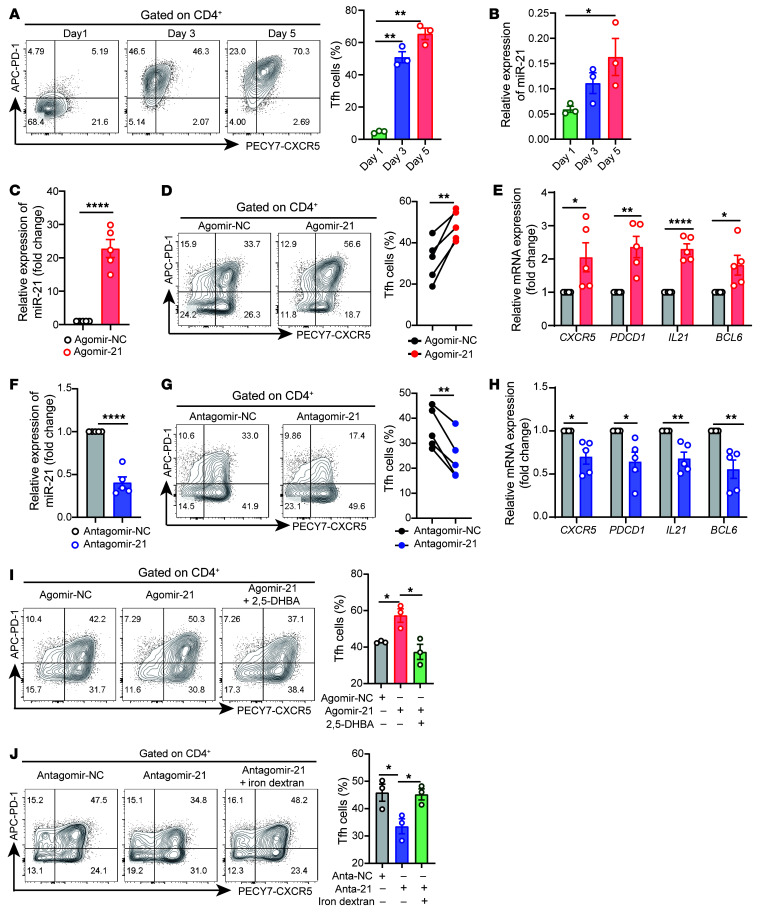
miR-21 contributes to Tfh cell differentiation in vitro. (**A**) Healthy naive CD4^+^ T cells were cultured under Tfh cell–polarized conditions for 1, 3, and 5 days. Representative flow cytometry and quantification of CD4^+^CXCR5^+^PD-1^+^ Tfh cells are shown (*n =* 3). (**B**) qPCR of miR-21 during the differentiation process of Tfh cells in **A**. (**C**–**E**) Healthy naive CD4^+^ T cells were transfected with Agomir-NC or Agomir-21 and cultured under Tfh cell–polarized conditions for 3 days (*n =* 5). After 3 days of polarization, (**C**) the expression level of miR-21, (**D**) flow cytometry and quantification of CD4^+^CXCR5^+^PD-1^+^ Tfh cells, and (**E**) mRNA expression of *CXCR5*, *PDCD1*, *IL21*, and *BCL6* were analyzed. (**F**–**H**) Healthy naive CD4^+^ T cells were transfected with Antagomir-NC or Antagomir-21 and cultured under Tfh cell–polarized conditions for 3 days (*n =* 5). After 3 days of polarization, (**F**) the expression of miR-21, (**G**) flow cytometry and quantification of CD4^+^CXCR5^+^PD-1^+^ Tfh cells, and (**H**) mRNA expression of *CXCR5*, *PDCD1*, *IL21*, and *BCL6* were analyzed. (**I**) Representative flow cytometry and quantification of CD4^+^CXCR5^+^PD-1^+^ Tfh cells transfected with Agomir-NC, Agomir-21, and Agomir-21 plus 2,5-DHBA (*n =* 3). (**J**) Representative flow cytometry and quantification of CD4^+^CXCR5^+^PD-1^+^ Tfh cells transfected with Antagomir-NC, Antagomir-21, or Antagomir-21 plus iron dextran (*n =* 3). Data are shown as mean ± SEM. Data are representative of at least 2 independent experiments with 3–5 donors. *P < 0.05, **P < 0.01, ****P < 0.0001 (1-way ANOVA with Tukey’s multiple-comparisons test for **A**, **B**, **I**, and **J** and 2-tailed Student’s *t* test for **C**–**H**).

We asked whether the expression kinetics of miR-21 overlaps with the progress of Tfh cell differentiation. Therefore, we determined the expression of miR-21 and the frequency of Tfh cells during Tfh cell differentiation progress. Results showed that the increase in Tfh cell percentage was parallel with the gradually elevated expression of miR-21 (Figure 5, A and B). A similar trend was also observed in the ex vivo differentiation process of Tfh cells of B6 mice (Supplemental Figure 13, A–C).

To confirm the role of miR-21 in Tfh cell differentiation, we transfected healthy naive CD4^+^ T cells with Agomir-21 or Agomir-NC and stimulated them to differentiate into Tfh cells in vitro. As expected, cells transfected with Agomir-21 showed increased miR-21 expression (Figure 5C). Furthermore, Agomir-21 increased the frequency of Tfh cells and the mRNA levels of *CXCR5*, *PDCD1*, *IL21*, and *BCL6* (Figure 5, D and E). Similarly to the results in humans, miR-21 also promoted murine naive CD4^+^ T cells to differentiate into Tfh cells in vitro (Supplemental Figure 13, D and E). On the contrary, inhibiting miR-21 in healthy naive CD4^+^ T cells by Antagomir-21 prevented Tfh cell differentiation in vitro (Figure 5, F–H). Similarly, we observed that miR-21 conditional KO (cKO) mice (specifically knocking out miR-21 in CD4^+^ T cells) had deficient Tfh cell differentiation capability, compared with the WT mice (Supplemental Figure 13, F and G). In addition, we also observed that miR-21 overexpression by Agomir-21 promoted the differentiation of Th17 cells, but not the differentiation of Th1 cells, Th2 cells, and Tregs, under different Th cell–polarized conditions in vitro (Supplemental Figure 14A). However, overexpressing miR-21 did not affect the effector functions of T cell subsets under neutral conditions (Supplemental Figure 14B).

Next, we compared gene expression between WT Tfh cells and miR-21 cKO Tfh cells induced in vitro by RNA-Seq. Among the approximately 1400 differently expressed genes, 686 were downregulated (Supplemental Table 4) and 727 were upregulated (Supplemental Table 5) in miR-21 cKO Tfh cells relative to their WT control cells (Supplemental Figure 13H). Several Tfh signature genes, such as *Tiam1*, *Cd200*, *Pdcd1*, *Bcl6*, *Cd28*, *Blta*, *Slamf6*, and *Pou2af1*, were significantly downregulated, while *Prdm1*, *Fasl*, and *Tbx21* were upregulated in miR-21 cKO Tfh cells (Supplemental Figure 13I). These results suggest that miR-21 promotes the differentiation of Tfh cells both in humans and mice in vitro.

To investigate whether iron depletion prevents miR-21–mediated Tfh cell differentiation, we treated the induced Tfh cells with Agomir-21 or Agomir-21 plus 2,5-DHBA. We found that 2,5-DHBA prevented the increase of Tfh cell differentiation induced by Agomir-21 to levels equivalent to those of the Agomir-NC controls (Figure 5I). Similarly, CPX treatment also counteracted the increase of Tfh cell differentiation induced by miR-21 overexpression (Supplemental Figure 15). On the contrary, iron dextran supplementation recovered the differentiation of Tfh cells inhibited by Antagomir-21 to levels equivalent to those of the Antagomir-NC controls (Figure 5J).

**Figure 6:**
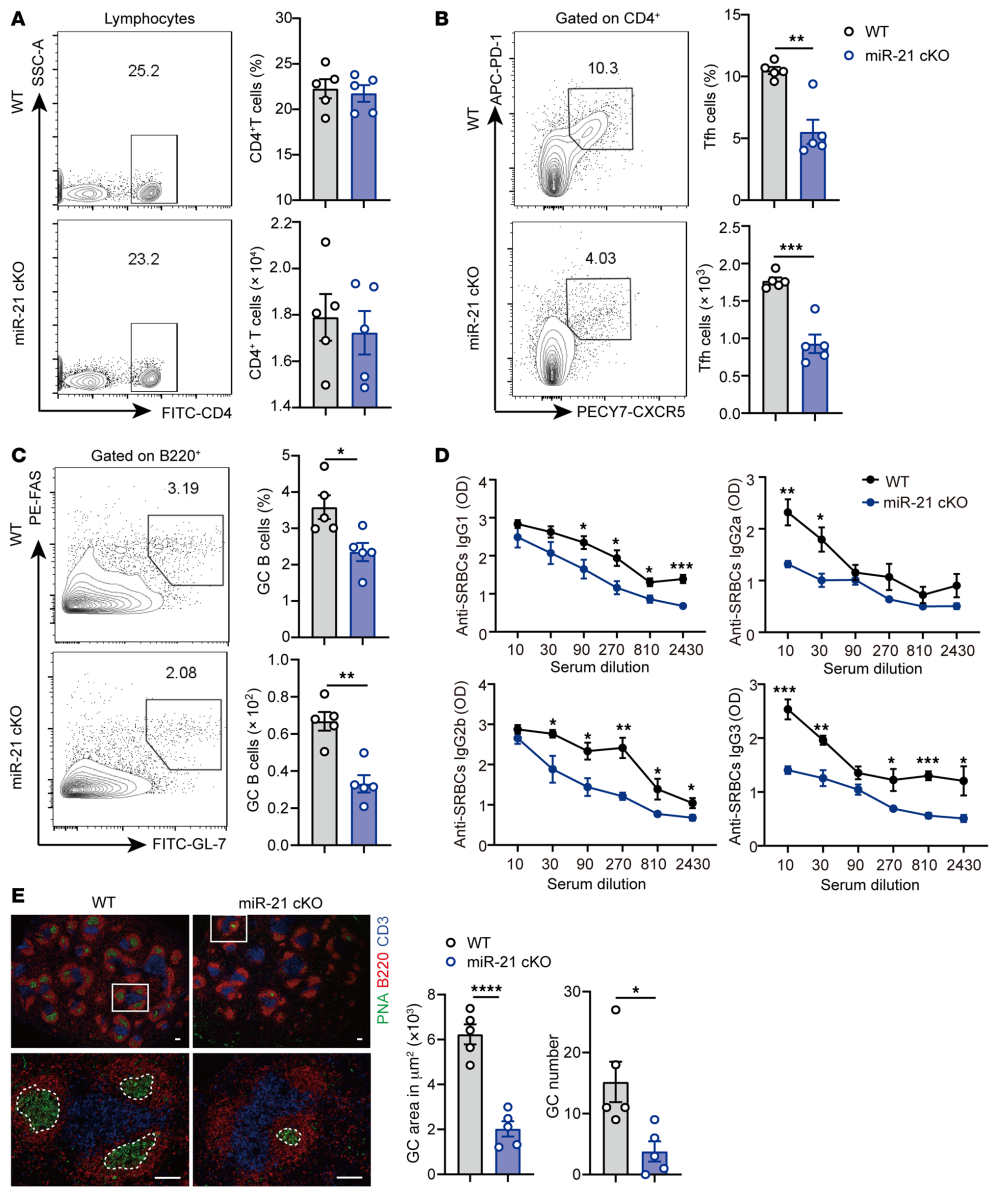
miR-21 promotes Tfh cell–mediated GC response. 8-week-old WT (*n =* 5) or miR-21 cKO mice (*n =* 5) were immunized with sheep red blood cells (SRBCs) for 7 days. After 7 days of SRBCs stimulation, mice were sacrificed for analysis. (**A**) Representative flow cytometry and quantification of CD4^+^ T cells. (**B**) Representative flow cytometry and quantification of CD4^+^CXCR5^+^PD-1^+^ Tfh cells. (**C**) Representative flow cytometry and quantification of B220^+^GL-7^+^FAS^+^ GC B cells. (**D**) Serum levels of anti-SRBC IgG isotypes after 7 days of SRBC immunization. (**E**) Representative histology of spleens at day 7 after SRBC immunization and quantification of GC number and GC area (dashed line). Blue, CD3; red, B220; green, PNA. Scale bar: 100 μM. For **A**–**C**, cells were isolated from the spleens of 8-week-old WT or miR-21 cKO mice after 7 days of SRBC immunization. Data are shown as mean ± SEM. Data are representative of 3 independent experiments. *P < 0.05, **P < 0.01, ***P < 0.001, ****P < 0.0001 (unpaired 2-tailed Student’s *t* test for **A**–**E**).

We next asked whether miR-21 affects Tfh cell–mediated humoral response in vivo. We immunized age-matched WT and miR-21 cKO mice with SRBCs to induce TD humoral response. After 7 days of immunization, mice were sacrificed for analysis. KO of miR-21 did not affect the percentage and number of total CD4^+^ T cells (Figure 6A). The percentages and numbers of Tfh cells and GC B cells were significantly decreased in the spleens of miR-21 cKO mice compared with WT controls (Figure 6, B and C). Furthermore, the serum levels of anti-SRBC IgG1, IgG2a, IgG2b, and IgG3 were markedly reduced in miR-21 cKO mice (Figure 6D). Histological analysis also confirmed an attenuated GC response in miR-21 cKO mice, as shown by the reduced size and quantities of PNA^+^ GC areas in the spleens of miR-21 cKO mice (Figure 6E).

To investigate whether HID can rescue the deficient differentiation of Tfh cells in miR-21 cKO mice, we treated 3-week-old WT mice with ND and age-matched miR-21 cKO mice with ND and HID for 5 weeks and then immunized them with SRBCs by i.p. injection. After 2 weeks of SRBC immunization, mice were sacrificed for analysis. The percentages and numbers of Tfh cells and GC B cells were markedly reduced in miR-21 cKO mice compared with those in the WT group, while miR-21 cKO mice fed with HID showed elevated percentages of Tfh cells and GC B cells compared with the miR-21 cKO mice fed with ND (Supplemental Figure 16, A and B). Furthermore, the size and number of GCs were significantly reduced in miR-21 cKO mice compared with WT mice, which were rescued by HID (Supplemental Figure 16, C and D). We collected the sera of mice at day 0, day 7, and day 14 of SRBC immunization. ELISA showed that the serum levels of anti-SRBC IgG1 and IgG2b, but not IgG3, at day 14 were significantly reduced in miR-21 cKO mice compared with the WT group, but the levels of anti-SRBC IgG1 and IgG2b were significantly enhanced in miR-21 cKO mice fed with HID (Supplemental Figure 16, E–H). These results suggest that iron supplementation rescues the defect of Tfh cell–mediated humoral response in miR-21 cKO mice.

### miR-21 promotes Tfh cell differentiation in patients with SLE and the lupus mouse model.

**Figure 7:**
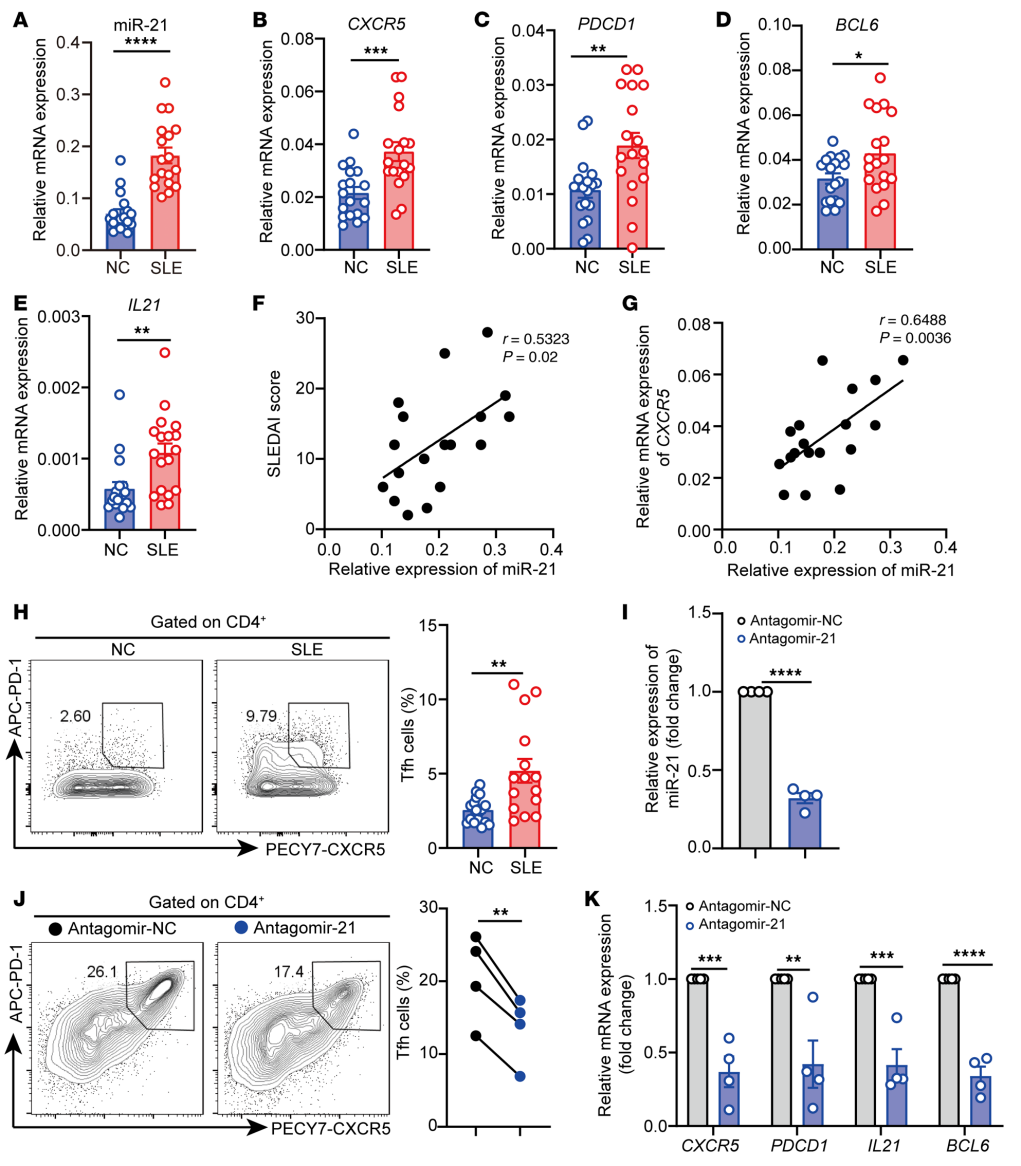
miR-21 regulates Tfh cell differentiation in lupus CD4^+^ T cells. (**A**–**E**) qPCR of (**A**) miR-21, (**B**) *CXCR5*, (**C**) *PDCD1*, (**D**) *BCL6*, and (**E**) *IL21* in CD4^+^ T cells from healthy donors and patients with SLE (*n =* 18). (**F** and **G**) Correlation between miR-21 and (**F**) SLEDAI score and (**G**) *CXCR5* mRNA in SLE CD4^+^ T cells (*n =* 18). (**H**) Representative flow cytometry and quantification of CD4^+^CXCR5^+^PD-1^+^ Tfh cells in CD4^+^ T cells isolated from peripheral blood from healthy donors (*n =* 18) and patients with SLE (*n =* 15). (**I**–**K**) SLE CD4^+^ T cells were transfected with Antagomir-NC or Anagomir-21 and stimulated by anti-CD3 and anti-CD28 for 2 days (*n =* 4). (**I**) miR-21 expression, (**J**) flow cytometry and quantification of CD4^+^CXCR5^+^PD-1^+^ Tfh cell subsets, and (**K**) mRNA levels of *CXCR5*, *PDCD1*, *IL21*, and *BCL6* were analyzed. *P < 0.05, **P < 0.01, ***P < 0.001, ****P < 0.0001 (Mann-Whitney U test for **A**, **C**, **D**, **E**, and **H** and 2-tailed Student’s *t* test for **B** and **I**–**K**; Spearman’s correlation for **F** and **G**). Data are shown as the mean ± SEM. For **I**–**K**, data are representative of 3 independent experiments with 4 donors.

We examined whether miR-21 is involved in Tfh cell differentiation in lupus. We isolated CD4^+^ T cells from the peripheral blood from healthy donors and patients with SLE to evaluate the correlation between miR-21 and Tfh cell–related genes. The expression of miR-21 was higher in CD4^+^ T cells from patients with SLE than in those from healthy controls (Figure 7A). The mRNA levels of Tfh signature genes, including *CXCR5*, *PDCD1*, *BCL6*, and *IL21*, were also increased in CD4^+^ T cells from patients with SLE (Figure 7, B–E). Furthermore, the expression of miR-21 was positively correlated with the SLE Disease Activity Index (SLEDAI) score and the mRNA level of *CXCR5* in CD4^+^ T cells from patients with SLE (Figure 7, F and G). Consistent with the mRNA expression, the frequency of CD4^+^CXCR5^+^PD-1^+^ Tfh cells was also highly elevated in peripheral blood of patients with SLE (Figure 7H).

To downregulate the expression of miR-21 in lupus CD4^+^ T cells and evaluate the effect on Tfh cell differentiation, we isolated CD4^+^ T cells from peripheral blood of patients with SLE and transfected them with Antagomir-21 and then stimulated them with anti-CD3/CD28 antibodies for two days. After two days of stimulation, Antagomir-21 reduced the expression of miR-21 (Figure 7I), the percentage of Tfh cells (Figure 7J), and mRNA levels of Tfh signature genes in lupus CD4^+^ T cells (Figure 7K). In addition, our previous study demonstrated that miR-21 promotes iron accumulation in lupus CD4^+^ T cells (25). Together, these results suggest that miR-21 favors aberrant Tfh cell expansion via increasing iron accumulation in SLE.

To further confirm the role of miR-21 in lupus, we treated the 12-week-old WT and miR-21 cKO mice with pristane by i.p. injection, which can induce a series of lupus-like symptoms in mice (43). After 12 weeks of pristane stimulation, the proportions of Tfh cells were decreased in the dLNs and spleens of miR-21 cKO mice, and the number of Tfh cells was decreased in the spleens of cKO mice (Supplemental Figure 17A). However, no significant changes were observed in Tfr/Tfh ratio (Supplemental Figure 17B). Consistent with the Tfh cells, the proportions of GC B cells were decreased in the dLNs and spleens of miR-21 cKO mice, and the cell number of GC B cells in dLNs of miR-21 cKO mice were also decreased (Supplemental Figure 17C). The serum levels of anti-dsDNA IgG and ANA total Ig were decreased in miR-21 cKO mice compared with the WT controls (Supplemental Figure 17D). Furthermore, miR-21 cKO mice exhibited alleviated urine protein in the last 4 weeks of treatment (Supplemental Figure 17E). Although we did not observe typic pathological changes of lupus nephritis due to the limitation of the observation period, morphological examination by H&E and PAS staining showed less lymphocyte infiltration and cell proliferation in the kidney of miR-21 cKO mice (Supplemental Figure 17F). Consistently, histological analysis showed that renal C3 and IgG immune complex depositions were also decreased in miR-21 cKO mice (Supplemental Figure 17G). Collectively, these data indicate that miR-21 contributes to SLE progression in human and mice.

### BDH2 is the target gene of miR-21 in regulating Tfh cells.

**Figure 8:**
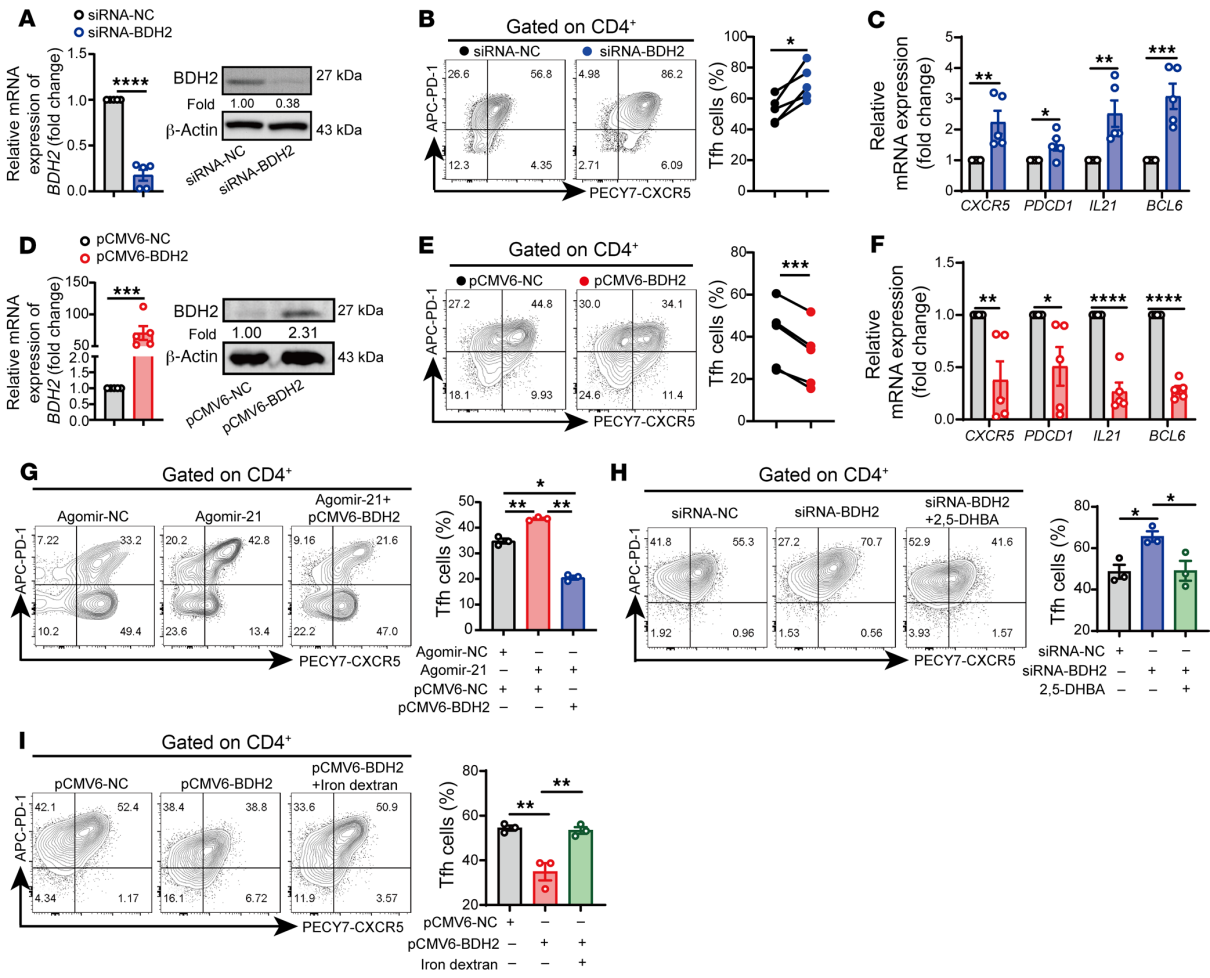
*BDH2* is the target gene of miR-21 in regulation of Tfh cells. (**A**–**C**) Healthy naive CD4^+^ T cells were transfected with siRNA-NC or siRNA-BDH2 and then cultured under Tfh cell–polarized conditions for 3 days (*n =* 5). After 3 days of stimulation, (**A**) qPCR and Western blot of BDH2, (**B**) flow cytometry and quantification of CD4^+^CXCR5^+^PD-1^+^ Tfh cells, and (**C**) qPCR of *CXCR5*, *PDCD1*, *IL21*, and *BCL6* were analyzed. (**D**–**F**) Healthy naive CD4^+^ T cells were transfected with pCMV6-NC or pCMV6-BDH2 and then cultured under Tfh cell–polarized conditions for 3 days (*n =* 5). (**D**) qPCR and Western blot of BDH2, (**E**) flow cytometry and quantification of CD4^+^CXCR5^+^PD-1^+^ Tfh cells, and (**F**) qPCR of *CXCR5*, *PDCD1*, *IL21*, and *BCL6* were analyzed. (**G**) Representative flow cytometry and quantification of induced Tfh cells in cells transfected with Agomir-NC, Agomir-21, and Agomir-21 plus pCMV6-BDH2 (*n =* 3). (**H**) Representative flow cytometry and quantification of CD4^+^CXCR5^+^PD-1^+^ Tfh cells in cells transfected with siRNA-NC, siRNA-BDH2, and siRNA-BDH2 plus 2,5-DHBA (*n =* 3). (**I**) Representative flow cytometry and quantification of CD4^+^CXCR5^+^PD-1^+^ Tfh cells in cells transfected with pCMV6-NC, pCMV6-BDH2, or pCMV6-BDH2 with iron dextran (*n =* 3). Data are shown as mean ± SEM. Data are representative of at least 2 independent experiments with 3–5 donors. *P < 0.05, **P < 0.01, ***P < 0.001, ****P < 0.0001 (2-tailed Student’s *t* test for **A**–**F** and 1-way ANOVA with Tukey’s multiple-comparisons test for **G**–**I**).

We further investigated the role of BDH2 in miR-21–mediated Tfh cell differentiation. First, we examined the expression levels of BDH2 in the process of Tfh cell differentiation. We observed a reduced expression of BDH2 during the process of Tfh cell differentiation, together with increased BCL6 and ferritin (Supplemental Figure 18, A and B). Furthermore, BDH2 was downregulated in induced Tfh cells transfected with Agomir-21 (Supplemental Figure 18, C and D), while it was increased in cells transfected with Antagomir-21 (Supplemental Figure 18, E and F). These results indicate that BDH2 might be involved in Tfh cell differentiation. Next, we examined whether changing BDH2 expression affects Tfh cell differentiation. We used siRNA-BDH2 to inhibit BDH2 expression in naive CD4^+^ T cells (Figure 8A) and then induced them to differentiate into Tfh cells. The results showed that the percentage of Tfh cells and mRNA expression of *CXCR5*, *PDCD1*, *IL21*, and *BCL6* were significantly increased in cells transfected with siRNA-BDH2 (Figure 8, B and C). On the contrary, in cells transfected with pCMV6-BDH2, the expression of BDH2 was highly increased compared with controls (Figure 8D), and the frequency of Tfh cells and mRNA levels of *CXCR5*, *PDCD1*, *IL21*, and *BCL6* were significantly decreased (Figure 8, E and F). Furthermore, rescuing the expression of BDH2 by transfecting pCMV6-BDH2 in Agomir-21–treated cells prevented Tfh cell differentiation to levels lower than in Agomir-NC controls (Figure 8G). These results suggest that *BDH2* is the target gene of miR-21 in regulating Tfh cell differentiation.

Because BDH2 plays an important role in intracellular homeostasis (39, 41, 42), we asked whether changing the iron bioavailability affects the role of BDH2 on Tfh cell differentiation. We used 2,5-DHBA to deplete intracellular iron in induced Tfh cells transfected with siRNA-BDH2 and found that iron depletion inhibited the differentiation of Tfh cells in cells downregulating BDH2 to levels comparable with the controls (Figure 8H). Conversely, iron dextran supplementation recovered the frequency of Tfh cells in cells overexpressing BDH2 to levels equivalent to the pCMV6-NC controls (Figure 8I). These results suggest that BDH2 modulates intracellular iron to affect Tfh cell differentiation.

### Inhibition of BDH2 promotes DNA hydroxymethylation of the BCL6 promoter by increasing intracellular iron.

Fe^2+^ can serve as a cofactor of several epigenetic enzymes, such as TET enzymes, to regulate immune cell biology (13, 20, 44). Iron-dependent TET enzymes catalyze 5-methylcytosine to 5-hydroxymethylcytosine, which leads to DNA hydroxymethylation and demethylation, activating gene transcription (13, 44). We asked whether the miR-21/BDH2 axis affects TET enzyme activity and then alters DNA methylation/hydroxymethylation of genes that control Tfh cell differentiation. In naive CD4^+^ T cells with TET2 or TET3 deficiency (*Tet2* cKO and *Tet3* cKO), overexpression of miR-21 did not affect the differentiation of Tfh cells (Supplemental Figure 19, A and B), suggesting that TET enzymes are required for miR-21 to regulate Tfh cell differentiation.

**Figure 9:**
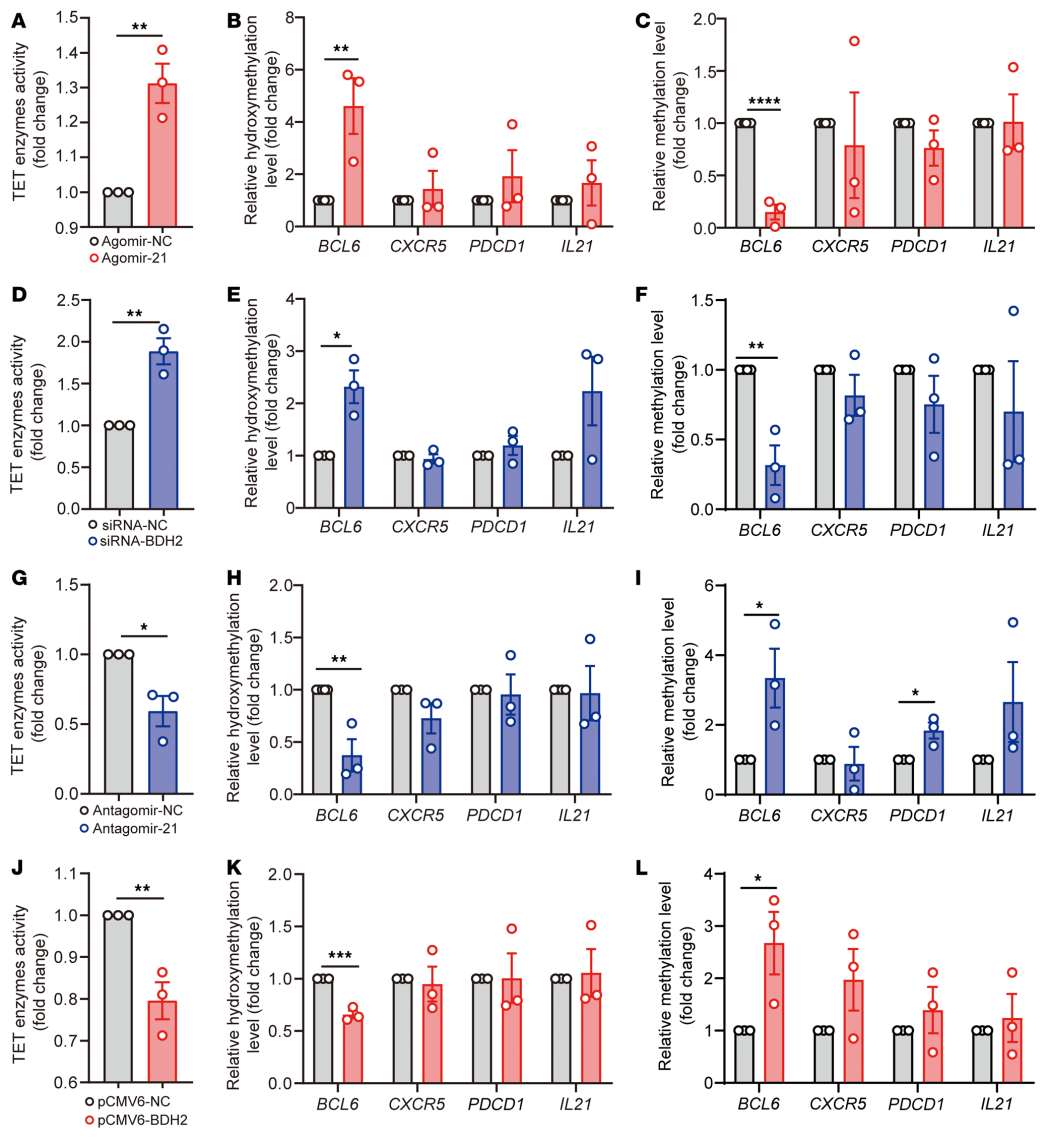
The miR-21/BDH2 axis promotes DNA hydroxymethylation of the *BCL6* gene by regulating intracellular iron. Healthy naive CD4^+^ T cells were isolated from peripheral blood from healthy donors and cultured under Tfh cell–polarized conditions for 3 days. After 3 days of stimulation, cells were collected for analysis. (**A**) Activity of TET enzymes, and (**B**) relative hydroxymethylation and (**C**) methylation levels in the promoter of Tfh cell–related genes *BCL6*, *CXCR5*, *PDCD1*, and *IL21* in induced Tfh cells transfected with Agomir-NC or Agomir-21 (*n =* 3). (**D**) Activity of TET enzymes, and (**E**) relative hydroxymethylation and (**F**) methylation levels in the promoter of *BCL6*, *CXCR5*, *PDCD1*, and *IL21* in induced human Tfh cells transfected with siRNA-NC or siRNA-BDH2 (*n =* 3). (**G**) Activity of TET enzymes, and (**H**) relative hydroxymethylation and (**I**) methylation levels in the promoter of *BCL6*, *CXCR5*, *PDCD1*, and *IL21* in induced human Tfh cells transfected with Antagomir-NC or Antagomir-21 (*n =* 3). (**J**) Activity of TET enzymes, and (**K**) relative hydroxymethylation and (**L**) methylation levels in the promoter of *BCL6*, *CXCR5*, *PDCD1*, and *IL21* in induced human Tfh cells transfected with pCMV6-NC or pCMV6-BDH2 (*n =* 3). Data are shown as mean ± SEM. Data are representative of 3 independent experiments with 3 donors. *P < 0.05, **P < 0.01, ***P < 0.001, ****P < 0.0001 (2-tailed Student’s *t* test for **A**–**L**).

We next examined the TET enzyme activity in cells transfected with Agomir-21 or siRNA-BDH2. Indeed, we observed increased TET enzyme activity in induced Tfh cells overexpressing miR-21 or downregulating BDH2 (Figure 9, A and D), but we saw no significant changes in TET2/TET3 expression (Supplemental Figure 19, C–F). We then performed methylated DNA immunoprecipitation (MeDIP) and hydroxymethylated DNA immunoprecipitation–qPCR (hMeDIP-qPCR) to determine the effect of miR-21/BDH2 on DNA methylation/hydroxymethylation of Tfh signature genes. In cells transfected with Agomir-21 or siRNA-BDH2, we observed increased DNA hydroxymethylation and decreased DNA methylation in the *BCL6* gene promoter, but no significant differences were detected in promoter regions of *CXCR5*, *PDCD1*, and *IL21* compared with their corresponding controls (Figure 9, B, C, E, and F). On the contrary, inhibiting miR-21 by Antagomir-21 or overexpressing BDH2 by pCMV6-BDH2 reduced TET enzyme activity (Figure 9, G and J), but the expression of TET2 and TET3 was not affected compared with the control groups (Supplemental Figure 19, G–J). We observed decreased DNA hydroxymethylation and increased DNA methylation in the *BCL6* gene promoter of cells inhibiting miR-21 or overexpressing BDH2 (Figure 9, H, I, K, and L). Besides, DNA methylation in the *PDCD1* gene promoter was also increased in cells inhibiting miR-21 (Figure 9I). However, there were no significant differences in DNA methylation/hydroxymethylation of *CXCR5* and *IL21* gene promoters (Figure 9, H, I, K, and L). The changes in the genomic distribution of 5-methylcytosine and 5-hydroxymethylcytosine in the *BCL6* promoter were also verified using MeDIP-Seq and hMeDIP-Seq in Tfh cells transfected with Agomir-21 or siRNA-BDH2 (Supplemental Figure 20). We detected another Fe^2+^-dependent epigenetic enzyme, JMJD3, in Tfh cells; it is responsible for the demethylation of H3K27me3 during T cell differentiation (45). However, JMJD3 enzyme activity was not affected by miR-21/BDH2 in Tfh cell differentiation, (Supplemental Figure 21). These results suggest that the miR-21/BDH2/intracellular iron axis promotes Tfh cell differentiation via inducing TET enzyme–mediated DNA hydroxymethylation of the *BCL6* promoter.

## Discussion

Iron overload has been reported in murine and human lupus, but the pathogenic mechanism is poorly understood (16, 17, 46). We showed here that intracellular iron was increased in lupus CD4^+^ T cells (Figure 1). Iron overload promoted pathogenic T cell differentiation, accelerating the disease progression of lupus mice. Notably, iron overload favored the expansion of spontaneous Tfh cells and GC B cells, as well as the proinflammatory cytokine-producing CD4^+^ T cells, aggravating autoantibody production and disease progression of MRL/*lpr* lupus-prone mice (Figure 2). In addition to driving pathogenic T cell differentiation in lupus, iron overload also augmented the expansion of Tfh cells and GC B cells, and the secretion of proinflammatory cytokines, as well as the production of antigen-specific IgG isotypes in TD humoral response (Figure 3). This study extends our knowledge about the role of iron in autoimmune diseases and humoral immunity and highlights that maintaining iron homeostasis might be critical for eliminating pathogenic Th cells and balancing the protective humoral response with autoimmune response.

Tfh cells have emerged as a central player in SLE pathogenesis (47, 48). Some evidence has suggested that the proportions of Tfh or Tfh-like cells were increased in patients with SLE (49, 50). Therefore, there is a great need for understanding the mechanism causing aberrant Tfh cell differentiation in lupus. Here, we showed that iron was required for Tfh cell differentiation. HID increased the proportion of Tfh cells and enhanced GC response in mice (Figures 2 and 3). 2,5-DHBA, which is catalyzed by BDH2, serves as a siderophore to reduce cytoplasmic iron (39, 42). Treatment with 2,5-DHBA or iron chelator CPX significantly inhibited the differentiation of Tfh cells by decreasing intracellular iron (Figure 4, C–E, and Supplemental Figure 11). Furthermore, the iron levels in CD4^+^ T cells from patients with SLE were positively correlated with the percentage of Tfh cells (Figure 1F). This finding indicates that, in patients with SLE, iron overload plays a positive role in Tfh cell differentiation. Further investigation of iron metabolism in Tfh cells will develop the understanding of the mechanism that causes aberrant Tfh cell differentiation and function in SLE.

The trace element iron is capable of affecting effector T cell activation and function. A previous study showed that iron promotes proinflammatory cytokine GM-CSF and IL-2 expression in T cells by regulating the stability of an RNA-binding protein, PCBP1 (12). Iron uptake blockade in autoreactive T cells inhibits their capability to induce experimental autoimmune encephalomyelitis. In addition, tetrahydrobiopterin (BH4) production in activated T cells is related to changes in iron metabolism and mitochondrial bioenergetics, and blockading BH4 synthesis improved T cell–mediated autoimmunity and allergic inflammation (51). These studies indicate that iron metabolism plays an important role in T cell functions and autoimmune diseases. However, the role and mechanism of iron in regulating T cell activation, differentiation, and autoantibody production in autoimmune diseases, such as SLE, remain unclear. Here, our data showed that iron overload enhanced Tfh cell differentiation and GC response as well as increased Th1 and Th17 cell differentiation, which promoted antibody production and autoimmune response in SLE. These data confirm the important role of iron in the pathogenesis of SLE.

Previous studies have demonstrated that miR-21 promotes T cell activation and apoptosis (52, 53). Furthermore, CD4^+^ T cells with miR-21 overexpression acquire increased capacities to support B cell maturation and Ig production, which is similar to the function of Tfh cells, indicating that miR-21 might be related to Tfh cell differentiation (54). Our finding reveals a potentially novel role of miR-21 in regulating Tfh cell differentiation and GC response via modulating intracellular iron homeostasis. We have reported that miR-21 targets BDH2 to catalyze intracellular iron accumulation in human and murine CD4^+^ T cells (25). We showed here that, in both humans and mice, miR-21 was capable of promoting Tfh cell differentiation (Figure 5 and Supplemental Figure 13). In SRBC-induced TD humoral response, miR-21 promoted GC formation and antigen-specific antibody production (Figure 6). miR-21 was also involved in aberrant Tfh cell expansion in patients with SLE (Figure 7). Furthermore, the deletion of miR-21 in CD4^+^ T cells alleviated the disease progression of pristane-induced lupus in mice (Supplemental Figure 17). On the contrary, BDH2 inhibited Tfh cell differentiation (Figure 8). The miR-21/BDH2 axis modulated iron homeostasis in Tfh cells (Supplemental Figure 12). Modulating intracellular iron recovered effects of the miR-21/BDH2 axis on Tfh cell differentiation (Figure 5, I and J; Supplemental Figure 15; and Figure 8, H and I). These observations indicate the potentially novel function of miR-21 in regulating Tfh cell differentiation, which further confirms that miR-21 is the key target for SLE therapy.

DNA methylation is involved in regulating the plasticity of CD4^+^ T cell differentiation (23). Several lineage-determining transcription factors, such as FOXP3 and RORC, can be affected by DNA methylation during Th cell differentiation (55, 56). TET enzymes catalyze DNA hydroxymethylation to promote gene expression, and this process requires Fe^2+^ to serve as a cofactor (20). A body of evidence has demonstrated that DNA demethylation contributes to T cell overactivation in SLE (57–59). In addition, our previous studies have also shown that iron overload enhances DNA hydroxymethylation and demethylation, promoting gene transcription and CD4^+^ T cell overactivation in SLE (25). Here, we demonstrated that miR-21–mediated Tfh cell differentiation is dependent on TET enzymes. miR-21/BDH2/intracellular iron axis affects TET enzyme activity in Tfh cells (Figure 9). Previous studies have reported that *BCL6* gene is sensitive to DNA methylation (60, 61). Using MeDIP and hMeDIP techniques, we found that the promoter region of *BCL6* gene was sensitive to miR-21/BDH2 axis–mediated DNA methylation/hydromethylation (Figure 9 and Supplemental Figure 20). Our results demonstrated the iron can control cell differentiation by inducing TET enzyme–mediated DNA demethylation of key genes, and iron overload is an important manipulator for aberrant epigenetic modifications occurring in SLE CD4^+^ T cells.

**Figure 10:**
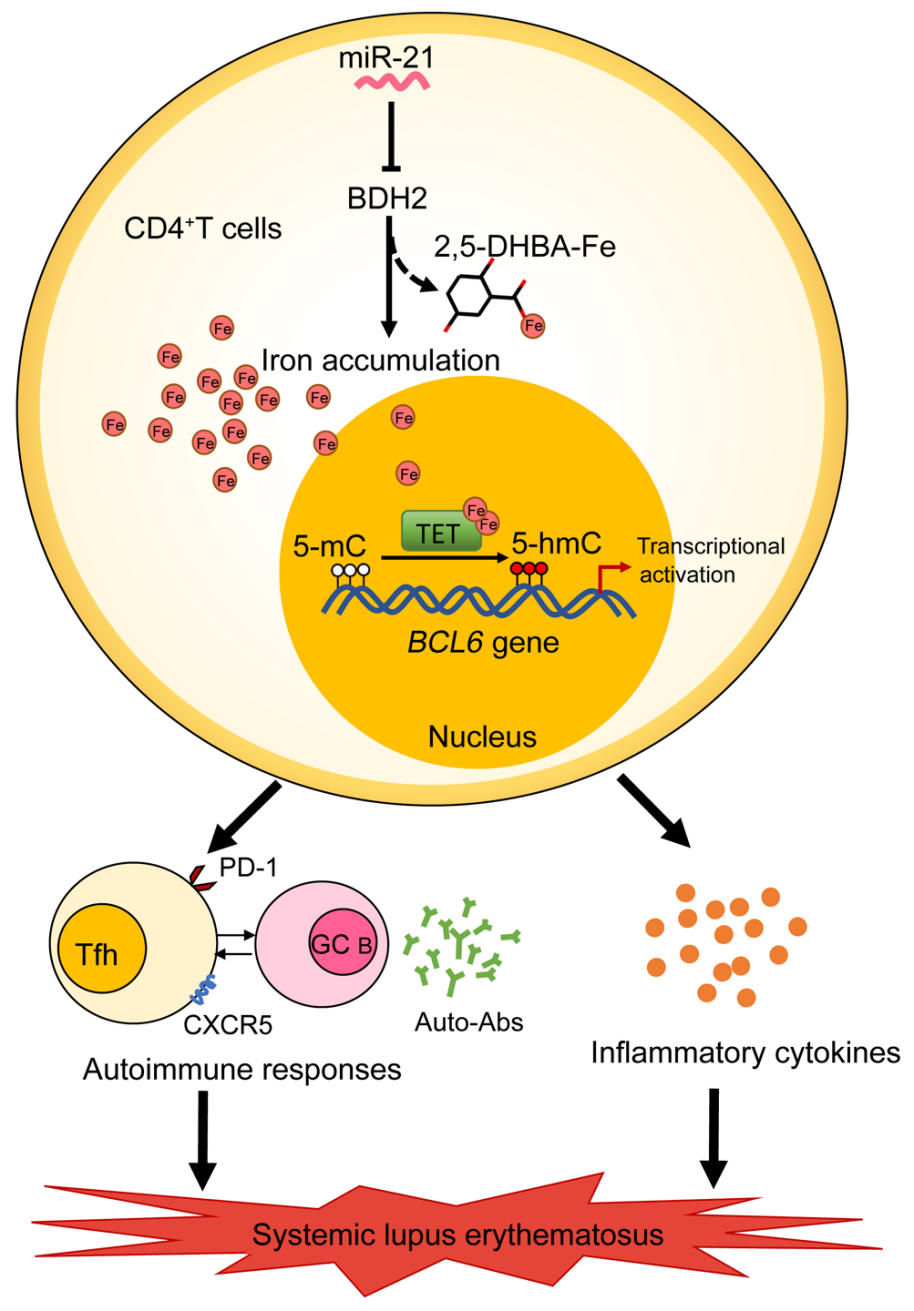
Schematic illustration of the contribution of iron overload to the pathogenic T cell differentiation and pathogenesis of SLE. In lupus CD4^+^ T cells, iron accumulation promotes Tfh cell differentiation and Tfh cell–mediated autoimmune responses, autoantibody production, as well as inflammatory cytokine secretion, driving disease progression of lupus. Mechanically, miR-21 represses BDH2 to induce iron accumulation in lupus CD4^+^ T cells by limiting the synthesis of siderophore 2,5-DHBA, which enhances Fe^2+^-dependent TET enzyme activity and promotes *BCL6* promoter hydroxymethylation and transcription activation, leading to excessive Tfh cell differentiation in SLE. Together, iron overload is an important inducer of the autoimmune response in lupus, and maintaining iron homeostasis will provide a good way for therapy and management of SLE.

In summary, our study shows that iron overload is an important inducer of the autoimmune response in lupus and indicates that regulating iron homeostasis may be a good target for SLE therapy. Our study also provides the experimental basis for the importance of controlling dietary iron in clinical management for patients with SLE. We demonstrate a potentially novel mechanism, in which miR-21 overexpression enhances Fe^2+^-dependent TET enzyme activity through repression of BDH2 and promotes *BCL6* promoter hydroxymethylation and transcription activation, leading to excessive Tfh cell differentiation in SLE; this mechanism provides potentially novel strategies for SLE therapy (Figure 10). Further study of iron-based treatment for SLE in mouse models and clinic experiments will be needed.

## Methods

Further information can be found in Supplemental Methods.

### Patients.

193 patients with SLE were recruited from outpatient dermatology clinics and in-patient wards, and all of them met at least 4 of the American College of Rheumatology Revised Criteria (62). The SLEDAI was used to assess lupus disease activity. Activity categories were evaluated based on the SLEDAI score: inactive (SLEDAI ≤ 4), active (SLEDAI > 4). Age-, sex-, and ethnicity-matched healthy donors were enrolled by medical staff at the Second Xiangya Hospital. Patient information is listed in Supplemental Table 1.

### Mice and HID treatment.

B6 and MRL/*lpr* mice were purchased from Slack Company. For the HID lupus mouse model, 3-week-old female MRL/*lpr* mice were fed with a HID (500 mg/kg) for 20 weeks. Age-matched MRL/*lpr* mice receiving a ND (50 mg/kg) for 20 weeks served as controls. The ingredients of the chow used in the HID group were the same as those in chow used in the ND group, except for iron content. Serum was collected monthly from 8 weeks of age onward to detect the anti-dsDNA IgGs in MRL/*lpr* mice. Urine protein was assessed using a colorimetric assay strip (URIT). For miR-21 cKO mice generation, the loxP-miR-21-loxP mice were constructed by Shanghai Biomodel Organism Science & Technology Development Co. Ltd. *Cd4-cre* mice (stock no. 022071) were purchased from Shanghai Biomodel Organism Science & Technology Development Co. Ltd. The miR-21–floxed allele (WT) mice were bred with *Cd4-cre* transgenic mice to generate miR-21^fl/fl^ *Cd4-cre* (miR-21 cKO) mice. *Tet2^fl/fl^* and *Tet3^fl/fl^* mice were provided by Akihiko Yoshimura at the Department of Microbiology and Immunology, Keio University School of Medicine, Tokyo, Japan (63). We generated *Tet2^fl/fl^*;*Cd4-cre* (*Tet2* cKO) and *Tet3^fl/fl^*;*Cd4-cre* (*Tet3* cKO) mice by crossing *Tet2^fl/fl^* and *Tet3^fl/fl^* mice with *Cd4-cre* mice. All mice were raised in specific pathogen–free conditions.

### SRBC immunization and ELISA.

For HID feeding and SRBC immunization, 3-week-old B6 mice were pretreated with HID for 5 weeks and then were i.p. immunized with 5% SRBCs in Alsever’s solution. To analyze the role of miR-21 on TD humoral response, 8-week-old miR-21 cKO mice and WT controls were injected i.p. with 5% SRBCs in Alsever’s solution. After 7–14 days of immunization, sera were collected by cardiac puncture after anesthetizing mice, and splenic cells were stained with fluorochrome-labeled antibodies and analyzed with FlowJo software. Anti-SRBC IgG isotypes were measured by ELISA. For anti-SRBC antibody analysis, wells were coated with SRBC membrane protein (20 μg/mL) (Bersee) overnight at 4°C. Wells were blocked with 5% BSA for 1 hour and then incubated with diluted serum for 2 hours at room temperature, followed by incubation with anti-mouse IgG1 (1:3000) (Bethyl Laboratories), IgG2a (1:5000) (Bethyl Laboratories), IgG2b (1:5000) (Bethyl Laboratories), IgG3 (1:3000) (Bethyl Laboratories), and anti-mouse IgM (1:3000) (SouthernBiotech). For anti-dsDNA IgG detection, the mouse anti-dsDNA IgG ELISA kit (Alpha Diagnostic) was used according to the manufacturer’s protocols. Reagents are listed in Supplemental Table 2.

### Histology.

Mouse kidney and spleen tissues were fixed in formalin and embedded in paraffin. H&E staining was used to assess the histological features of the kidney. The renal pathology was scored according to the criteria of previous studies (64). To assess the immune complex deposition in the kidney, we stained paraffin-embedded renal sections with rabbit anti-C3 antibody (Abcam) and HRP-conjugated anti-rabbit antibody (Abcam) for mouse C3 and HRP-conjugated anti-mouse IgG antibody (Abcam) for mouse IgG. PNA (GC zone), CD3 (T cell zone), and B220 (B cell zone) were used to determine the GC area. For PNA staining, spleen tissues were stained with biotinylated anti-peanut agglutinin (Vector Laboratories ), and then incubated with biotinylated anti-peanut agglutinin antibody (Vector Laboratories). For CD3 staining, rabbit anti-CD3 antibody (Abcam) and HRP-conjugated anti-rabbit antibody (Abcam) were used. For B220 (CD45R) staining, rat anti-mouse CD45R antibody (Abcam) and HRP-conjugated goat anti-rat IgG(H+L) antibody (Proteintech) were used. After incubation of primary antibody and HRP-conjugated antibody, the opal 7-Color Manual IHC Kit (Perkin Elmer) was used for fluorescence labeling. Images were captured by Perkin Elmer, and the images were analyzed by the Mantra system. Information on antibodies is provided in Supplemental Table 2.

### Transfection of Agomir, Antagomir, siRNA, and plasmid.

For Agomir and Antagomir transfection, CD4^+^ T cells or naive CD4^+^ T cells were cultured under Opti-MEM (Gibco) with Agomir-21/Antagomir-21 (400 nM) or their corresponding controls for 6 hours. Then, RPMI 1640 complete medium (Gibco) was added at 200 nM to the final concentrations of Agomir-21/Antagomir-21 or their controls. For siRNA and plasmid transfection, naive CD4^+^ T cells were transfected with siRNA or plasmid using the Human T Cell Nucleofector Kit and Amaxa Nucleofector system (Lonza) according to the manufacturer’s protocols. Briefly, naive CD4^+^ T cells were collected and resuspended in 100 μL transfection reagents, and 10 μL siRNA (20 μM) or 5 μg plasmid was added and transfected into the cells by electroporation using the nucleofector program V-024 in the Amaxa Nucleofector apparatus (Lonza). After being cultured under RPMI 1640 complete medium (Gibco) for 6 hours, the cells were transferred to fresh complete medium and incubated for 48 to 72 hours and then harvested for subsequent experiments.

### In vitro human CD4^+^ T cell activation and Tfh cell differentiation.

For human CD4^+^ T cell activation, PBMCs were separated from peripheral blood by density gradient centrifugation (GE Healthcare). Total CD4^+^ T cells were isolated from PBMCs by positive selection using human CD4 microbeads (Miltenyi Biotec) and then cultured under the stimulation of plate-bound anti-CD3 (2 μg/mL) (Calbiochem) and anti-CD28 (1 μg/mL) (Calbiochem) for two days. For human Tfh cell differentiation, naive CD4^+^ T cells were isolated from PBMCs by negative selection using the human Naive CD4^+^ T Cell Isolation Kit (Miltenyi Biotec), and then cells were cultured under the Tfh cell–polarized conditions as previously described (48). The purity of CD4^+^ T cells and naive CD4^+^ T cells (CD4^+^CD45RA^+^) was over 95%. Briefly, naive CD4^+^ T cells were stimulated with plate-bound anti-CD3 (2 μg/mL) and anti-CD28 (1 μg/mL) plus TGF-β (5 ng/mL) (R&D System), IL-6 (20 ng/mL) (PeproTech), IL-12 (10 ng/mL) (PeproTech), and IL-21 (20 ng/mL) (R&D System) for 3 to 5 days to induce Tfh cells. The medium was refreshed on day 3.

### In vitro iron depletion and iron supplementation.

For iron dextran supplementation, naive CD4^+^ T cells were isolated from peripheral blood from healthy donors and treated with iron dextran (20 μM) (MilliporeSigma) and cultured under Tfh cell–polarized conditions for 3 days. For 2,5-DHBA treatment, naive CD4^+^ T cells were cultured under Tfh cell–polarized conditions for 3 days and treated with 2,5-DHBA (20 μM) (Selleck) for the last two days. For CPX treatment, naive CD4^+^ T cells were cultured under Tfh cell–polarized conditions for 3 days and treated with CPX (20 μM) (Selleck) for the last 4 hours.

### Flow cytometry.

The expression of cytokines, surface markers, and transcriptional factors was determined by flow cytometry using FACS Canto II (BD Biosciences) or CYTEK NL-3000 (CYTEK Biosciences), and the data were analyzed by the Flowjo software (Tree Star). In brief, for surface markers, cells were incubated with fluorochrome-labeled antibodies against surface markers at 4°C for 30 minutes protected from light. For cytokines, cells were stimulated with PMA and ionomycin with the addition of GolgiPlug (BD Biosciences) at 37°C and 5% CO_2_ for 6 hours. For intracellular staining, cells were fixed and permeabilized using the Cytofix/Cytoperm Fixation/Permeabilization Solution Kit (BD Biosciences) or Foxp3/Transcription Factor Staining Buffer Set (eBioscience) and stained with fluorescent antibodies for an additional 30 minutes at 4°C in the dark. The Annexin V Apoptosis Detection Kit I (BD Biosciences) was used according to the manufacturer’s instructions to determine cell viability, and annexin V^–^PI^–^ cells were determined as viable cells. For ROS detection, cells were loaded with DCFH-DA (10 μmol/L) (Beyotime), induced at 37°C for 30 minutes, and then washed twice with 1×PBS. For Fe^2+^ detection, FerroOrange (1 μmol/L) (DOJINDO) was loaded into cells, and cells were stained at 37°C for 30 minutes. Information on antibodies is shown in Supplemental Table 2.

### Western blotting.

Cells were lysed with radio immunoprecipitation assay buffer (MilliporeSigma) supplemented with phenylmethanesulfonyl fluoride (Beyotime). Total protein was quantified with the Pierce BCA Protein Assay Kit (Thermo Fisher Scientific). Equal amounts of proteins were separated by SDS-PAGE using 8% polyacrylamide gels and then transferred to PVDF membranes (MilliporeSigma). The membranes were blocked with 5% nonfat dry milk in PBS containing 0.1% Tween-20 (PBST) buffer for 1 hour at room temperature and then incubated with the following antibodies: anti-Ferritin (1:1000) (Abcam), anti-BDH2 (1:1000) (Proteintech), anti-BCL6 (1:1000) (CST), anti–β-actin (1:5000) (Proteintech), and anti-LaminB1 (1:1000) (CST). Band intensity was analyzed by ImageQuant LAS 4000 mini (GE-Healthcare). Quantification of BDH2 was normalized to β-actin by densitometry using ImageJ software (NIH). Information on antibodies is shown in Supplemental Table 2.

### RNA isolation and qPCR.

Total RNA was extracted from cells using TRIzol reagent (MRC), and the RNA quality was evaluated with a NanoDrop spectrophotometer (ND-2000, Thermo Fisher Scientific). Extracted RNA was reverse transcribed to generate cDNA using the PrimeScript RT reagent Kit with gDNA Eraser (TaKaRa) according to the manufacturer’s instructions. miRs were specifically reverse transcribed using specific miR primers, along with U6. The transcripts were analyzed for the expression of various genes using the LightCycler 96 (Roche) thermocycler. The relative expression levels of miRs and genes were calculated by the ΔC_t_ or ΔΔC_t_ method, which normalized to the reference gene U6 (miR) or *ACTB* (gene). Primers for miR-21 and U6 were purchased from RiboBio Co. Ltd., and other relevant primers are listed in Supplemental Table 3.

### Data availability.

All sequencing data were deposited in the Gene Expression Omnibus database (GEO GSE194050).

### Statistics.

Statistical analyses were performed with SPSS25 software. All data are presented as the mean ± SEM. Statistical significance was assessed using a 2-tailed Student’s *t* test for comparisons between 2 groups. When the sample data are not normally distributed or displayed unequal variances between 2 groups, the 2-tailed Mann-Whitney *U* test was used for statistical analysis. One-way ANOVA with relevant post hoc tests was used for multiple comparisons. When the sample data were not normally distributed or displayed unequal variances between multiple groups, the Kruskal-Wallis test and Dunn’s multiple-comparisons test were used for multiple comparisons. Pearson’s correlation was used for the correlation analysis. When the sample data were not normally distributed, Spearman’s correlation was used for the correlation analysis. *P* < 0.05 was considered significant.

### Study approval.

All human studies were approved by the Ethics Committee of the Second Xiangya Hospital of Central South University. All participants provided written informed consent. All animal procedures were approved by the Animal Care and Use Committee of the Laboratory Animal Research Center at the Second Xiangya Medical School, Central South University.

## Author contributions

XG and YS conceptualized the studies and wrote the manuscript. XG, YS, and JW analyzed the data and interpreted the results. XG, YS, JW, SL, XM, LL, LH, M Zheng, PD, YY, HL, and SJ performed the experiments. HW and DY provided suggestions for the studies. M Zhao and QL conceived the studies, interpreted the data, directed the studies, and revised the manuscript. XG and YS share co–first authorship, and the order in which they are listed was determined by their workload.

## Supplementary Material

Supplemental data

## Version 1 05/02/2022

Electronic publication
