# Supplementary material for: Iron-dependent epigenetic modulation promotes pathogenic T cell differentiation in lupus
Source: J Clin Invest. 2022 May 2;132(9):e152345. doi: 10.1172/JCI152345 (PMC9057600; doi:10.1172/JCI152345)
Supplement: Supplemental data [file jci-132-152345-s190.pdf]

Supplementary Data

Methods

Genotype identification of cKO mice

We confirmed the genotypes of WT and miR-21 cKO mice by PCR analysis of genomic DNA isolated from mouse tails using the following primers: P1 and P2 were used to identify the genotypes of the miR-21 floxed allele (982bp). P1, 5’-CAGAATTGCCCAGGCTTTTA-3’; P2, 5’- AATCCATGAGGCAAGGTGAC-3’. P3 and P4 were used to identify the genotype of the cre allele (100bp). P3, 5’-GCGGTCTGGCAGTAAAAACTATC-3’; P4, 5′-GTGAAACAGCATTGCTGTCACTT-3′.

Pristane-induced lupus mouse model

For lupus-like disease induction, 12 weeks old female WT and miR-21 cKO mice were intraperitoneally injected with 500 μL of pristane (Sigma). Urine protein was detected weekly during the observation period of pristane stimulation. Mice were sacrificed for analysis after 3 months of treatment. Cells isolated from dLNs and spleen were stained with fluorochrome-labeled antibodies and analyzed with FlowJo software. Serum was collected for autoantibody detection using mouse anti-dsDNA IgG ELISA kit (Alpha diagnostic) and mouse Anti-Nuclear Antibodies Total Ig ELISA Kit (Alpha diagnostic). Renal tissue was fixed in formalin and embedded in paraffin for histological analysis.

In vitro human Th1 and Th17 differentiation

To induce Th cell differentiation, human naive $CD4^+$ T cells were isolated from the peripheral blood of healthy donors using human naive $CD4^+$ T Cell Isolation Kit (Miltenyi Biotec). Purity of

naive $CD4^+$ T ($CD4^+CD45RA^+$) cells was over 95%. Naive $CD4^+$ T cells were stimulated with plate-bound anti-CD3 (2 μg/mL) (Calbiochem) and anti-CD28 (1 μg/mL) (Calbiochem) under different Th cell-polarizated conditions. For Th1 cell differentiation, anti-IL-4 (10 μg/mL) (eBioscience) plus IL-2 (5 ng/mL) (PeproTech), IL-12 (10 ng/mL) (PeproTech). For Th17 cell differentiation, anti-IFN-γ (10 μg/mL) (PeproTech), anti-IL-4 (10 μg/mL) (eBioscience) plus cytokines TGF-β (5 ng/mL) (R&D Systems), IL-6 (25 ng/mL) (PeproTech), IL-1β (12.5 ng/mL) (PeproTech), and IL-23 (25 ng/mL) (PeproTech). After 3-5 days of stimulation, cells were collected for analysis.

In vitro murine $CD4^+$ T cell activation and Th cell differentiation

For murine $CD4^+$ T cell activation, $CD4^+$ T cells were isolated from the splenic cell suspension of B6 mice using mouse CD4 microbeads (Miltenyi Biotec), and then cultured under the presence of plate-bound anti-CD3 (5 μg/mL) (eBioscience) and anti-CD28 (2 μg/mL) (eBioscience) for 2 days. To induce Th cell differentiation, naive $CD4^+$ T cells were isolated for the splenic cell suspension of B6 mice using mouse naive $CD4^+$ T Cell Isolation Kit (Miltenyi Biotec). Purity of naive $CD4^+$ T cells ($CD4^+CD62L^+$) cells was over 95%. Naive $CD4^+$ T cells were stimulated with plate-bound anti-CD3 (5 μg/mL) and anti-CD28 (2 μg/mL) under different Th cell polarization conditions. For Th1 differentiation, naive $CD4^+$ T cells were stimulated with anti-IL-4 (10 μg/mL) (eBioscience) plus IL-12 (10 ng/mL) (PeproTech). For Th2 differentiation, naive $CD4^+$ T cells were stimulated with anti-IFN-γ (20 μg/mL) (eBioscience) plus IL-4 (20 ng/mL) (PeproTech). For Th17 differentiation, naive $CD4^+$ T cells were stimulated with anti-IFN-γ (10 μg/mL) and anti-IL-4 (10 μg/mL) plus cytokines TGF-β (2 ng/mL) (R&D Systems), IL-6 (30 ng/mL) (PeproTech), IL-1β (10 ng/mL) (PeproTech), and IL-23 (20 ng/mL) (R&D systems). For Treg differentiation, naive $CD4^+$ T cells were stimulated with anti-IFN-γ (5 μg/mL) and anti-IL-4 (5 μg/mL) plus cytokines TGF-β (5 ng/mL) and IL-2 (10 ng/mL) (PeproTech). For Tfh differentiation, naive $CD4^+$ T cells

were stimulated with anti-IFN-γ (10 μg/mL), anti-IL-4 (10 μg/mL), anti-TGF-β (20 μg/mL) plus cytokines IL-6 (10 ng/mL) and IL-21(10 ng/mL) (R&D Systems). Cells were cultured at 37 ℃ and 5% CO2 conditions for 3-5 days. The medium was refreshed on day 3. The percentages of in vitro-stimulated Th1, Th2, Th17, Treg, and Tfh cells were determined by flow cytometry.

Adoptive transfer of T cells and B cells into Rag2$^{-/-}$ mice

Rag2$^{-/-}$ mice were purchased from Cavins Laboratory Animal Co. Ltd of Changzhou (Changzhou, China). CD4$^+$ T cells were isolated from the ND-fed B6 mice or the HID-fed B6 mice using the mouse CD4 MicroBeads (Miltenyi), and CD19$^+$ B cells were then obtained from the ND-fed B6 mice using the mouse CD19 MicroBeads (Miltenyi). The purity of CD4$^+$ T cells and CD19$^+$ B cells is determined by flow cytometry and the positive cells were over 95%. Rag2$^{-/-}$ mice were randomly divided into two groups. One group received CD4$^+$ T cells and CD19$^+$ B cells isolated from ND-fed mice and the other group was transferred with CD4$^+$ T cells isolated from HID-fed mice and CD19$^+$ B cells isolated from the ND-fed mice by tail vein injection. After 7 days of cell injection, Rag2$^{-/-}$ mice were immunized with 500 μL 5% SRBCs by intraperitoneal injection. Mice were sacrificed for analysis after 7 days of SRBCs immunization. The percentages of Tfh cells and GC B cells weres determined by flow cytometry and serum was collected to detect the titers of anti-SRBCs antibodies.

RNA-sequencing and bioinformatic analysis.

For Tfh cell preparation, splenic naive CD4$^+$ T cells were isolated from the WT and miR-21 cKO mice, and then cultured under the Tfh-polarized conditions for 5 days. Total RNA was extracted from Tfh cells using TRIzol reagent (MRC) according to the manufacturer's protocol and then submitted to SHBIO Co., Ltd. for RNA-sequencing, including total RNA sample detection, mRNA enrichment, synthesis of double-stranded cDNAs, End Repair/dA-Tailing Module, fragment

selection, and PCR amplification, library detection, and Illumina sequencing. The differentially expressed genes between the two groups were filtered by P-value < 0.05 and the absolute value of fold change > 1.2. Sequencing data are available at the NCBI Gene Expression Omnibus (GEO) under accession number GSE194050.

.

Intracellular iron detection

We used an iron colorimetric assay kit (Abcam) to determine the concentration of intracellular iron. In short, naive $CD4^+$ T cells or polarized Tfh cells were lysed by assay buffer and then centrifuged at 16,000g to remove the insoluble materials. The supernatants were collected and quantified for ferrous iron according to the manufacturer's instructions. The concentration of ferrous iron was normalized to the concentration of proteins in supernatants.

TET enzyme activity

TET enzyme activity was determined using TET Activity Assay Kit (Epigentek). Briefly, cells were harvested and washed once with 1×PBS, and the nuclear extraction was prepared using the Nuclear and Cytoplasmic Protein Extraction Kit (Beyotime). 10 μg of nuclear extracts were used for TET activity detection according to the manufacturer's instructions.

DNA extract, MeDIP and hMeDIP

Genomic DNA was extracted from cells using DNeasy Blood & Tissue Kit (Qiagen). MeDIP and hMeDIP experiments were performed using MeDIP (Active Motif) and hMeDIP kits (Active Motif). Briefly, 20 μg of purified genomic DNA was broken into 200-600 bp nucleotides by sonicator. 500 ng fragmented genomic DNA was used to perform MeDIP and hMeDIP reactions following the protocols provided by the manufacturer. 10% of fragmented genomic DNA was set as Input DNA. Immunoprecipitated DNA was assessed using real-time PCR. For the MeDIP and

hMeDIP assay, input DNAs were used to produce a standard curve at 1, 2, 4, 8, 16 ng/μL in triplicate, we used the CT value of the samples to extrapolate the concentration of the sample DNA by the standard curve plot, and then we multiply the DNA concentration by the volume of enriched DNA to determine the total amount of enriched sample DNA. Real-time PCR primer sequences are shown in Supplemental Table 3.

JMJD3 enzyme activity

JMJD3 enzyme activity was determined using JMJD3 Activity Assay Kit (Epigentek). Briefly, cells were harvested and washed once with 1×PBS, and the nuclear extraction was prepared using the Nuclear and Cytoplasmic Protein Extraction Kit (Beyotime). 10 μg of nuclear extracts were used for JMJD3 activity detection according to the manufacturer's instructions.

MeDIP and hMeDIP-sequencing and data analyses

DNA was isolated from induced Tfh cells. 1μg of original genomic DNA was fragmented to a mean size of 300 bp. The methylated DNA fractions were immunoprecipitated using the Magnetic Methylated DNA Immunoprecipitation Kit (Diagenode) according to the instructions. The hydroxymethylated DNA fractions were immunoprecipitated using the hMeDIP kit (Active Motif) according to the instructions. After that, the immunoprecipitated products were then treated with protease K and purified with ZYMO DNA Clean & Concentrator-5 (ZYMO). Finally, the MeDIP-seq and hMeDIP-seq libraries were constructed by PCR amplification with the enriched DNA as templates. The PCR products were purified using Agencourt Ampure Beads (Beckman Counlter). After analyzing by the Bioanalyzer analysis system (Agilent, Santa Clara, USA) and quantified by the real-time PCR, the library was sequenced using Illumina platform with PE150 read length at the E-GENE Institute (Shenzhen, China). The Integrative Genomics Viewer (IGV) software was used for data visualization of the MeDIP-seq and hMeDIP-seq. Raw and processed sequencing

data are available at the NCBI Gene Expression Omnibus (GEO) under accession number GSE194050.

Supplemental Figures

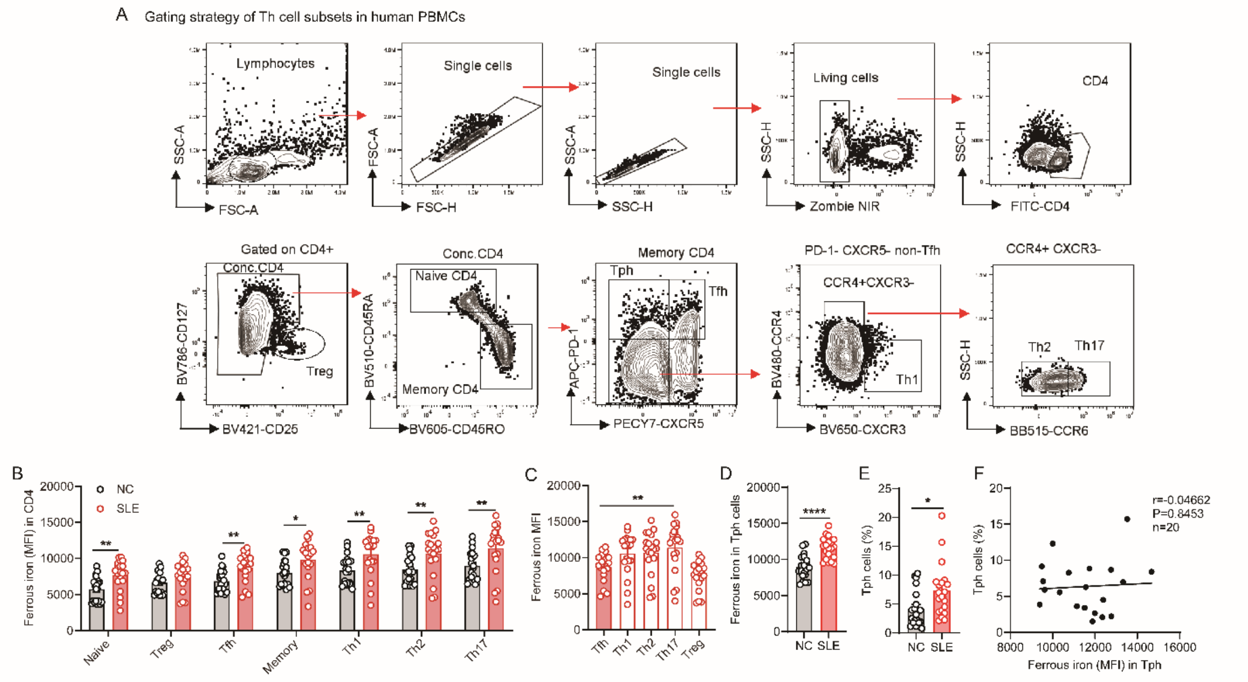

Supplemental Figure 1. The level of Fe^{2+} in $CD4^+$ T cell subsets of human blood. (**A**) Gating strategy of Th cell subsets in human PBMCs. Naive $CD4^+$ T cells were defined as $CD4^+CD45RA^+CD45RO^-$ cells, memory $CD4^+$ T cells were defined as $CD25^{-/low}$ $CD4^+CD45RO^+CD45RA^-$ cells, Th1 cells were defined as $CD25^{-/low}$ $CD4^+$ $CD45RO^+CD45RA^-$ $CXCR3^+CCR6^-CCR4^-$ cells, Th2 cells were defined as $CD25^{-/low}CD4^+$ $CD45RO^+CD45RA^-$ $CCR4^+CXCR3^-CCR6^-$ cells, Th17 cells were defined as $CD25^{-/low}CD4^+CD45RO^+CD45RA^-$ $CCR6^+CXCR3^-CCR4^+$ cells, Treg cells were defined as $CD4^+CD25^+CD127^-$ cells, Tfh cells were defined as $CD4^+CD45RO^+CD45RA^-CXCR5^+PD\text{-}1^+$ cells, Tph cells were defined as $CD4^+CD45RO^+CD45RA^-CXCR5^-PD\text{-}1^+$ cells. (**B**) Quantification of ferrous iron in Th cell subsets of healthy donors (n=20) and SLE patients (n=20). (**C**) Comparisons of ferrous iron between Tfh cells and Th1, Th2, Th17, and Treg cells in SLE patients (n=20). (**D**) Quantification of ferrous iron in circulating Tph cells of healthy donors (n=20) and SLE patients (n=20). (**E**) Quantification of Tph cell percentage in $CD4^+$T cells of healthy donors (n=20) and SLE patients (n=20). (**F**) Correlation between Fe^{2+} in Tph cells and the percentage of Tph cells in $CD4^+$ T cells. Data are shown as mean ± SEM. Data are representative of 2 independent experiments. ****$P < 0.0001$, ***$P < 0.001$, **$P < 0.01$,*$P < 0.05$ (Mann-Whitney *U* test for naive, Treg, Memory, Th1, Th2, and Th17 cells in **B** and **E**; unpaired two-tailed Student's *t*-test for Tfh cells in **B** and **D**; Kruskal-Wallis test and Dunn's multiple comparisons test for **C**; Spearman correlation for **F**).

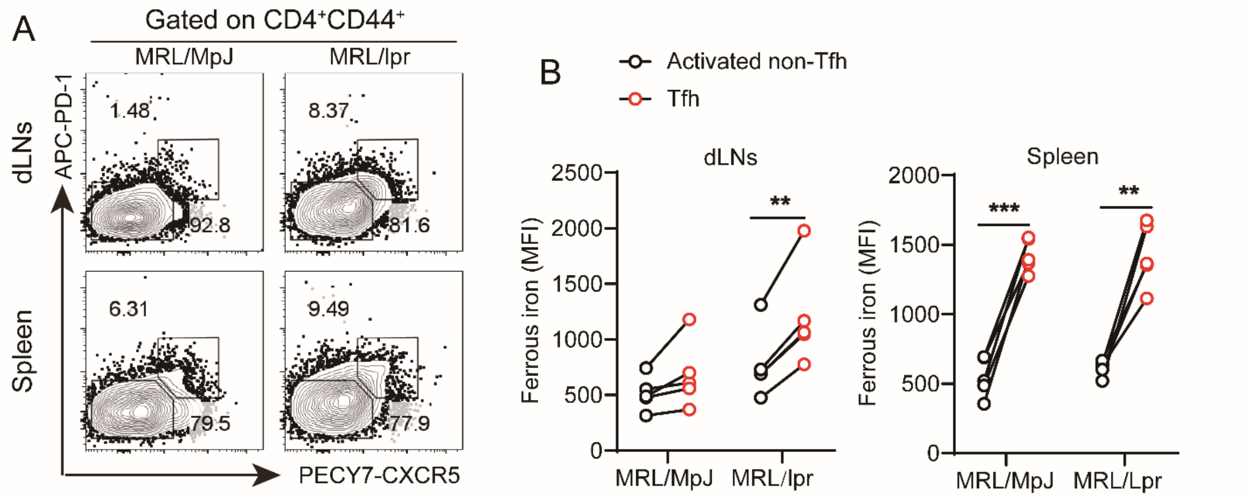

Supplemental Figure 2. Comparison of Fe^{2+} in Tfh cells and activated non-Tfh cells between MRL/MpJ and MRL/lpr mice. (**A**) Representative flow cytometry of $CD4^+CD44^+CXCR5^+PD\text{-}1^+$ Tfh cells and $CD4^+CD44^+CXCR5^-PD\text{-}1^-$ activated non-Tfh cells in draining lymph nodes (dLNs) and spleen of MRL/MpJ and MRL/Lpr mice. (**B**) Comparison of Fe^{2+} in $CD4^+CD44^+CXCR5^+PD\text{-}1^+$ Tfh cells and $CD4^+CD44^+CXCR5^-PD\text{-}1^-$ activated non-Tfh cells between aged-matched (8-weeks) MRL/MpJ and MRL/Lpr mice (n=5). Data are shown as mean ± SEM. Data are representative of 2 independent experiments. $^{**}P < 0.01$, $^{*}P < 0.05$ (paired two-tailed Student's *t*-test for **B**).

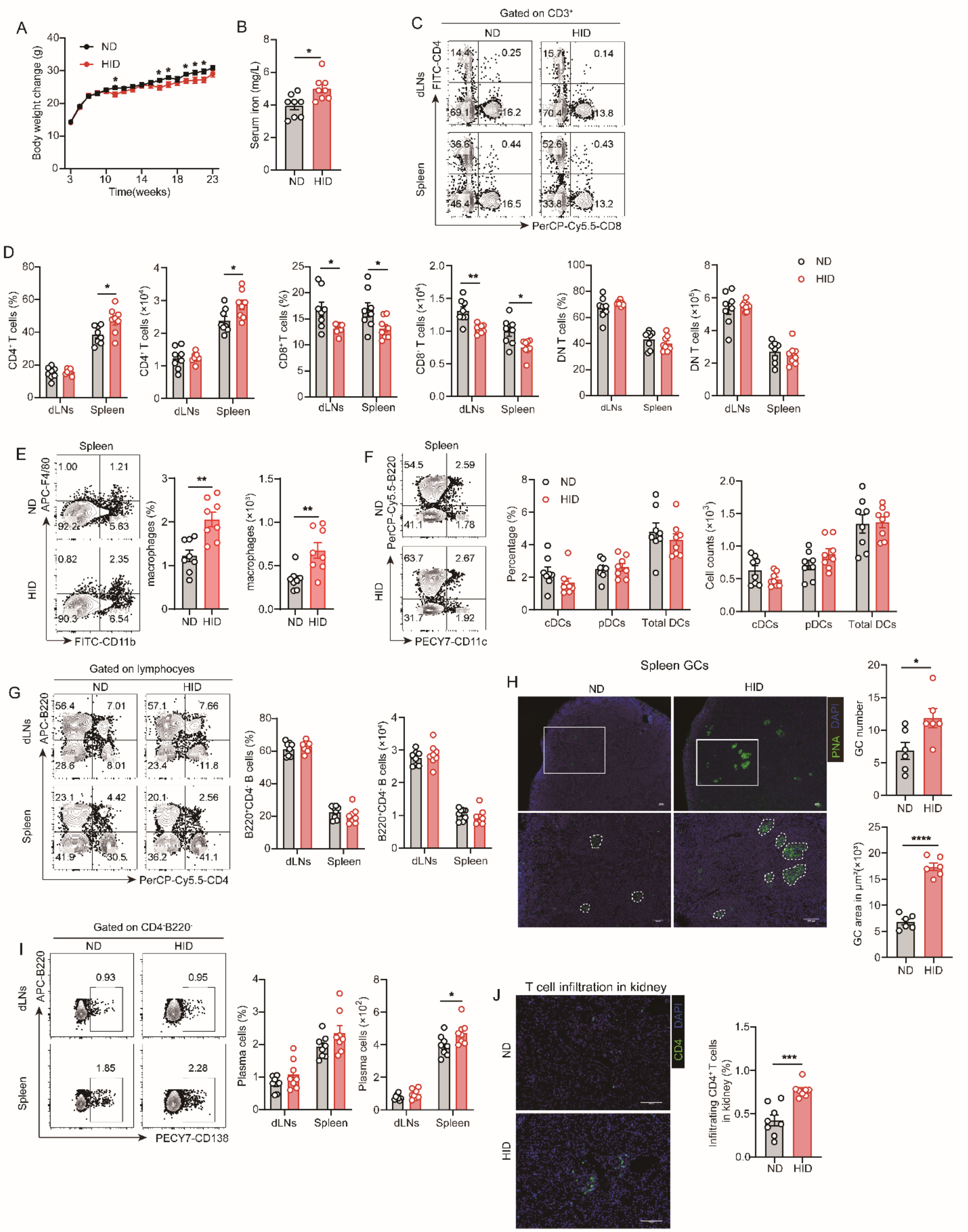

Supplemental Figure 3. Phenotypic observation of HID-treated MRL/lpr mice. 3-weeks old female MRL/lpr mice were fed with normal iron diet (ND, 50 mg/kg) or high iron diet (HID, 500 mg/kg) for 20 weeks. (**A**) Body weight change of MRL/lpr mice treated with ND or HID (n=8). (**B**) Serum iron levels of 23-weeks old MRL/lpr mice treated with ND or HID (n=8). (**C-D**) Representative flow cytometry (**C**) and quantification (**D**) of $CD3^{+}CD4^{+}CD8^{-}$T cells,

$CD3^+CD8^+CD4^-$T cells, and $CD3^+CD4^-CD8^-$ double negative (DN) T cells in dLNs and spleen of MRL/lpr mice after 20-weeks of ND or HID treatment (n=8). (**E**) Representative flow cytometry and quantification of $CD11b^+F4/80^+$ macrophages in spleen of MRL/lpr mice after 20-weeks of ND or HID treatment (n=8). (**F**) Representative flow cytometry and quantification of DCs in spleen of MRL/lpr mice after 20-weeks of ND or HID treatment (n=8). cDCs were defined as $B220^-CD11c^+$ cells, pDCs were defined as $B220^+CD11c^+$ cells, total DCs were defined as $CD11c^+$ cells. (**G**) Representative flow cytometry and quantification of $B220^+CD4^-$ B cells in dLNs and spleen of ND- or HID-fed MRL/lpr mice (n=8). (**H**) Representative histology and quantification of GCs in spleen after 20-weeks of ND or HID treatment (n=6). Green PNA; blue DAPI. Scale bar, 100 μm. (**I**) Representative flow cytometry and quantification of $CD4^-B220^-CD138^+$ plasma cells in dLNs and spleen of ND- or HID-fed MRL/lpr mice (n=8). (**J**) immunofluorescent staining of $CD4^+$ T cells and quantification of $CD4^+$ T cell infiltration in kidney after 20-weeks of ND or HID treatment (n=8). Green CD4; blue DAPI. Scale bar, 100 μm. Data are shown as mean ± SEM. Data are representative of 2 independent experiments. ****P < 0.0001, ***P < 0.001, **P<0.01, *P<0.05 (unpaired two-tailed Student's *t*-test for **A**, **B**, **D-J**).

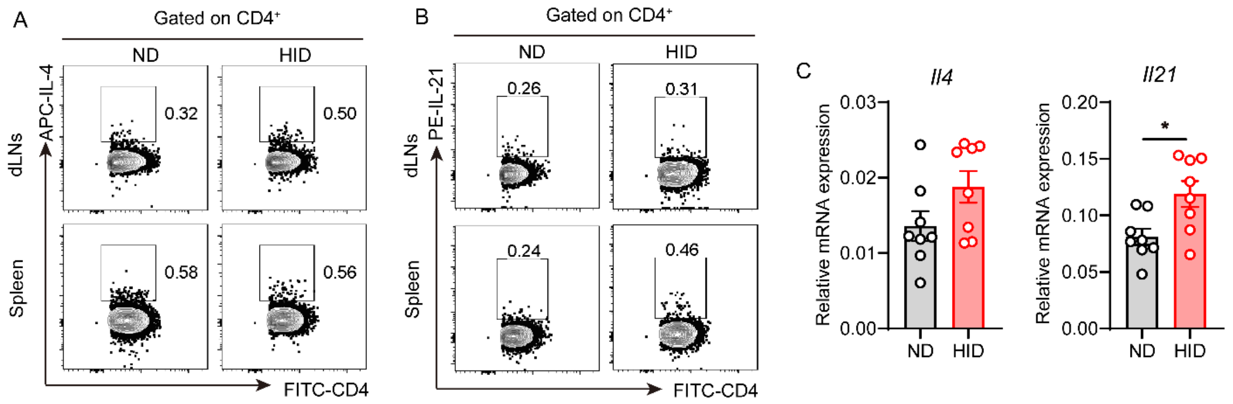

Supplemental Figure 4. The expression of IL-4 and IL-21 in $CD4^+$ T cells of MRL/lpr mice fed with ND or HID. **A** and **B** Representative flow cytometry of IL-4 (**A**) and IL-21 (**B**) in $CD4^+$ T cells of MRL/lpr mice fed with HID. **C** qPCR of *Il4* and *Il21* in $CD4^+$ T cells of MRL/lpr mice fed with ND (n=8) or HID (n=8). Data are shown as mean ± SEM. Data are representative of 2 independent experiments. *P < 0.05 (unpaired two-tailed Student's *t*-test for **C**).

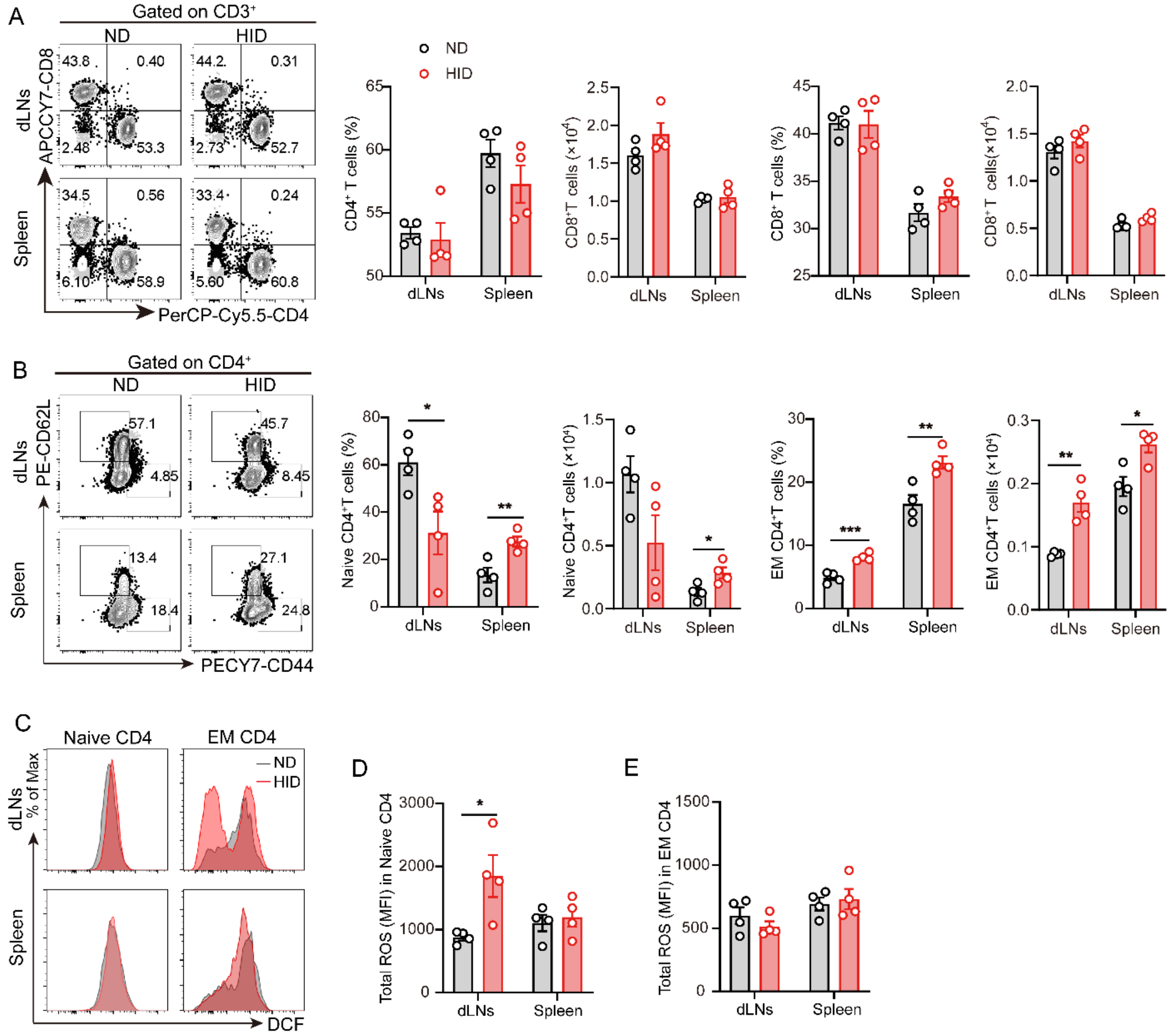

Supplemental Figure 5. Phenotypic observation of HID-treated mice. 3-weeks old female B6 mice were treated with 50 mg/kg normal iron diet (ND, n=4) or 500 mg/kg high iron diet (HID, n=4). After 7-weeks of HID treatment, mice were sacrificed for analysis. (**A**) Representative flow cytometry and quantification of total CD4$^+$ T cells and CD8$^+$ T cells in dLNs and spleen of B6 mice fed with 7 weeks of ND or HID. (**B**) Representative flow cytometry and quantification of CD4$^+$CD62L$^+$CD44$^-$ naive CD4$^+$ T cells and CD4$^+$ CD44$^+$ CD62L$^-$ EM CD4$^+$ T cells in dLNs and spleen of B6 mice fed with ND or HID. (**C-E**) Representative flow cytometry (**C**) and quantification of total ROS in naive CD4$^+$ T cells (**D**) and EM CD4$^+$ T cells (**E**). Data are shown as mean ± SEM. Data are representative of 2 independent experiments. ***P < 0.001, **P<0.01, *P<0.05 (unpaired two-tailed Student's *t*-test for **A**, **B**, **D**, **E**).

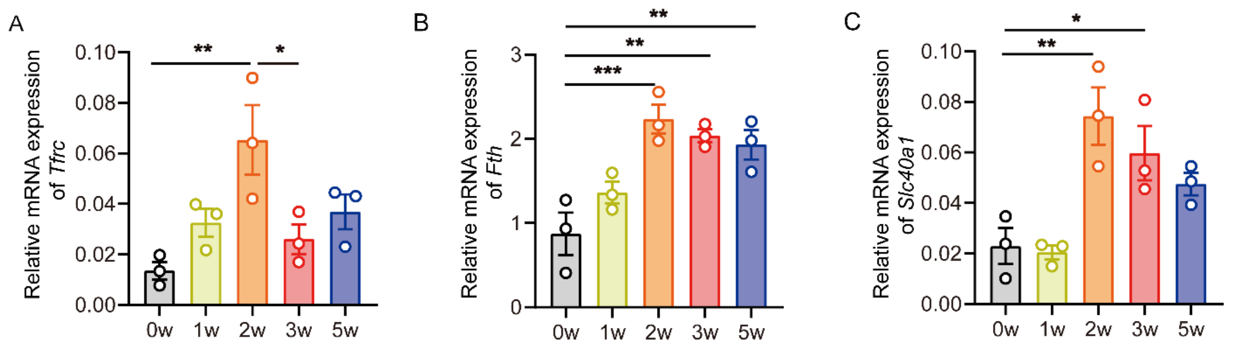

Supplemental Figure 6. The expression of iron-related genes in splenic CD4$^+$ T cells during the process of HID treatment. Female B6 mice were fed with HID for different times and sacrificed for analysis. (**A-C**) qPCR of *Tfrc* (**A**), *Fth* (**B**), and *Slc40a1* (**C**) in splenic CD4$^+$ T cells after 0, 1, 2, 3, 5-weeks of HID treatment (n=3 for each group). Data are shown as mean ± SEM. Data are representative of 2 independent experiments. ***P < 0.001, **P<0.01, *P<0.05 (One way-ANOVA and Tukey's multiple comparisons test for **A**-**C**).

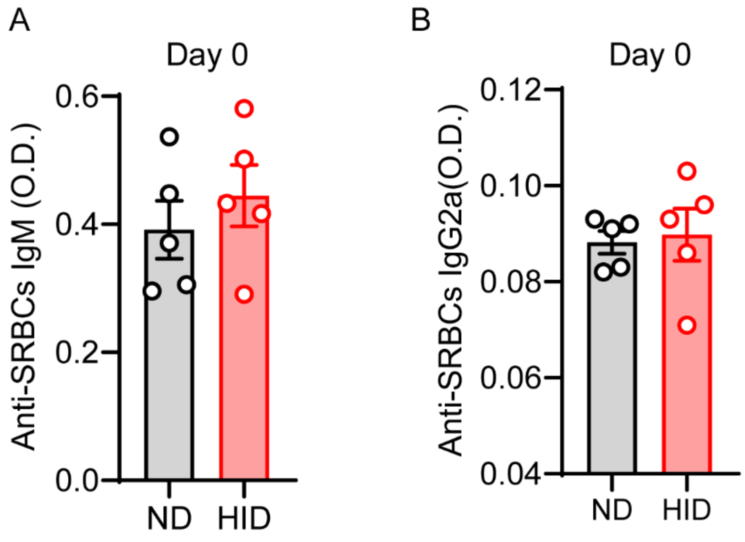

Supplemental Figure 7. Serum levels of anti-SRBCs IgM and IgG2a at day 0 of SRBCs immunization. Serum levels of anti-SRBCs IgM (**A**) and anti-SRBCs IgG2a (**B**) in ND- and HID-treated mice at day 0 of SRBCs immunization (n=5 for each group). Data are shown as mean ± SEM. Data are representative of 2 independent experiments. For **A** and **B**, unpaired two-tailed Student's *t*-test was used.

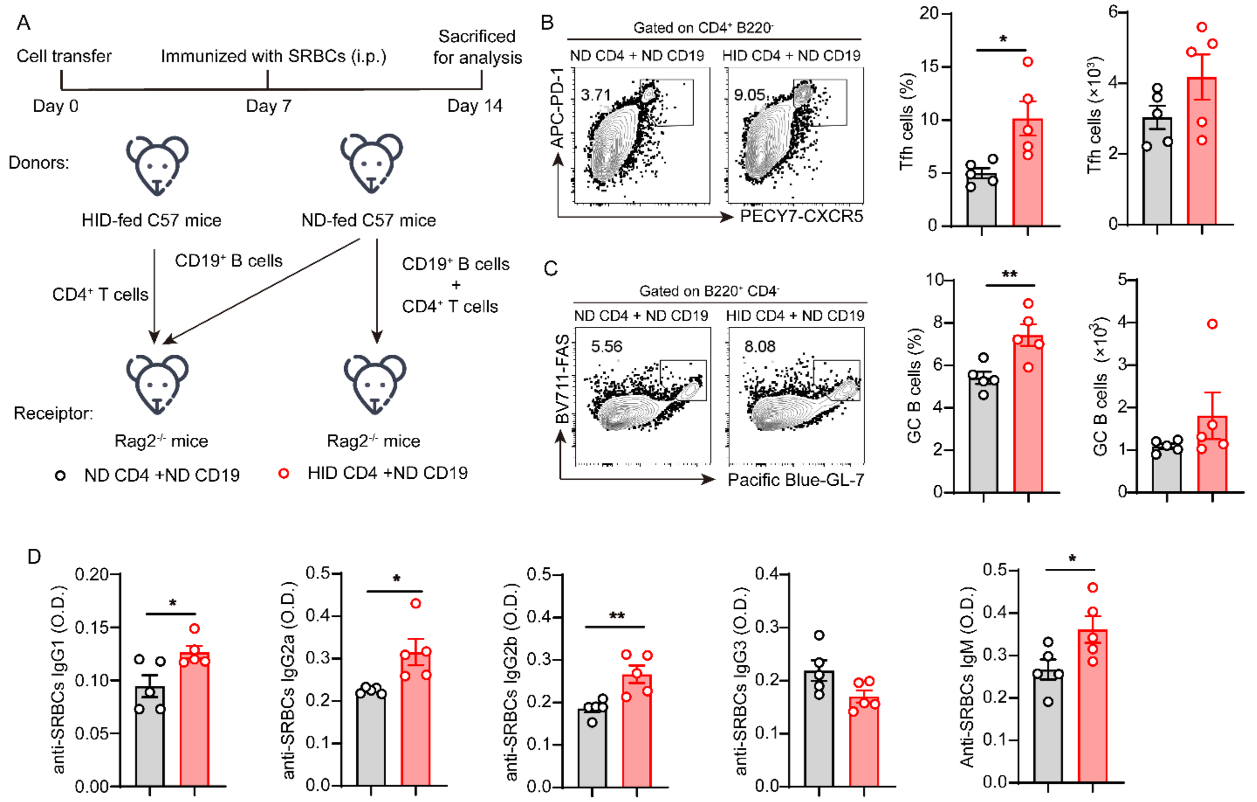

Supplemental Figure 8. The increase of anti-SRBCs antibodies after HID treatment is T cell-dependent. T/B cell suspensions were transferred into the 8-weeks old Rag2$^{-/-}$ mice by tail vein injection. After 7 days of T/B cell transfer, Rag2$^{-/-}$ were immunized with SRBCs by intraperitoneal injection. Mice were sacrificed for analysis after 7 days of SRBCs immunization. (**A**) Schematic diagram of the cell transfer and SRBCs immunization in Rag2$^{-/-}$ mice. (**B** and **C**) Representative flow cytometry and quantification of CD4$^+$CXCR5$^+$PD-1$^+$ Tfh cells (**B**) and B220$^+$FAS$^+$GL-7$^+$ GC B cells (**C**) in the spleen of Rag2$^{-/-}$ mice receiving HID CD4$^+$ T cells and ND CD19$^+$ B cells or ND CD4$^+$ T cells and ND CD19$^+$ B cells. (**D**) The serum levels of anti-SRBCs IgG1, IgG2a, IgG2b, IgG3, and IgM in Rag2$^{-/-}$ mice receiving HID CD4$^+$ T cells and ND CD19$^+$ B cells (n=5) or ND CD4$^+$ T cells and ND CD19$^+$ B cells (n=5). Data are shown as mean ± SEM. Data are representative of 2 independent experiments. $^{**}P < 0.01$, $^{*}P < 0.05$ (unpaired two-tailed Student's *t*-test for **B**-**D**).

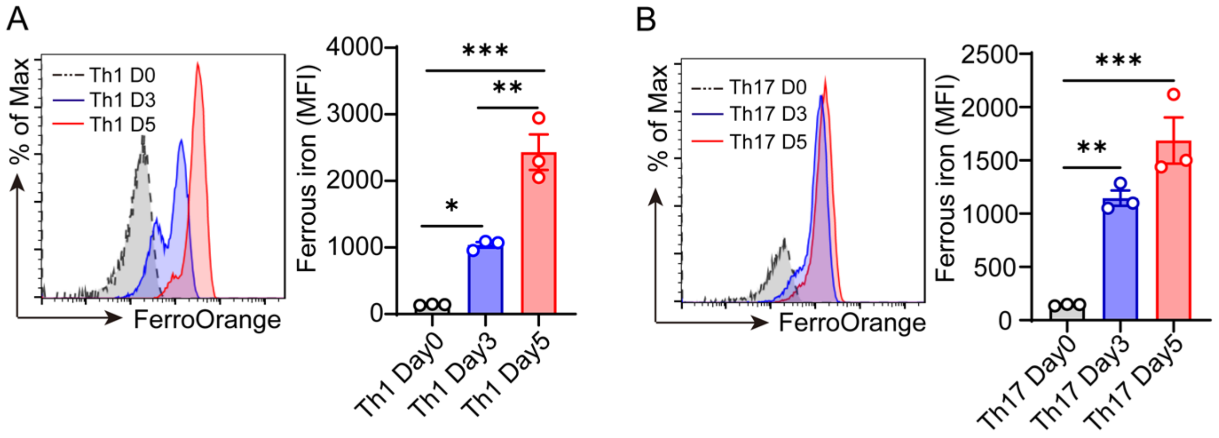

Supplemental Figure 9. The levels of intracellular iron during the differentiation processes of Th1 and Th17 cells. Human naive T cells were isolated from the peripheral blood of healthy donors, and then cultured under Th1 and Th17 cell-polarized conditions for 0, 3, 5 days respectively. (**A**) Representative flow cytometry and quantification of ferrous iron during the differentiation process of Th1 cells (n=3). (**B**) Representative flow cytometry and quantification of ferrous iron during the differentiation process of Th17 cells (n=3). Data are shown as mean ± SEM. Data are representative of 2 independent experiments. ***P<0.001, **P<0.01, *P<0.05 (one-way ANOVA with Tukey's multiple comparisons test for **A** and **B**)

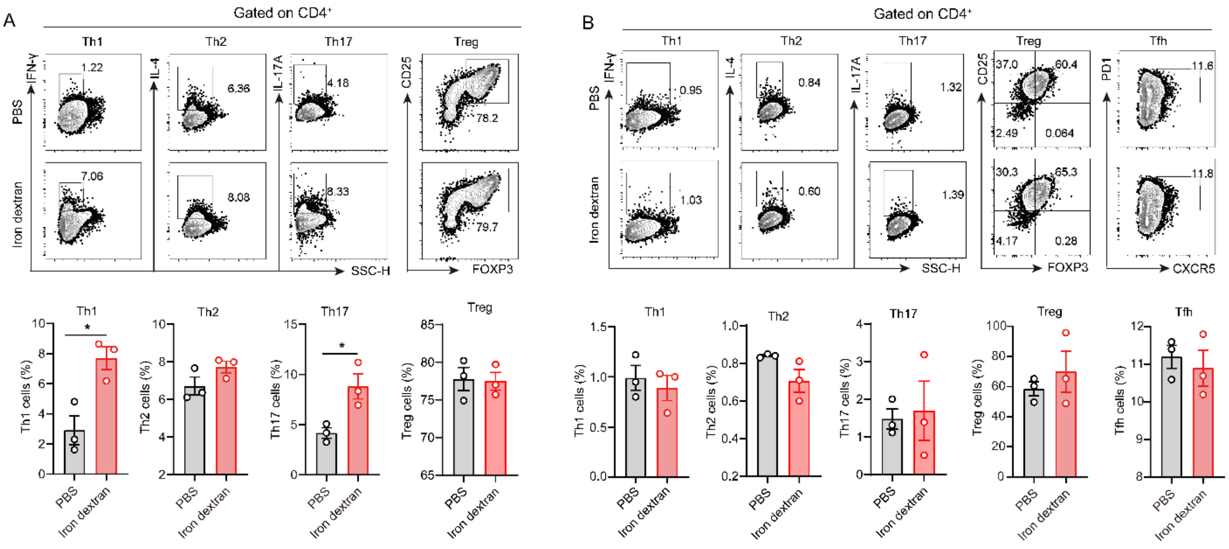

Supplemental Figure 10. The role of iron to Th cell polarization. (**A**) Naive CD4$^+$ T cells were isolated from splenic cells of 8-12 weeks old B6 mice (n=3), and then treated with PBS or iron dextran 20 μM, and then cultured under Th1, Th2, Th17, and Treg cell-polarized conditions for 3 days. After 3 days of polarization, cells were collected to determine the percentages of Th1, Th2, Th17, and Treg cells respectively. (**B**) CD4$^+$ T cells were isolated from splenic cells of 8-12 weeks old female B6 mice (n=3), and treated with PBS or iron dextran 20 μM in the presence of anti-CD3 5 μg/mL and anti-CD28 2 μg/mL for 2 days. After 2 days of stimulation, cells were collected to determine the percentages of Th cell subsets. Th1 cells were defined as CD4$^+$IFN-γ$^+$ cells, Th2 cells were defined as CD4$^+$IL-4$^+$ cells, Th17 cells were determined as CD4$^+$IL-17A$^+$ cells, Treg cells were determined as CD4$^+$CD25$^+$FOXP3$^+$ cells, Tfh cells were defined as CD4$^+$CXCR5$^+$PD-

1+ cells. Data are shown as the mean ± SEM. Data are representative of 2 independent experiments with 3 individuals. *P < 0.05 (unpaired two-tailed Student's *t*-test for **A**-**B**).

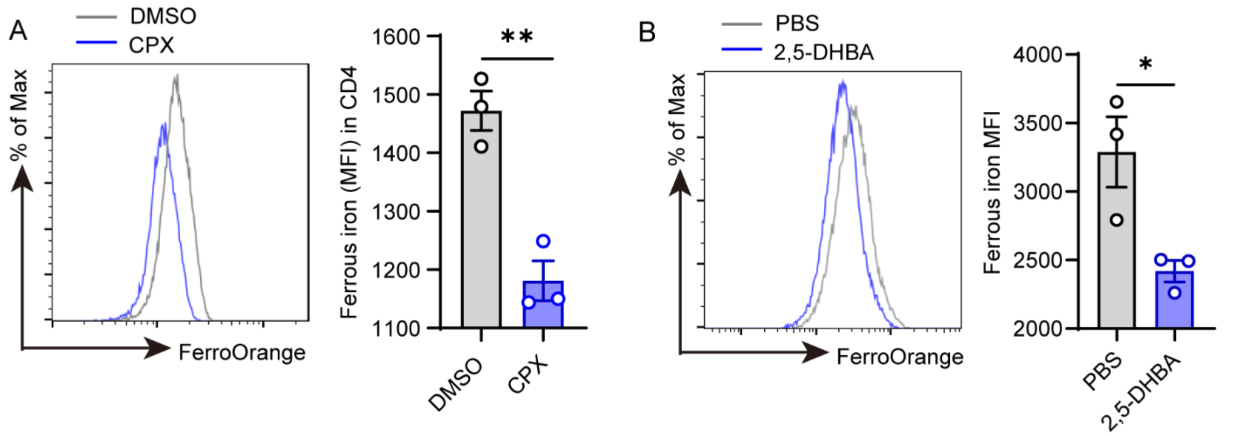

Supplemental Figure 11. The efficiency of iron chelators. (**A**) CD4+ T cells were isolated from peripheral blood of healthy donors and treated with CPX 20 µm or DMSO for 4 hours, and then cells were collected for analysis. Representative flow cytometry and quantification of ferrous iron MFI in CD4+ T cells treated with DMSO or CPX 20 µm for 4 hours. (**B**) CD4+ T cells were treated with 2,5-DHBA 20 µm or PBS in the presence of anti-CD3/CD28 for 2 days. Representative flow cytometry and quantification of ferrous iron MFI in CD4+ T cells treated with PBS or 2,5-DHBA for 2 days. Data are shown as mean ± SEM. Data are representative of 2 independent experiments with 3 donors. **P<0.01, *P<0.05 (unpaired two-tailed Student's *t*-test for **A**-**B**).

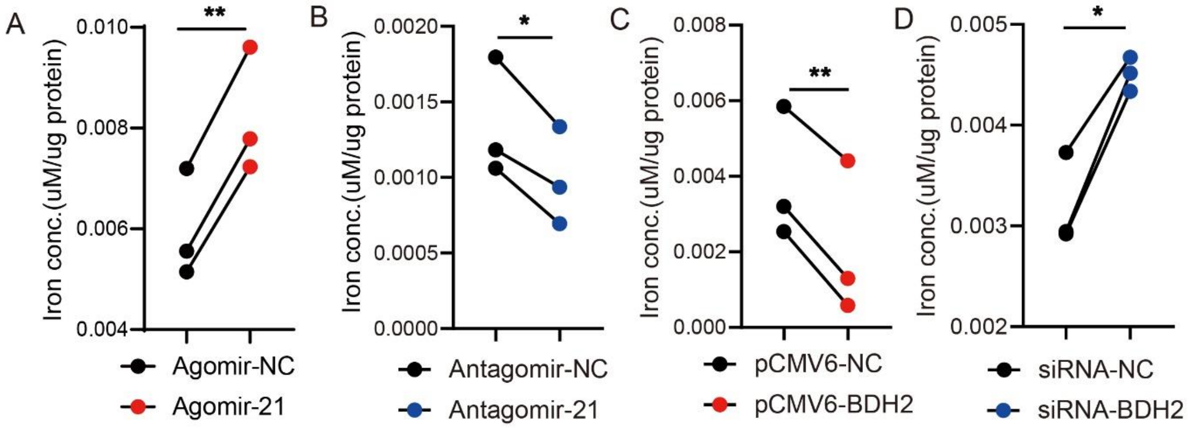

Supplemental Figure 12. miR-21/BDH2 modulates intracellular iron in Tfh cells. miR-21/BDH2 modulates intracellular iron in Tfh cells. (**A**-**D**) Healthy naive CD4+ T cells were transfected with Agomir-21(**A**), Antagomir-21(**B**), pCMV6-BDH2(**C**), and siRNA-BDH2(**D**) respectively, and cultured under Tfh-polarized conditions in vitro for 3 days. After 3 days of Tfh polarization, cells were collected for intracellular iron analysis. Data are shown as mean ± SEM. Data are representative of 2 independent experiments with 3 donors. ****P<0.0001, ***P<0.001, **P<0.01, *P<0.05 (paired two-tailed Student's *t*-test for **A**-**D**).

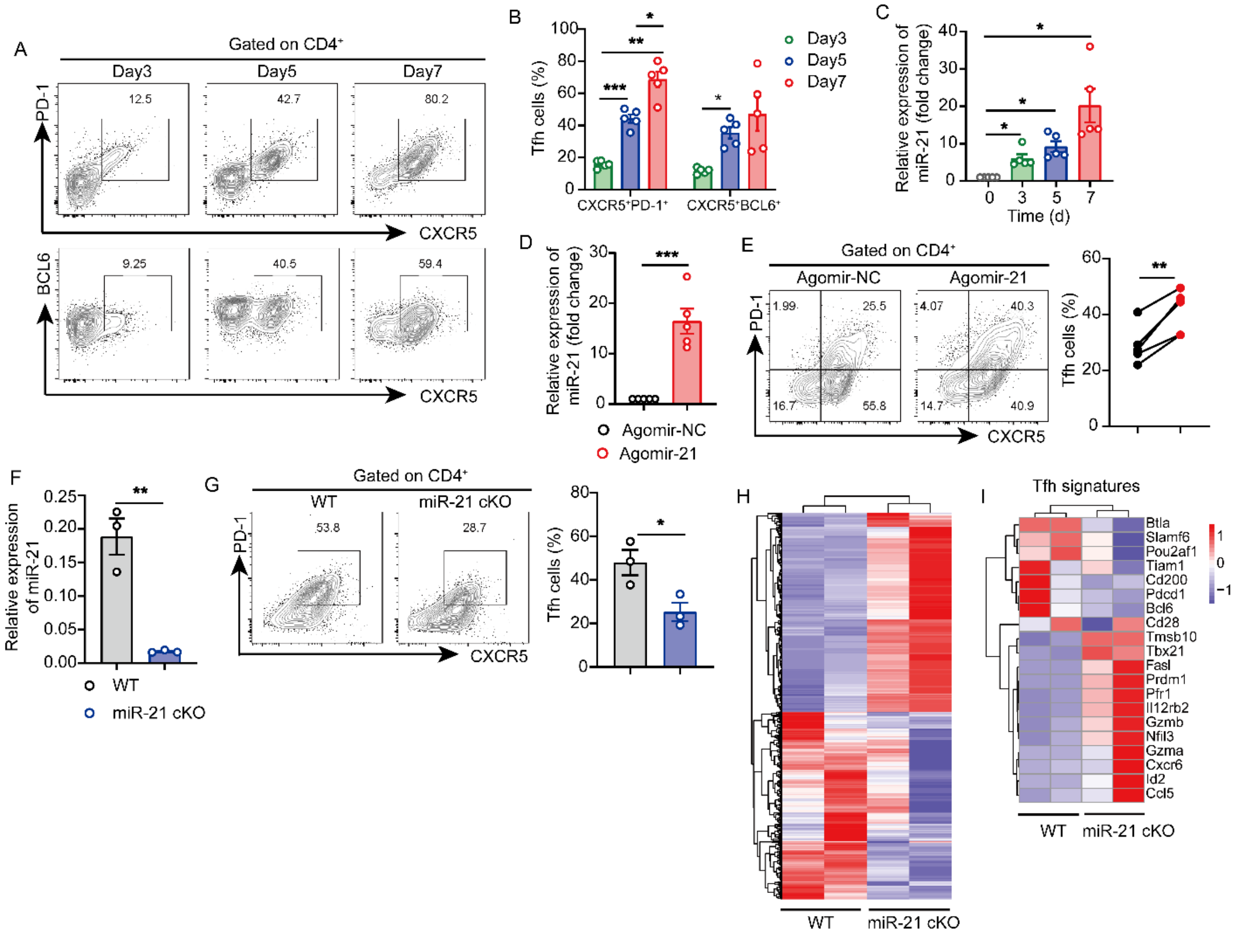

Supplemental Figure 13. miR-21 contributes to murine Tfh cell differentiation in vitro. (**A**-**B**) Representative flow cytometry (**A**) and quantification (**B**) of induced Tfh cells after 3, 5, 7 days of Tfh polarization (n=5). (**C**) qPCR of miR-21 in naive CD4$^+$ T cells and Tfh cells induced in **A**. (**D**-**E**) naive CD4$^+$ T cells isolated from B6 mice (n=5) were transfected with Agomir-NC or Agomir-21 and cultured under Tfh-polarized conditions for 5 days. After 5 days of differentiation, qPCR of miR-21 (**D**), flow cytometry and quantification of CD4$^+$CXCR5$^+$PD-1$^+$ Tfh cells were analyzed (**E**). (**F**-**G**) Naive CD4$^+$ T cells isolated from WT (n=3) or miR-21 cKO mice (n=3) were cultured under Tfh-polarized conditions for 5 days, and then cells were collected to analyze the expression of miR-21 (**F**) and flow cytometry and quantification of CD4$^+$CXCR5$^+$PD-1$^+$ Tfh cells (**G**). (**H**) Microarray heat-map of genes differentially expressed between WT and miR-21 cKO Tfh cells induced in vitro. (**I**) Microarray heat-map of Tfh signatures differentially expressed between WT and miR-21 cKO Tfh cells induced in vitro. Data are shown as the mean ± SEM. Data are representative of at least 2 independent experiments. ****P<0.0001, ***P<0.001, **P<0.01, *P<0.05 (one-way ANOVA with Tukey's multiple comparisons test for **B** and **C**; two-tailed Student's *t*-test for **D**-**G**).

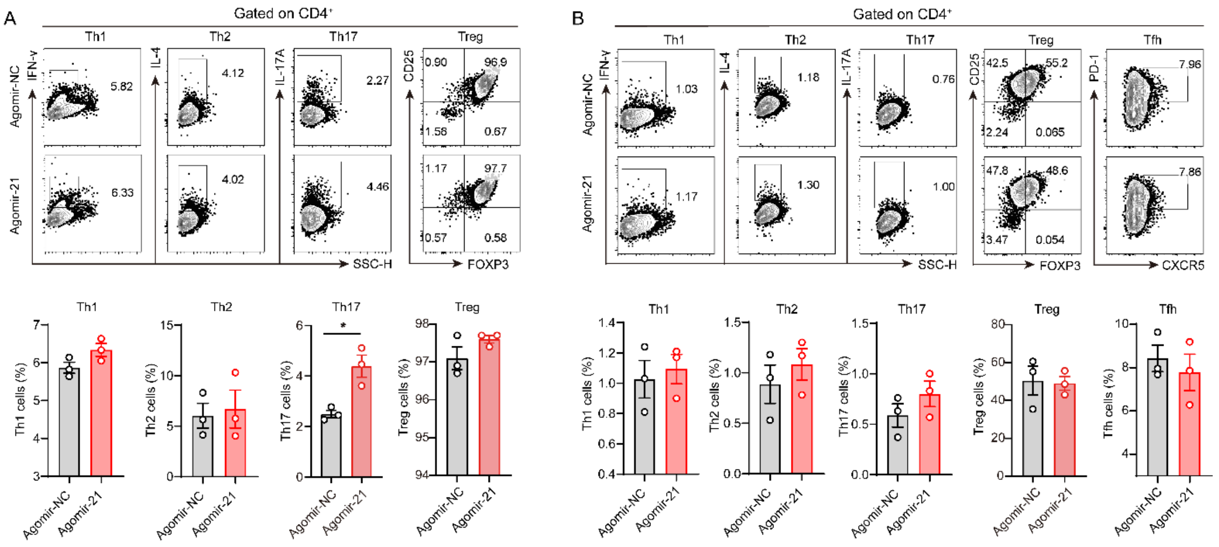

Supplemental Figure 14. The role of miR-21 to Th cell polarization. (**A**) naive $CD4^+$ T cells were isolated from splenic cells of 8-12 weeks old B6 mice (n=3), and then treated with Agomir-NC or Agomir-21 200 nM and cultured under Th1, Th2, Th17, and Treg cell-polarized conditions for 3 days respectively. After 3 days of polarization, cells were collected to determine the percentages of Th1, Th2, Th17, and Treg cells respectively. (**B**) $CD4^+$ T cells were isolated from splenic cells of 8-12 weeks old female B6 mice (n=3), and treated with Agomir-NC or Agomir-21 200 nM in the presence of anti-CD3 5 μg/mL and anti-CD28 2 μg/mL for 2 days. After 2 days of stimulation, cells were collected to determine the percentage of Th cell subsets. Th1 cells were defined as $CD4^+IFN\text{-}\gamma^+$ cells, Th2 cells were defined as $CD4^+IL\text{-}4^+$ cells, Th17 cells were determined as $CD4^+IL\text{-}17A^+$ cells, Treg cells were determined as $CD4^+CD25^+FOXP3^+$ cells, Tfh cells were defined as $CD4^+CXCR5^+PD\text{-}1^+$ cells. Data are shown as the mean ± SEM. Data are representative of 2 independent experiments. *P < 0.05 (Unpaired two-tailed Student's *t*-test was used for **A**-**B**).

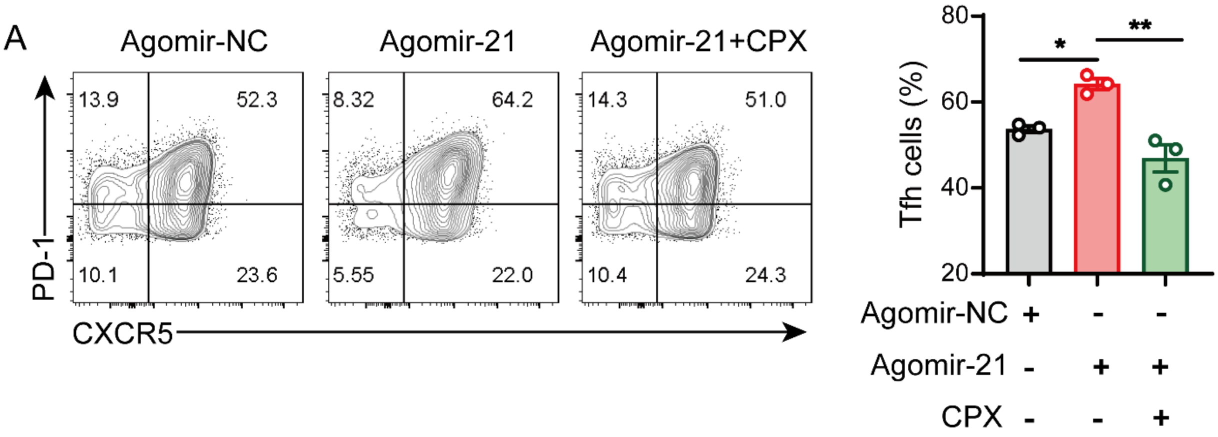

Supplemental Figure 15. Iron chelator CPX recovers the effect of miR-21 in Tfh cell differentiation. (**A**) Representative flow cytometry and quantification of $CD4^+CXCR5^+PD\text{-}1^+$ Tfh percentage in cells transfected with either Agomir-NC, Agomir-21, or Agomir-21 plus CPX (n=3). Data are shown as the mean ± SEM. Data are representative of 2 independent experiments. **P<0.01, *P<0.05 (one-way ANOVA with Tukey's multiple comparisons test for **A**).

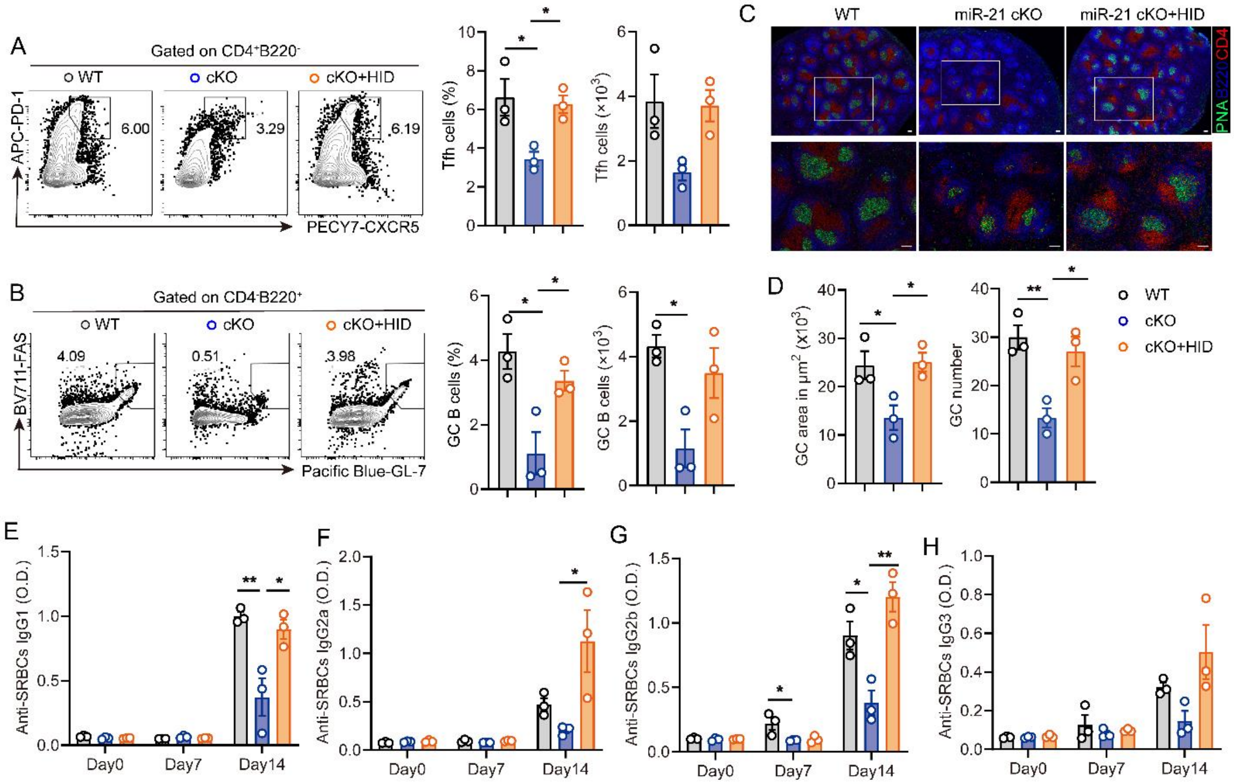

Supplemental Figure 16. HID recovers the defect of humoral immunity in miR-21 cKO mice. 3-weeks old WT mice were fed with ND (n=3) and age-matched miR-21 cKO mice were fed with ND (n=3) or HID (n=3) for 5 weeks, and then immunized with SRBCs. After 2 weeks of SRBCs stimulation, mice were sacrificed for analysis. (**A**) Representative flow cytometry and quantification of $CD4^+CXCR5^+PD\text{-}1^+$ Tfh cells in the spleen of WT mice, miR-21 cKO mice, and miR-21 cKO mice fed with HID respectively. (**B**) Representative flow cytometry and quantification of $B220^+GL\text{-}7^+FAS^+$ GC B cells in the spleen of WT mice, miR-21 cKO mice, and miR-21 cKO mice fed with HID respectively. (**C** and **D**) Representative histology and quantification of GCs in spleen after 2-weeks of SRBCs immunization. Red CD4; blue, B220; green, PNA. Scar bar, 100 µM. (**E**-**H**) The titers of anti-SRBCs IgG1 (**E**), IgG2a (**F**), IgG2b (**G**), IgG3 (**H**) in WT mice, miR-21 cKO mice, and miR-21 cKO mice fed with HID respectively. Data are shown as the mean ± SEM. Data are representative of 2 independent experiments. $^{**}P < 0.01$, $^{*}P < 0.05$ (one-way ANOVA with Tukey's multiple comparisons test for **A**-**B**, **D**-**H**).

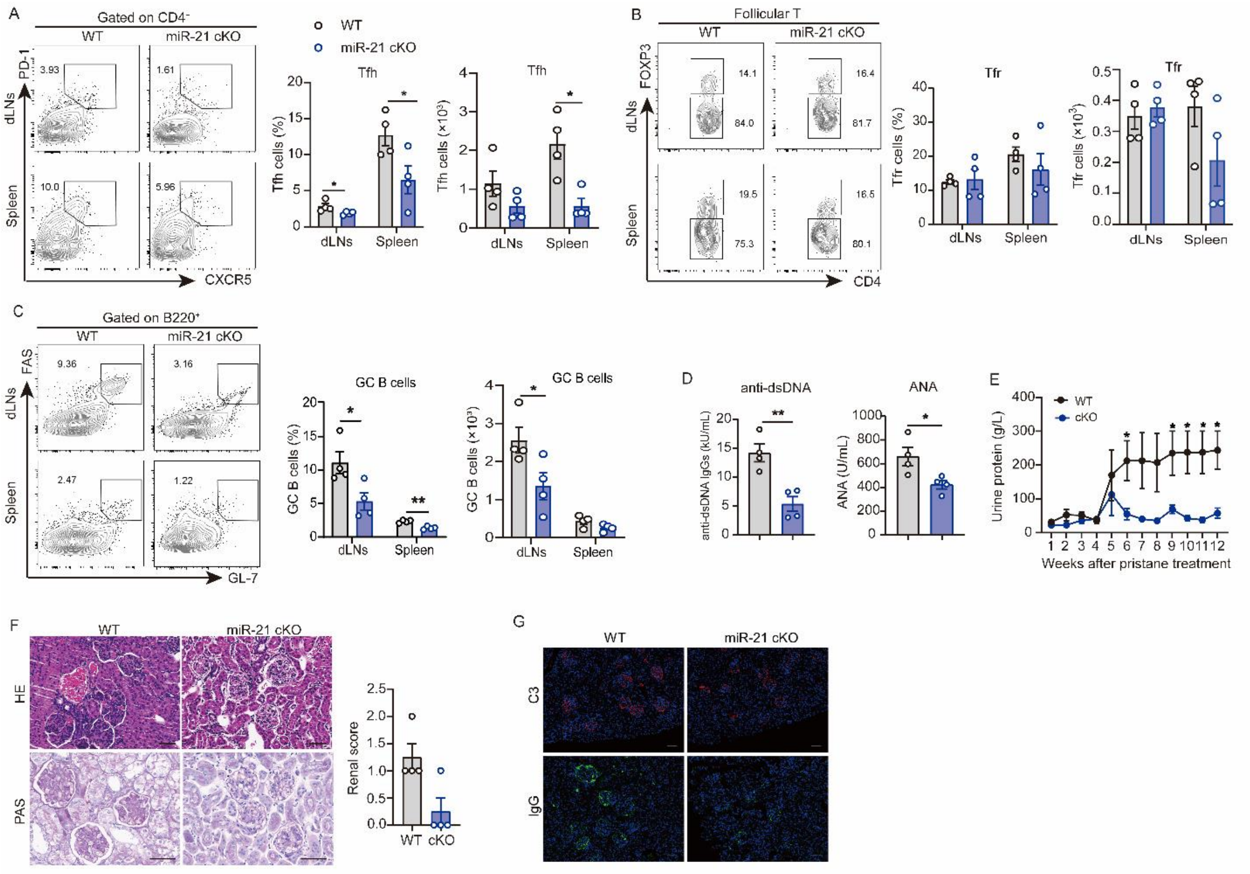

Supplemental Figure 17. miR-21 contributes pristane-induced lupus in mice. 12-weeks old WT (n=4) or miR-21 cKO mice (n=4) were intraperitoneally injected with 500 μL of pristane. After 12 weeks of stimulation, mice were sacrificed for analysis. (**A**) Representative flow cytometry and quantification of CD4$^+$CXCR5$^+$PD-1$^+$Tfh cells gated on CD4$^+$ T cells. (**B**) Representative flow cytometry and quantification of CD4$^+$CXCR5$^+$PD-1$^+$FOXP3$^+$ Tfr cells gated on CD4$^+$CXCR5$^+$PD-1$^+$ Tfh cells. (**C**) Representative flow cytometry and quantification of GC B cells gated on B220$^+$ B cells. (**D**) ELISA of anti-dsDNA IgG and ANA total Ig after 12 weeks of pristane treatment. (**E**) Urine protein of WT or miR-21 cKO mice stimulated with pristane. (**F**) Representative morphology (by H&E and PAS staining) of the kidney after 12 weeks of pristane treatment. Scale bar, 50 μm. (**G**) Representative histology of kidney after 12 weeks of pristane treatment. Red, C3; green, IgG. Scale bar, 50 μm. Cells were isolated from dLNs and spleen of WT or miR-21 cKO mice. Data are shown as the mean ± SEM. Data are representative of 2 independent experiments. **P<0.01, *P<0.05 (unpaired two-tailed Student's *t*-test for **A**-**D**; unpaired two-tailed Mann-Whitney *U* tests for **E** and **F**).

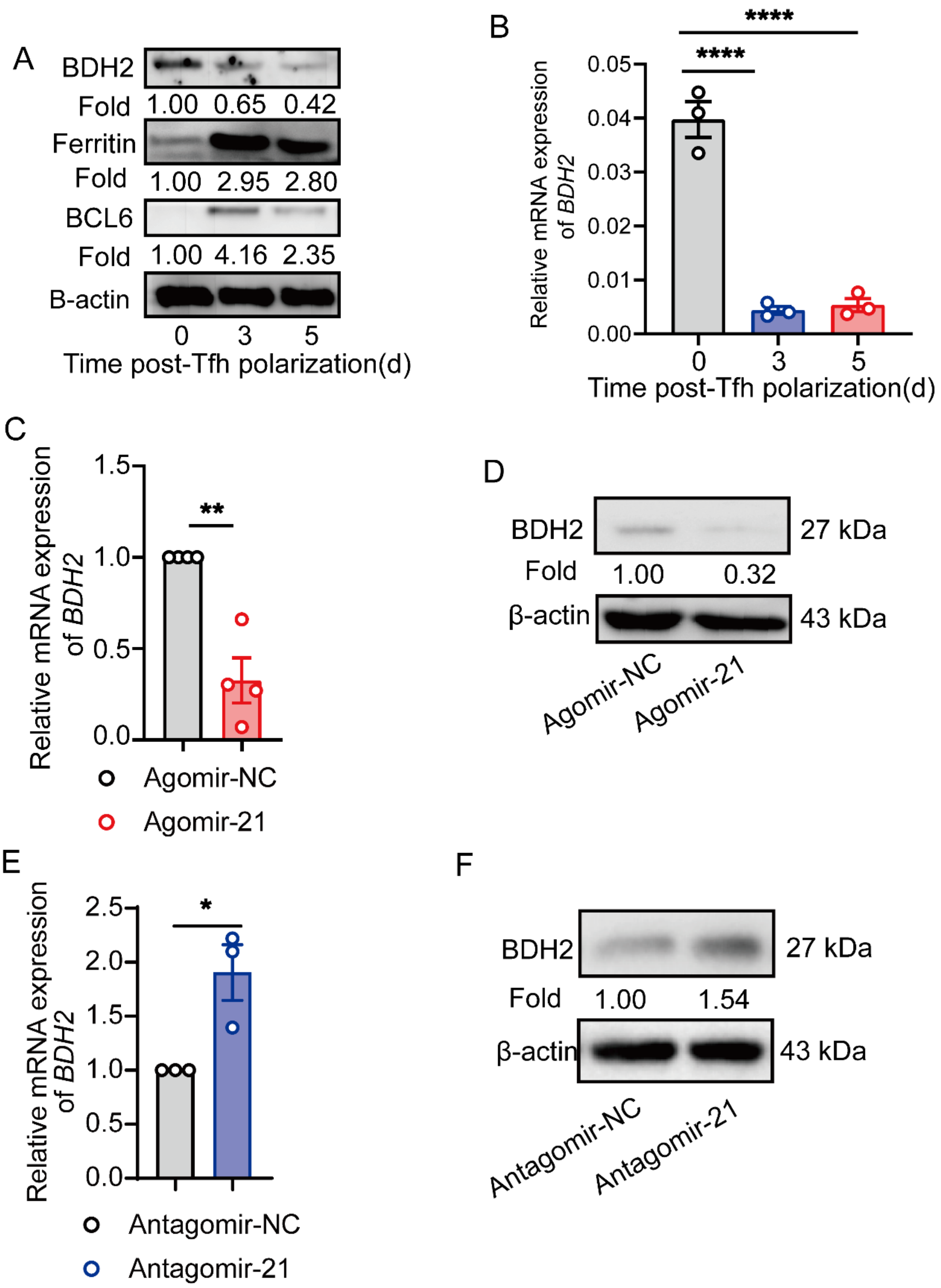

Supplemental Figure 18. *BDH2* is the target gene of miR-21 in regulating Tfh cell differentiation. (**A-B**) Healthy naive $CD4^+$ T cells were cultured under Tfh-polarization for 0, 3, 5 days respectively. Western blot was used to determine the protein expression of BDH2, BCL6, and Ferritin (**A**), and qPCR was used to determine the mRNA expression of *BDH2* in cells cultured under Tfh-polarized conditions for 0, 3, 5 days (**B**). (**C** and **D**) Healthy naive $CD4^+$ T cells were transfected with Agomir-NC or Agomir-21 and cultured under Tfh-polarization for 3 days. qPCR (**C**) and Western blot (**D**) were used to determine the expression of BDH2 in cells transfected with

Agomir-NC or Agomir-21. (**E** and **F**) Healthy naive CD4$^+$ T cells were transfected with Antagomir-NC or Antagomir-21 and cultured under Tfh-polarization for 3 days. qPCR (**E**) and Western blot (**F**) were used to determine the expression of BDH2 in cells transfected with Antagomir-NC or Antagomir-21. Data are shown as the mean ± SEM. Data are representative of 3 independent experiments with 3 or 4 donors. ****P<0.0001, **P<0.01, *P<0.05 (one-way ANOVA with Tukey's multiple comparisons test for **B**; Unpaired two-tailed Student's *t*-test for **C**, **E**).

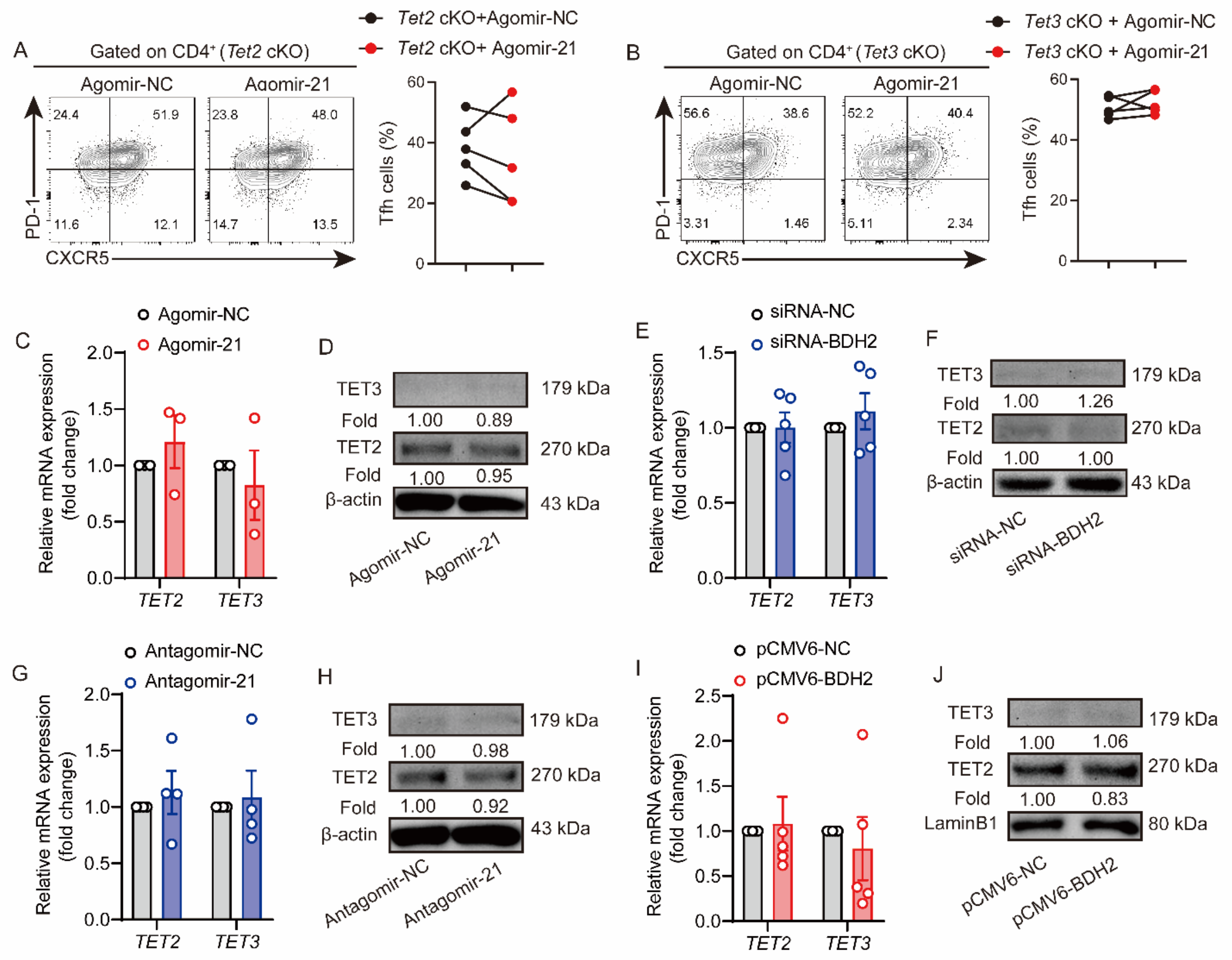

Supplemental Figure 19. miR-21/BDH2 axis regulates TET enzyme activity independent of TET enzyme expression. (**A-B**) naive CD4$^+$ T cells were isolated from the spleen of *Tet2* cKO (**A**) or *Tet3* cKO mice (**B**) and transfected with Agomir-NC or Agomir-21, and then cultured under Tfh-polarized conditions for 3 days. After 3 days of Tfh polarization, the percentage of induced Tfh cells was detected by flow cytometry (n=5). (**C-J**) Human naive CD4$^+$ T cells were isolated from peripheral blood of healthy donors, and cultured under Tfh cell polarized conditions for 3 days. qPCR (**C**) and representative Western blot (**D**) of TET2 and TET3 in induced human Tfh cells transfected with Agomir-NC and Agomir-21. qPCR (**E**) and representative Western blot (**F**) of TET2 and TET3 in induced human Tfh cells transfected with siRNA-NC and siRNA-BDH2. qPCR (**G**) and representative Western blot (**H**) of TET2 and TET3 in induced human Tfh cells transfected with Antagomir-NC and Antagomir-21. qPCR (**I**) and representative Western blot (**J**) of TET2 and TET3 in induced human Tfh cells transfected with pCMV6-NC and pCMV6-BDH2. Data are

shown as the mean ± SEM. For **C**-**J**, data are representative of 2 independent experiments with at least 3 donors per group (Two-tailed Student's *t*-test for **A**, **C**, **E**, **G**, **I**, **J**).

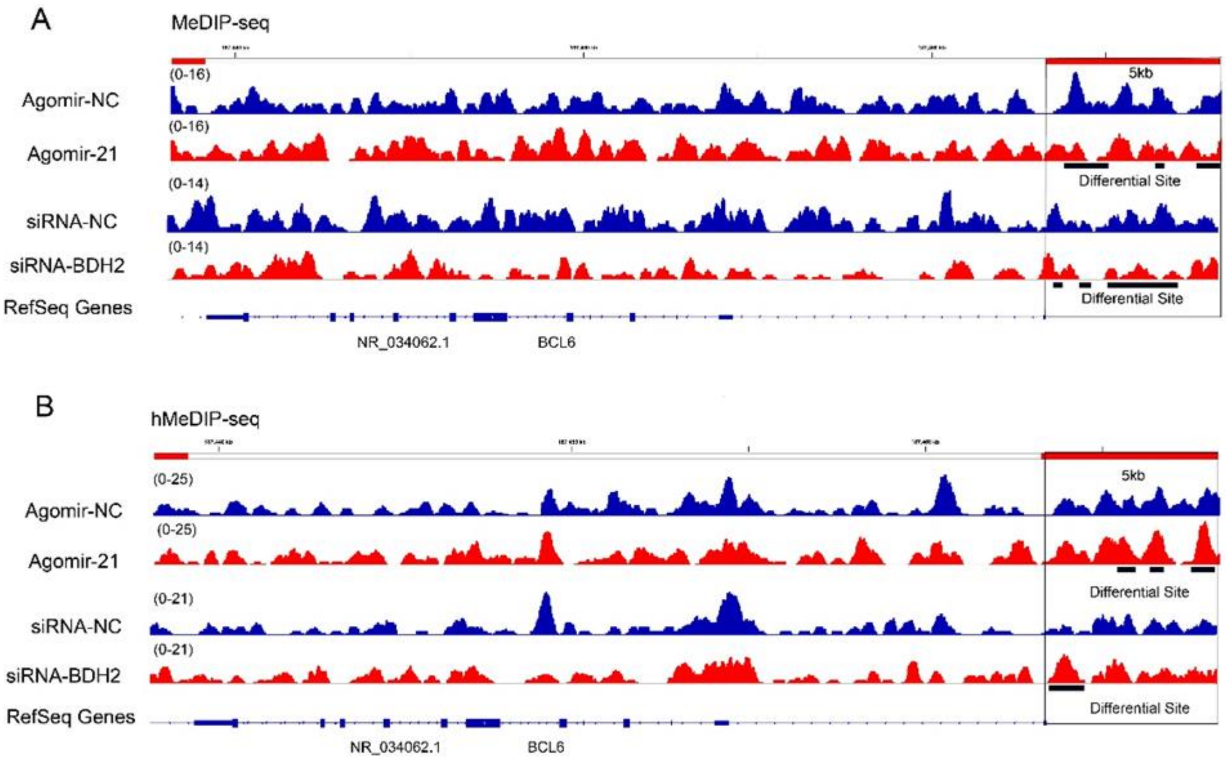

Supplemental Figure 20. Representative IGV profiles of 5-mC MeDIP-seq and 5-hmC hMeDIP-seq of *BCL6* gene. (**A**) Representative IGV profiles of 5-mC MeDIP-seq of *BCL6* gene in induced Tfh cells transfected with Agomir-21 or siRNA-BDH2. (**B**) Representative IGV profiles of 5-hmC hMeDIP-seq of *BCL6* gene in induced Tfh cells transfected with Agomir-21 or siRNA-BDH2. The inset figures show the differential sites in the upstream 5000 bp of transcription starting site (TSS).

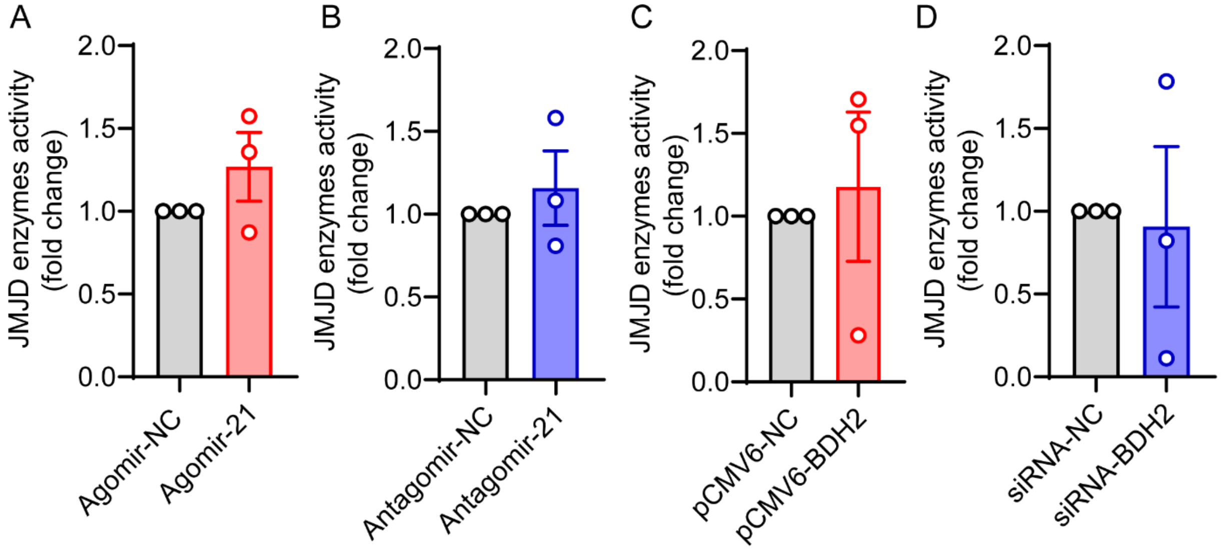

Supplemental Figure 21. The activity of JMJD3 enzyme. Human naive CD4$^+$ T cells were transfected with Agomir-21, Antagomir-21, pCMV6-BDH2, and siRNA-BDH2 respectively, and cultured under Tfh-polarized conditions for 3 days. After 3 days of stimulation, cells were collected to analyze the JMJD3 activity. (**A**) JMJD activity of induced Tfh cells transfected with Agomir-NC or Agomir-21. (**B**) JMJD3 activity of induced Tfh cells transfected with Antagomir-NC or Antagomir-21. (**C**) JMJD3 activity of induced Tfh cells transfected with pCMV6-NC or pCMV6-BDH2. (**D**) JMJD3 activity of induced Tfh cells transfected with siRNA-NC or siRNA-BDH2. Two-tailed Student's *t*-test for **A**-**D**. Data are shown as the mean ± SEM. Data are representative of 2 independent experiments with 3 donors.

Supplemental Tables

Supplemental Table 1. Patient information

Number	Sex	Age	SLEDAI score
01	Female	13	4
02	Female	23	8
03	Female	39	2
04	Female	51	4
05	Female	19	12
06	Female	56	0
07	Female	48	2
08	Female	53	0
09	Female	51	12
10	Female	29	4
11	Female	27	4
12	Female	43	7
13	Female	37	4
14	Female	70	6
15	Female	31	6
16	Female	14	4
17	Female	47	0
18	Female	28	4
19	Female	42	9
20	Female	52	6
21	Female	56	13
22	Female	51	0
23	Female	48	0
24	Female	43	0
25	Female	24	22
26	Female	19	0
27	Female	24	4
28	Female	19	0
29	Female	24	4
30	Female	30	2
31	Female	20	4
32	Female	52	4

33	Female	27	6
34	Female	35	8
35	Female	61	2
36	Female	37	8
37	Female	31	0
38	Female	26	0
39	Female	17	2
40	Female	54	0
41	Female	45	2
42	Female	39	2
43	Female	23	5
44	Female	47	2
45	Female	46	0
46	Female	28	12
47	Female	22	2
48	Female	36	6
49	Female	50	5
50	Female	43	0
51	Female	48	7
52	Female	47	0
53	Female	36	10
54	Female	27	1
55	Female	23	10
56	Male	27	4
57	Female	31	8
58	Female	36	2
59	Female	23	10
60	Female	37	6
61	Female	43	12
62	Male	50	0
63	Female	55	4
64	Female	41	4
65	Female	18	2
66	Male	30	2
67	Female	31	0
68	Male	48	9
69	Female	48	6
70	Male	45	4
71	Female	45	2
72	Female	45	2
73	Female	50	6
74	Female	38	4
75	Female	45	4
76	Female	34	4
77	Female	29	0
78	Female	43	2
79	Female	57	2

80	Female	36	2
81	Female	24	6
82	Female	52	4
83	Female	25	10
84	Female	48	13
85	Female	29	6
86	Male	26	12
87	Female	44	0
88	Female	34	11
89	Female	24	5
90	Female	27	2
91	Female	20	4
92	Female	25	4
93	Female	28	4
94	Female	37	2
95	Female	44	12
96	Female	56	2
97	Female	39	6
98	Female	51	10
99	Female	32	4
100	Female	31	6
101	Female	28	10
102	Female	27	6
103	Female	33	10
104	Female	46	6
105	Female	45	2
106	Female	48	10
107	Female	31	12
108	Female	27	8
109	Female	69	6
110	Female	33	0
111	Female	37	8
112	Female	16	4
113	Female	47	8
114	Female	23	2
115	Female	20	2
116	Female	14	2
117	Female	21	8
118	Female	31	2
119	Female	48	12
120	Female	34	4
121	Female	54	9
122	Female	48	4
123	Female	32	6
124	Female	21	6
125	Female	31	2
126	Female	20	2

127	Female	38	6
128	Female	49	0
129	Female	53	8
130	Female	38	2
131	Female	38	0
132	Female	35	2
133	Female	45	5
134	Female	28	2
135	Female	48	0
136	Female	50	0
137	Female	19	8
138	Female	39	18
139	Female	26	12
140	Female	50	19
141	Female	24	4
142	Female	36	16
143	Female	27	12
144	Female	21	10
145	Female	38	16
146	Female	30	28
147	Female	28	25
148	Female	32	6
149	Male	48	12
150	Female	36	12
151	Female	18	16
152	Female	28	3
153	Female	38	2
154	Female	52	6
155	Female	49	4
156	Female	18	8
157	Female	53	4
158	Female	51	2
159	Female	25	4
160	Female	49	5
161	Female	33	4
162	Female	33	8
163	Female	38	2
164	Female	53	2
165	Female	50	6
166	Female	30	12
167	Female	35	12
168	Female	35	8
169	Female	42	6
170	Female	48	13
171	Female	34	11
172	Female	24	5
173	Female	27	2

174	Female	44	8
175	Female	26	16
176	Female	53	0
177	Female	28	0
178	Female	24	18
179	Female	47	4
180	Female	15	2
181	Female	33	6
182	Female	31	6
183	Female	42	2
184	Female	50	8
185	Female	27	8
186	Female	34	0
187	Female	33	6
188	Female	23	5
189	Female	63	6
190	Female	31	0
191	Female	37	8
192	Female	57	8
193	Female	58	4

No.1-No.61 were used to determine the intracellular Fe^{2+} in $CD4^+$ T cells (Figure 1, A and B). No.62-No.104 were used to determine the mRNA expression of *FTH* and *FTL* (Figure 1, C and D). No.105-No.108 were used to determine the protein level of ferritin (Figure 1E). No.109-No.136 were used to analyze the correlation between intracellular Fe^{2+} and Tfh cell frequency in $CD4^+$ T cells (Figure 1F). No.137-No.154 were used to determine the expression of miR-21 and Tfh cell-related gene *CXCR5*, *PDCD1*, *BCL6*, and *IL21*, and to analyze the correlation between miR-21 and Tfh cell-related genes and SLEDAI score (Figure 7, A-G). No.155-No.169 were used to detect the percentage of Tfh cells in lupus $CD4^+$ T cells (Figure 7H). No.170-No.173 were used for Antagomir-21 treatment (Figure 7, I-K). No.174-No.193 were used to determine the intracellular $Fe2^+$ in Th cell subsets of SLE patients (Supplemental Figure 1).

Supplemental Table 2. Reagents

Reagents	Catalog number	Brand
FITC Rat anti-mouse CD4	100406	Biolegend
PE-Cy™7 Rat Anti-Mouse CD185 (CXCR5)	560617	BD Pharmingen
PE Hamster Anti-Mouse CD279 (PD-1)	551892	BD Pharmingen
APC Hamster Anti-Mouse CD279 (PD-1)	562671	BD Pharmingen
APC Rat anti mouse FOXP3	17-4776-42	eBioscience
Zombie NIR™ Fixable Viability Kit	423106	Biolegend
FITC Rat Anti-Mouse T- and B-Cell Activation Antigen (GL-7)	553666	BD Pharmingen
PE Hamster Anti-Mouse CD95	554258	BD Pharmingen
APC Rat Anti-Mouse CD45R/B220	553092	BD Pharmingen
PE/Cyanine7 anti-mouse CD138 (Syndecan-1)	142514	Biolegend
PE Rat anti-mouse IL-21	12-7211-82	eBioscience
APC Rat anti-mouse IL-4	17-7041-82	eBioscience
APC-Cy™7 Rat Anti-Mouse IL-17A	560821	BD Pharmingen
PerCP-Cy™5.5 Rat Anti-Mouse IFN-γ	560660	BD Pharmingen
Cytofix/Cytoperm™ Fixation/Permeabilization Solution Kit	554714	BD Pharmingen
Foxp3 / Transcription Factor Staining Buffer Set	00-5523-00	eBioscience
Leukocyte Activation Cocktail, with BD GolgiPlμg™	550583	BD Pharmingen
Alexa Fluor 488 Rat anti-mouse CD25	53-0251-82	eBioscience
PerCP-Cyanine5.5 Rat anti-mouse CD4	35-0042-80	eBioscience
PE-Cy™7 Rat Anti-Mouse CD44	560569	BD Pharmingen
APC Hamster Anti-Mouse CD3e	561826	BD Pharmingen
PerCP/Cyanine5.5 anti-mouse CD8a	100734	Biolegend
APC-Cy™7 Rat Anti-Mouse CD62L	560514	BD Pharmingen
PE Rat anti-Ki67	12-5698-82	eBioscience
FITC Annexin V Apoptosis Detection Kit I	556547	BD Pharmingen
Reactive Oxygen Species Assay Kit	S0033S	Beyotime
FerroOrange	F374	DOJINDO
FITC Mouse Anti-Human CD4	555346	BD Pharmingen
PE-Cyanine7 Mouse Anti-human CD185 (CXCR5)	25-9185-42	eBioscience
APC Mouse anti-human CD279 (PD-1)	329908	Biolegend
BB515 Mouse anti-human CD196 (CCR6)	564479	BD Pharmingen
BV421 Mouse anti-human CD25	564033	BD Pharmingen
BV480 Mouse anti-human CD194 (CCR4)	746361	BD Pharmingen
BV510 Mouse anti-human CD45RA	563031	BD Pharmingen
BV605 Mouse anti-human CD45RO	562791	BD Pharmingen
BV650 Mouse anti-human CXCR3	740603	BD Pharmingen
BV786 Mouse anti-human CD127	563324	BD Pharmingen
Pacific Blue Rat anti-mouse/human GL-7	144614	Biolegend
BV711 Hamster anti-mouse CD95	740716	BD Pharmingen
BV786 Rat anti-mouse CD45R/B220	563894	BD Pharmingen
APC-Cy7 Rat anti-mouse CD44	560568	BD Pharmingen
PE-Cy™7 Hamster anti-mouse CD11c	561022	BD Pharmingen
FITC Rat anti-mouse CD11b	11-0112-82	eBioscience
APC Rat anti-mouse F4/80	123116	Biolegend
PerCP/Cyanine5.5 anti-mouse CD45R/B220	103236	Biolegend
Rabbit Anti-Ferritin monoclonal antibody	ab75973	Abcam
Rabbit Anti-BDH2 polyclonal antibody	27207-1-AP	Proteintech
Rabbit Anti-BCL6 monoclonal antibody	14895S	CST

Rabbit Anti-TET2 polyclonal antibody	21207-1-AP	Proteintech
Rabbit Anti-TET3 polyclonal antibody	Ab139311	Abcam
Rabbit Anti-Lamin B1 monoclonal antibody	13435S	CST
Mouse Anti-Beta Actin monoclonal antibody	HRP-60008	Proteintech
Goat anti-mouse IgG (H+L), HRP conjugate	SA00001-1	Proteintech
Goat Anti-Rabbit IgG(H+L), HRP conjugate	SA00001-2	Proteintech
Goat Anti-Mouse IgG1	A90-105P	Bethyl laboritories
Goat Anti-Mouse IgG2b	A90-109P	Bethyl laboritories
Goat Anti-Mouse IgG3	A90-111P	Bethyl laboritories
Goat Anti-Mouse IgG2a	A90-107P	Bethyl laboritories
Goat Anti-Mouse IgM	1020-05	SouthernBiotech
Mouse Anti-dsDNA IgG ELISA Kit	5120	Alpha diagnostic
Mouse Anti-Nuclear Antibodies (ANA) Total Ig ELISA Kit	5210	Alpha diagnostic
5%SRBCs	RCB001	Bersee
Rabbit Anti-Mouse C3	ab200999	Abcam
Rabbit Anti-Mouse CD3	ab5690	Abcam
Peanut Agglutinin (PNA), Unconjugated	L-1070	Vector Labs
Biotinylated anti-Peanut Agglutinin	BA-0074	Vector Labs
Donkey Anti-Mouse IgG H&L (HRP)	ab205724	Abcam
Donkey Anti-Rabbit IgG H&L (HRP)	ab205722	Abcam
Rat Anti-Mouse CD45R(B220)	ab64100	Abcam
Goat Anti-Rat IgG(H+L), HRP conjugate	SA00001-15	Proteintech
Opal 7-Color Manual IHC Kit	NEL811001KT	Perkin Elmer
Total Iron-Binding Capacity (TIBC) and Serum Iron Assay	ab239715	Abcam
Colorimetric Iron Assay	ab83366	Abcam
Pristane	P9622	Sigma
Iron dextran	D8517	Sigma
2,5-dihydroxybenzoic acid(2,5-DHBA)	S3799	Selleck
Ciprofloxacin (CPX)	S5008	Selleck
Human anti-CD3	217570	Calbiochem
Human anti-CD28	217669	Calbiochem
Human anti-IFN-γ	16-7317-85	eBioscience
Human anti-IL-4	16-7048-85	eBioscience
Recombinant human IL-6	200-06-5	PeproTech
Recombinant human IL-12	200-12-2	PeproTech
Recombinant human IL-21	8879-IL-010	R&D Systems
Recombinant human TGF-β	240-B-002	R&D Systems
Recombinant human IL-2	200-02-10	PeproTech
Recombinant human IL-1β	200-01B-2	PeproTech
Recombinant human IL-23	200-23-2	PeproTech
Purified Hamster anti-mouse CD3e, Functional Grade	16-0031-85	eBioscience
Purified Hamster anti-mouse CD28, Functional Grade	16-0281-85	eBioscience
Purified Rat anti-mouse IFN-γ, Functional Grade	16-7312-85	eBioscience
Purified Rat anti-mouse IL-4, Functional Grade	16-7041-85	eBioscience
Purified Anti-TGF-β, Functional Grade	16-9243-85	eBioscience
Recombinant murine IL-6	216-16	PeproTech
Recombinant murine IL-21	210-21	PeproTech
Recombinant murine IL-12	210-12-2	PeproTech
Recombinant murine IL-4	214-14-5	PeproTech
Recombinant TGF-β	7666-MB-005	R&D Systems
Recombinant IL-1β	211-11B-2	PeproTech

Recombinant IL-23	1887-ML-010	R&D Systems
Recombinant IL-2	212-12-5	PeproTech
Mouse naive $CD4^+$ T cell isolation kit II	130-104-453	Miltenyi Biotec
Human Naive $CD4^+$ T Cell Isolation Kit	130-094-131	Miltenyi Biotec
Human CD4 microbeads	130-045-101	Miltenyi Biotec
Mouse CD4 microbeads	130-117-043	Miltenyi Biotec
Mouse CD19 microbeads	130-121-301	Miltenyi Biotec
TRIzol reagent	TR118	MRC
TB Green® Premix Ex Taq™	RR420Q	Takara
micrOFF hsa-miR-21 antagomir	miR310813164540-4	RiboBio
micrON hsa-miR-21 agomir	miR4101028121559-4	RiboBio
Bulge-Loop hsa-miR-21-5p Primer Set	MQPS0000834-1	RiboBio
Bulge-Loop U6 qPCR Primer Set	MQPS0000002-1	RiboBio
M-MLV Reverse Transcriptase	M1701	Progema
Recombinant Rnasin Ribonuclease inhibitor	N251A	Progema
dNTP Mix	U151A	Progema
PrimeScript RT reagent Kit With gDNA Eraser (Perfect Real Time)	RR047A	Takara
Falcon 70um cell strainer, white	352350	Falcon
Nuclear and Cytoplasmic Protein Extraction Kit	P0028	Beyotime
5mC Hydroxylase TET Activity/Inhibition Assay Kit (Fluorometric)	P-3087	Epigentek
JMJD3/UTX Demethylase Activity/Inhibition Assay Kit	P-3085	Epigentek
P3 Primary Cell 4D-Nucleofector® X Kit L (24 RCT)	V4XP-3024	Lonza
QIAamp Fast DNA Tissue Kit	51404	Qiagen
Magnetic Methylated DNA Immunoprecipitation Kit	C02010040	Diagenode
DNA Clean & Concentrator-5	D4014	ZYMO
MeDIP	55009	Active Motif
hMeDIP	55010	Active Motif
RIPA Lysis Buffer	20-188	Sigma
PMSF (100mM)	ST506	Beyotime
Pierce™ BCA Protein Assay Kit	23225	Thermo Scientific
Mouse Direct PCR Kit	B40015	Bimake

Supplemental Table 3. Primer sequences

Mouse *Bcl6* FP	TATCCAGTTTACCCGGCACG
Mouse *Bcl6* RP	TGTCGACAACATGCTCCATCT
Mouse *Cxcr5* FP	AGGCACCAGCACAAACCTTC
Mouse *Cxcr5* RP	AGGCCAGTTCCTTGTACAGGTC
Mouse *Pdcd1* FP	TGGGGCCTAAGCCTATGTCT
Mouse *Pdcd1* RP	CTCCCAAGGGTGGCTTTAGG
Mouse *Il4* FP	TCACAGCAACGAAGAACACCA
Mouse *Il4* RP	CAGGCATCGAAAAGCCCGAA
Mouse *IL21* FP	ACTCAGTTCTGGTGGCATGG
Mouse *Il21* RP	TGCTCACAGTGCCCCTTTAC
Mouse *Tfrc* FP	TTCGCAGGCCAGTGCTAGG
Mouse *Tfrc* RP	TACAAGGGAGTACCCCGACAG
Mouse *Fth* FP	CAGACCGTGATGACTGGGAG
Mouse *Fth* RP	TCAATGAAGTCACATAAGTGGGGA
Mouse *Slc40a1* FP	TCCAACCCGCTCCCATAAG
Mouse *Slc40a1* RP	CAGCCTTATGCCGAAAGACC
Mouse *Gapdh* FP	AGGTCGGTGTGAACGGATTTG

Mouse *Gapdh* RP	TGTAGACCATGTAGTTGAGGTCA
Mouse *Actb* FP	GTGACGTTGACATCCGTAAAGA
Mouse *Actb* RP	GCCGGACTCATCGTACTCC
Human *TET2* FP	GATAGAACCAACCATGTTGAGGG
Human *TET2* RP	TGGAGCTTTGTAGCCAGAGGT
Human *TET3* FP	TCCAGCAACTCCTAGAACTGAG
Human *TET3* RP	AGGCCGCTTGAATACTGACTG
Human *FTL* FP	CAGCCTGGTCAATTTGTACCT
Human *FTL* RP	GCCAATTCGCGGAAGAAGTG
Human *FTH* FP	GCCTCCTACGTTTACCTGTCCA
Human *FTH* RP	GGAAGATTCGGCCACCTCGT
Human *IL21* FP	TGGTCCCTGAATTTCTGCCAG
Human *IL21* RP	TTAGTTGGGCCTTCTGAAAGCA
Human *BCL6* FP	CGGAAGGGTCTGGTTAG
Human *BCL6* RP	TGAGCACGATGAACTTGTAT
Human *ACTB* FP	GAGCTACGAGCTGCCTGACG
Human *ACTB* RP	GTAGTTTCGTGGATGCCACAG
Human *CXCR5* FP	CTACCCGCTAACGCTGGAAA
Human *CXCR5* RP	GGTATTGTCCACGGCCTTCA
Human *PDCD1* FP	TGGATTTCCAGTGGCGAGAG
Human *PDCD1* RP	TGACCTTGGGACCGTAGGAT
Human *BDH2* FP	TTCATCCAGCAGGGCATCAG
Human *BDH2* RP	CAAGCTCCAGCCTCCATCAA
Human *CXCR5* promoter FP	GAGGAAATGCCCACTTCTGGA
Human *CXCR5* promoter RP	GAGGGCCGCTTCCTTTAAGA
Human *BCL6* promoter FP	CCGATGCCGATCCTGTGATT
Human *BCL6* promoter RP	TTGCCAAGCAGTTTTCGGTG
Human *PDCD1* promoter FP	GGCAGCTTAGCCACTTATCCA
Human *PDCD1* promoter RP	CAGACAGCTAAGGTGGCCC
Human *IL21* promoter FP	CCTAAAAGGTAGCCTCTCCTCC
Human *IL21* promoter RP	TGGCAACATGTGCTTTCATGC

Supplemental Table 4. Down-regulated genes in miR-21 cKO Tfh cells

Gene.Name	miR21_cKO1	miR21_cKO2	WT1	WT2	log2FC	pvalue	padj
Phc3	541	399	1329	2379	-2.24983	2.55E-19	1.41E-15
Fcho2	352	292	783	1072	-1.80368	1.74E-15	6.44E-12
mt-Co1	1237267	824582	2170020	2128033	-1.34969	5.85E-12	1.30E-08
Lrrc47	227	200	560	547	-1.66169	7.16E-12	1.32E-08
Gm29216	36931	23882	66605	61052	-1.36221	3.41E-11	4.73E-08
Cish	3321	2878	4979	5936	-1.09757	6.01E-11	6.67E-08
mt-Atp6	244300	171954	405961	390836	-1.22684	7.58E-11	7.64E-08
mt-Nd4	142860	101792	227019	228605	-1.18563	1.26E-10	1.16E-07
Gatad2b	344	469	807	1182	-1.56216	1.37E-10	1.17E-07
Gm10925	41003	28745	68787	64442	-1.2247	1.58E-10	1.25E-07
Gm28439	13974	10043	22780	22096	-1.19154	1.70E-10	1.26E-07
Zfp52	1084	1044	2084	1858	-1.17918	4.55E-10	3.16E-07
Gm13340	8604	5603	14912	13450	-1.29018	5.24E-10	3.42E-07
mt-Atp8	84801	57013	138562	129914	-1.21213	8.40E-10	4.91E-07
mt-Co2	187765	135005	292230	284301	-1.12647	1.05E-09	5.84E-07
mt-Nd4l	29881	20704	45826	44897	-1.1324	2.90E-09	1.47E-06

Gm28661	30596	21278	47841	45534	-1.13858	2.92E-09	1.47E-06
Gm10222	5721	4110	9146	8511	-1.1358	3.44E-09	1.66E-06
mt-Co3	200285	144283	308245	292085	-1.09139	4.47E-09	2.06E-06
mt-Cytb	168762	122023	284431	240491	-1.14625	5.06E-09	2.24E-06
Gm28437	31609	22743	49423	45701	-1.09869	5.90E-09	2.52E-06
Inpp4a	160	111	333	357	-1.63445	2.52E-08	1.00E-05
Inpp4b	1155	1039	1734	1776	-0.96379	1.04E-07	3.71E-05
Apc	483	808	1314	1330	-1.3156	1.07E-07	3.71E-05
A630033H20Rik	190	123	386	363	-1.54952	1.55E-07	5.05E-05
mt-Nd5	112213	83252	137633	170120	-0.93634	2.06E-07	6.36E-05
mt-Nd1	104055	77655	137079	141937	-0.90593	2.77E-07	8.31E-05
mt-Nd2	74089	53238	93793	105372	-0.93029	3.01E-07	8.78E-05
Lrrk2	111	93	312	211	-1.65691	1.30E-06	0.000335
Zkscan3	2286	1525	3332	2995	-1.02396	1.53E-06	0.000383
Gm8995	8036	6056	9691	11302	-0.85827	1.55E-06	0.000383
Clk1	3263	2903	4117	4583	-0.78004	2.17E-06	0.000501
Btla	4047	3114	5280	5295	-0.85037	2.47E-06	0.000548
Suv420h2	391	222	634	596	-1.2967	3.04E-06	0.000649
Hdac9	94	87	307	175	-1.716	4.10E-06	0.000843
Gm37881	434	287	737	604	-1.19038	5.53E-06	0.001097
Asxl2	712	487	872	1180	-1.05449	6.42E-06	0.001249
Dnmt3a	487	857	1197	1261	-1.15063	7.11E-06	0.001293
Mapk1ip1	113	141	244	292	-1.35613	9.04E-06	0.001543
Cpd	467	351	768	635	-1.0722	1.14E-05	0.001891
Itga3	2191	1650	2937	2694	-0.84285	1.67E-05	0.002718
Zfp800	507	515	731	823	-0.88655	1.76E-05	0.002824
Slc36a1	38	32	93	131	-1.95475	1.99E-05	0.003113
Noct	336	231	781	408	-1.3768	2.23E-05	0.003429
Lrig1	399	266	519	634	-1.07662	2.46E-05	0.003681
Malt1	5964	5810	7637	7731	-0.67	2.48E-05	0.003681
Gpr83	272	155	489	374	-1.31336	2.49E-05	0.003681
mt-Nd3	6950	4909	7914	8967	-0.79413	2.52E-05	0.003686
Pde7a	1319	908	1719	1666	-0.89388	2.63E-05	0.003794
Ogt	11905	10391	12973	17374	-0.7223	2.73E-05	0.003836
Gm42418	1330	747	1797	1649	-1.02365	3.43E-05	0.004545
Igfbp4	2540	2123	2607	5116	-0.99403	3.80E-05	0.004899
Zfp513	164	164	297	300	-1.14938	4.03E-05	0.005081
Zfp738	294	225	437	430	-1.02847	6.25E-05	0.007381
Zfp53	814	830	1083	1199	-0.75552	6.53E-05	0.007549
Atxn1	695	769	1134	989	-0.82541	8.12E-05	0.008926
6430571L13Rik	816	618	1064	1028	-0.83389	8.81E-05	0.009223
Zfp862-ps	118	73	260	172	-1.47897	9.45E-05	0.009712
Sspo	11	3	59	39	-3.11125	9.79E-05	0.00988
Pdcd1	343	297	714	421	-1.12955	0.000117	0.01148
Pnisr	3266	2860	4023	3888	-0.65695	0.000129	0.012358
Tnfsf11	1565	1397	2389	1802	-0.79643	0.000157	0.014558

Trps1	450	532	625	843	-0.85527	0.000165	0.015004
Tex15	352	248	551	439	-1.01785	0.000177	0.015857
Slc15a3	435	364	864	502	-1.07752	0.000179	0.015857
Tmc6	886	636	1218	1022	-0.85149	0.000192	0.016882
Hectd2	375	472	640	628	-0.86664	0.000196	0.017116
Ikbke	3581	2207	4239	4045	-0.80863	0.000215	0.018331
Cdc14b	227	185	297	388	-1.01263	0.000224	0.018562
Abca5	18	15	55	72	-2.22312	0.000224	0.018562
Clk4	1205	1117	1498	1533	-0.67008	0.000224	0.018562
Izumo1r	1927	1659	2220	2370	-0.64107	0.000257	0.020841
C230085N15Rik	110	62	205	165	-1.40133	0.000281	0.022431
Fam193a	537	446	710	686	-0.79425	0.000302	0.023793
Gm36989	73	40	151	121	-1.56362	0.000327	0.025337
Gm36931	234	237	437	329	-0.99577	0.000343	0.025895
Mapkbp1	1449	834	1621	1748	-0.84925	0.000359	0.026886
Gm37123	47	30	103	99	-1.68114	0.000362	0.026922
AI504432	1406	1005	1714	1593	-0.74675	0.000371	0.027454
Cbarp	483	386	617	622	-0.79894	0.00043	0.030948
Ccdc137	331	195	444	420	-1.00741	0.000478	0.033588
Prpf39	1983	1662	2253	2346	-0.62137	0.000488	0.033861
Cdip1	434	366	535	600	-0.78813	0.000494	0.033966
Cd6	4133	3056	5186	4349	-0.7015	0.000497	0.033966
Wdr81	163	227	261	419	-1.071	0.000505	0.033966
Srgap3	594	1003	1234	1167	-0.87167	0.000535	0.034941
Lnpep	1698	1435	2011	1963	-0.63099	0.000578	0.037488
Itm2a	842	871	1051	1163	-0.65246	0.000581	0.037488
Cd200	1739	1837	2941	2008	-0.76627	0.000617	0.039571
Gm28438	1113	714	1219	1341	-0.773	0.000621	0.03959
Lgals4	536	393	636	681	-0.78953	0.000633	0.040145
Gramd1a	6554	4622	7266	7114	-0.65313	0.000673	0.042163
Ankrd12	954	726	1088	1138	-0.69253	0.000764	0.04657
Gprin3	819	547	840	1099	-0.78592	0.000785	0.047341
Cpt1b	21	1	52	69	-2.74812	0.000812	0.048469
Gm37105	11	12	58	38	-2.35878	0.00084	0.049532
Pik3cd	4981	3347	4788	6212	-0.6822	0.000853	0.049792
Ccnt2	1160	941	1343	1353	-0.64697	0.000865	0.050234
Zfp831	240	148	305	332	-1.00193	0.000872	0.050402
Pacsin1	133	76	164	240	-1.22913	0.000894	0.050976
Hivep3	792	438	1092	809	-0.92806	0.000908	0.050976
Zbtb20	140	192	267	279	-0.99974	0.000909	0.050976
Zfp407	399	349	617	473	-0.83809	0.00091	0.050976
Sacs	1786	1308	1977	2013	-0.6547	0.000919	0.050976
Socs3	2406	1575	2238	3267	-0.74483	0.000919	0.050976
Faah	1261	820	1318	1524	-0.73429	0.000926	0.051094
Phf20l1	2114	1769	2245	2541	-0.58498	0.000955	0.052206
Mis12	202	70	278	276	-1.31979	0.000989	0.052775

Cd5	5667	4465	7135	5796	-0.64649	0.001004	0.053256
Mbd5	416	261	547	481	-0.89591	0.001013	0.053256
Zcchc7	1435	1153	1662	1620	-0.63112	0.001018	0.053256
Pgap2	199	235	340	321	-0.89309	0.001068	0.055652
Slamf6	641	285	711	792	-0.98739	0.001114	0.057494
Zfp948	800	587	1008	880	-0.73737	0.001124	0.057518
Arid5b	472	312	559	576	-0.82177	0.001125	0.057518
Arhgap27	937	726	1048	1111	-0.66254	0.001173	0.058931
Gm20716	465	227	587	531	-0.9865	0.001176	0.058931
Skil	267	283	417	380	-0.82316	0.001179	0.058931
Trim30d	640	573	934	724	-0.74538	0.001201	0.059514
Pde1b	683	427	809	773	-0.80208	0.001214	0.059711
Trp53i11	330	301	365	574	-0.84622	0.00122	0.059711
Hspa1b	8	8	21	64	-2.6645	0.001231	0.059711
Slc17a9	911	744	1001	1122	-0.64308	0.001231	0.059711
Per1	664	355	712	815	-0.86997	0.001351	0.06406
Mdm4	6014	4917	6426	6623	-0.54208	0.001414	0.065832
Kdm7a	959	803	985	1288	-0.6462	0.001447	0.066251
Ccnl2	5770	4486	5749	6673	-0.55956	0.001507	0.067713
Tbx6	73	60	123	136	-1.24554	0.001558	0.069437
Gm37248	14	6	63	27	-2.48427	0.001713	0.074854
Rgs2	667	640	1006	753	-0.72357	0.001759	0.076246
Zfp280d	588	491	585	914	-0.74775	0.001771	0.076267
Adamts6	342	207	363	479	-0.89805	0.001773	0.076267
Tor1b	306	233	573	307	-1.01392	0.001829	0.077896
RP23-20K24.9	2	10	38	32	-2.82784	0.001853	NA
Gm29253	1	0	7	36	-5.67231	0.001944	NA
Nisch	3735	2418	3836	4028	-0.64184	0.001954	0.081514
Rbm5	3784	2897	3738	4366	-0.56163	0.002016	0.083183
Abca1	946	745	1100	1052	-0.63687	0.00204	0.083828
Orai1	360	406	669	446	-0.83881	0.002051	0.083945
Rgs1	3018	2773	3309	3457	-0.50961	0.002077	0.084093
Ip6k1	1894	1311	1869	2206	-0.63001	0.002156	0.086036
Rc3h2	1102	936	1164	1348	-0.58417	0.00218	0.086701
Mga	1219	1047	1322	1440	-0.56988	0.002191	0.086706
Ckmt1	85	62	150	127	-1.20678	0.002217	0.086706
Prpf4b	2580	2249	2711	2980	-0.52089	0.002219	0.086706
Golim4	173	158	268	239	-0.90483	0.00225	0.087269
Klf3	135	89	185	197	-1.05682	0.00225	0.087269
Fam208b	1765	1460	1875	2011	-0.55411	0.002268	0.0874
Ccr6	50	44	102	96	-1.36299	0.002308	0.088021
Zscan26	993	870	1102	1184	-0.57977	0.00233	0.088548
Zfp707	61	56	131	102	-1.28731	0.002344	0.088759
Gm1966	443	293	402	723	-0.88319	0.002399	0.089614
Angptl2	22	9	63	43	-2.07588	0.002502	0.092548
Zfp942	674	560	819	762	-0.64691	0.002613	0.09575

Serinc5	1128	754	1177	1263	-0.66141	0.002669	0.097086
Zkscan1	629	501	697	756	-0.64771	0.002706	0.098083
Flnb	1222	804	1273	1354	-0.662	0.002723	0.098083
Pard6b	40	24	71	85	-1.56907	0.00274	0.098174
Zfp712	62	47	112	106	-1.2891	0.002748	0.098174
1810026B05Rik	2126	1739	1997	2784	-0.58375	0.002759	0.098174
Kdm1b	461	395	510	616	-0.67667	0.00276	0.098174
Spry1	703	589	907	763	-0.66275	0.002828	0.099752
5830444F18Rik	16	10	52	39	-2.10416	0.00285	0.099752
Gm36995	63	48	138	90	-1.33845	0.002886	0.100714
Ankrd23	333	241	337	499	-0.81874	0.002934	0.101739
Nfkbiz	1354	1062	1356	1613	-0.57964	0.002946	0.101826
Prkca	1125	788	1154	1281	-0.63344	0.002968	0.102262
Gm37726	9	5	42	25	-2.56252	0.003044	NA
Bmf	13	13	31	62	-2.10459	0.003065	0.104558
Ftx	291	230	356	373	-0.77066	0.003072	0.104558
Rad52	400	429	566	522	-0.67983	0.003173	0.106352
Rassf1	1125	842	1125	1335	-0.60539	0.003199	0.106546
Tnfsf13b	0	0	6	16	-6.1602	0.003199	NA
Spopl	487	349	518	607	-0.71178	0.003207	0.106546
Gm38394	3179	3124	3527	3690	-0.47988	0.003281	0.108363
Samd9l	1425	1033	1197	2191	-0.73247	0.003322	0.10909
Paxbp1	5225	4562	5307	5898	-0.47878	0.003323	0.10909
Cd55	230	259	361	327	-0.78029	0.003465	0.112301
Dmtf1	1341	1102	1426	1510	-0.55076	0.003475	0.112301
Kcnq1ot1	296	169	378	329	-0.89868	0.003574	0.114921
Gm43484	11	16	44	46	-2.01904	0.00359	0.114921
Gga3	449	328	645	453	-0.79795	0.003617	0.115101
Rbm38	619	341	706	655	-0.79635	0.003656	0.115903
Tnfsf8	5934	6159	8055	6392	-0.54961	0.003695	0.116819
Vps39	959	886	1061	1153	-0.54682	0.003722	0.117326
Pou2f2	748	408	768	845	-0.76814	0.003772	0.118246
Gm26917	1653	1333	1809	1765	-0.54766	0.003829	0.119003
Stom	76	71	135	126	-1.11631	0.003891	0.120255
Pmepa1	128	106	193	180	-0.96168	0.003907	0.120428
Zfp422	49	77	55	268	-1.59861	0.003941	0.121124
Atp10d	791	641	799	990	-0.60189	0.004003	0.122707
Ifih1	814	599	991	833	-0.66199	0.004026	0.12272
Rora	541	523	569	806	-0.64527	0.004071	0.123433
Tmc8	613	375	710	642	-0.74527	0.004092	0.123729
Galnt14	4	8	36	24	-2.61569	0.004181	NA
Mapk8ip3	2467	1968	2902	2455	-0.56554	0.004195	0.126158
Rbm4	249	352	459	391	-0.78804	0.004235	0.126663
Heg1	345	414	500	498	-0.67934	0.00432	0.128502
Ephx1	915	834	1115	1011	-0.57115	0.004364	0.129399
Rgs16	958	909	1639	923	-0.76059	0.004382	0.129399

Col11a2	580	425	711	610	-0.68735	0.004393	0.129399
Dtx3	1232	1142	1565	1314	-0.57015	0.004414	0.129578
Kcna3	396	229	457	437	-0.80772	0.004712	0.134069
Calcoco1	385	362	444	521	-0.65075	0.004722	0.134069
Xndc1	487	260	507	563	-0.80576	0.004813	0.135891
Uvssa	719	610	778	849	-0.5762	0.004924	0.13763
Mib1	578	548	598	829	-0.61792	0.005045	0.139772
Sh3bp5l	225	118	289	253	-0.95462	0.005074	0.140065
Jmjd6	823	843	960	1026	-0.53696	0.005184	0.142736
Arhgef18	383	207	337	559	-0.87773	0.005569	0.149991
Axl	1289	1102	1793	1238	-0.64093	0.005614	0.150818
Sdc1	113	98	228	131	-1.06969	0.005631	0.150933
Trat1	305	342	505	377	-0.74068	0.005651	0.151098
Egfr	69	86	183	102	-1.17975	0.005719	0.152171
Trim24	1369	1151	1456	1498	-0.51553	0.005772	0.153233
Pml	852	495	881	903	-0.69468	0.005804	0.153427
Atf2	752	515	715	916	-0.64539	0.005821	0.153427
Cd226	442	362	532	501	-0.65062	0.005851	0.153856
Ddr1	703	361	673	814	-0.76791	0.005969	0.155831
Gm29488	0	2	20	12	-4.29392	0.005969	NA
Ttc14	2357	2099	2242	2936	-0.49483	0.006061	0.157688
9930111J21Rik2	744	585	754	881	-0.58177	0.006188	0.160431
Adprhl2	362	253	415	414	-0.71928	0.006246	0.160917
Rcor3	387	261	385	500	-0.73045	0.006248	0.160917
Timp1	371	322	642	357	-0.83227	0.006265	0.160917
Tchp	262	192	293	338	-0.75865	0.006417	0.164277
Sntb1	46	22	71	83	-1.46517	0.006563	0.166363
Cic	2012	1727	1983	2305	-0.47993	0.006686	0.167845
Gm26621	33	16	81	46	-1.68067	0.006756	0.168206
Gm16973	152	153	214	227	-0.81579	0.006796	0.168321
Rusc1	154	336	360	401	-0.91165	0.006847	0.169198
Gm38190	63	56	90	128	-1.14958	0.006933	0.170087
Rab40c	179	131	235	221	-0.84651	0.007002	0.170759
Crk	615	616	805	702	-0.58176	0.007119	0.172787
Spin4	156	208	271	253	-0.81119	0.007132	0.172787
Eno2	397	235	391	489	-0.76045	0.007212	0.172829
Fosl2	2561	2113	2338	3125	-0.50319	0.007244	0.173236
Epm2aip1	946	873	1076	1058	-0.51732	0.007293	0.174038
Znrf3	324	269	339	448	-0.68705	0.007359	0.175225
Map2k6	124	95	179	163	-0.93334	0.007377	0.175276
Farp1	334	343	467	408	-0.65966	0.007462	0.176538
Prdm15	460	385	542	520	-0.61808	0.007549	0.17785
Gm37488	17	2	49	30	-2.36616	0.007572	0.178015
Phf3	872	889	794	1555	-0.68052	0.007615	0.178126
Trim11	728	563	835	757	-0.59317	0.007616	0.178126
Mau2	1474	1148	1445	1630	-0.51393	0.007722	0.180011

4932438A13Rik	3461	2840	3277	3873	-0.46447	0.007886	0.18177
Sh2b3	484	339	543	521	-0.66028	0.007925	0.18177
A130071D04Rik	57	30	92	85	-1.31694	0.008026	0.182764
Cdkn1b	1562	1386	1550	1825	-0.47676	0.008046	0.182764
1810013L24Rik	504	444	543	623	-0.58111	0.008054	0.182764
Ptprc	13175	13670	14114	15186	-0.40942	0.008232	0.185979
Gigyf1	1065	647	992	1180	-0.62764	0.008648	0.191532
Tmprss6	78	48	92	139	-1.15141	0.008667	0.191562
Stk11	993	817	1134	1020	-0.54166	0.008711	0.191778
Sltm	2047	2027	2293	2275	-0.45125	0.008831	0.192874
Bdp1	972	844	913	1268	-0.5409	0.008959	0.194021
2610008E11Rik	284	278	334	394	-0.65425	0.00897	0.194021
Cd84	1352	1067	1449	1383	-0.51658	0.009067	0.195734
Impdh1	634	611	678	806	-0.53405	0.009244	0.198663
Dgkk	68	17	368	137	-2.89596	0.009268	0.198663
Papolg	640	614	684	809	-0.53258	0.009274	0.198663
Zfp799	178	127	210	232	-0.82038	0.009297	0.198777
Zfp638	1118	982	1107	1325	-0.49295	0.009415	0.200308
Ttll3	133	110	169	193	-0.85812	0.009423	0.200308
Klhl24	1136	1009	1116	1370	-0.49316	0.009468	0.200574
Araf	1622	1292	1571	1788	-0.48858	0.009508	0.200865
Tcf4	68	82	114	136	-1.01611	0.009622	0.201293
Hspa1a	8	4	12	47	-2.55133	0.009764	NA
Nufip2	1541	1659	1850	1783	-0.46944	0.00978	0.202846
Gm28187	74	101	163	125	-1.00989	0.009799	0.202856
Clec2d	1199	1039	1208	1362	-0.48272	0.009817	0.202856
Gm20683	164	135	233	198	-0.81939	0.009884	0.203092
Vps9d1	172	221	273	270	-0.75052	0.00994	0.203866
Slc5a3	647	618	905	667	-0.60901	0.009979	0.203972
Ppp1r18	1511	1398	1343	2205	-0.55734	0.010076	0.205139
Slc37a3	369	456	572	480	-0.64008	0.010125	0.205764
Erbb2ip	1964	1817	1874	2422	-0.46254	0.010203	0.206976
Pikfyve	701	558	768	747	-0.55542	0.010225	0.207038
Ptger2	582	574	763	641	-0.57158	0.010398	0.209616
Pnpla6	471	342	494	532	-0.62161	0.010436	0.209616
Ahdc1	90	51	113	132	-1.08209	0.010457	0.209616
Trim30a	2935	2539	3248	2927	-0.46421	0.01047	0.209616
Ccnl1	3078	2635	3039	3303	-0.43513	0.010489	0.209616
Map4k2	3111	1842	3180	2897	-0.58822	0.010503	0.209616
Zfp609	759	652	765	901	-0.52156	0.010524	0.209648
Zufsp	452	395	521	518	-0.58165	0.010649	0.211344
Scai	863	587	794	998	-0.58721	0.010685	0.211344
Gm43813	0	0	11	5	-5.75078	0.010709	NA
Safb2	1512	1111	1530	1540	-0.51512	0.010716	0.21158
Dgka	9089	7079	8210	9939	-0.44851	0.010843	0.212381
Gm17173	124	104	232	132	-0.97839	0.010858	0.212381

Sirt2	776	693	866	853	-0.51382	0.010879	0.212381
Hdac5	578	407	535	712	-0.61995	0.011016	0.214443
Hmgcr	3133	2882	3246	3360	-0.42062	0.011166	0.215887
Zfp874a	280	283	350	368	-0.63491	0.011172	0.215887
Whsc1l1	4302	3549	3777	5201	-0.47069	0.011187	0.215887
Klhl42	213	162	259	256	-0.74568	0.01122	0.216083
Ric1	404	335	501	431	-0.62644	0.011526	0.219351
Sesn3	1805	1971	2212	2050	-0.46217	0.011553	0.219351
Ahnak	7599	5318	7509	7309	-0.48781	0.011581	0.219351
B3gnt2	986	935	1342	978	-0.56837	0.011726	0.221658
AW146154	105	98	169	141	-0.90216	0.011848	0.22273
Kcna2	10	3	26	29	-2.36962	0.011849	NA
H2-K2	158	110	176	218	-0.83791	0.011921	0.222953
Tom1l2	300	185	347	318	-0.74737	0.011955	0.222953
Atp8b2	1366	832	1205	1517	-0.59097	0.011973	0.222953
Myh10	245	261	427	271	-0.76294	0.011979	0.222953
Anks3	455	320	516	471	-0.64008	0.011996	0.222953
Malat1	6482	5548	6065	7107	-0.41287	0.012415	0.226735
Gm37940	0	5	27	12	-3.26206	0.012429	NA
Gfi1	730	644	937	727	-0.57088	0.012464	0.226735
Gse1	536	363	448	746	-0.68259	0.012574	0.227596
Lrrc45	421	339	518	438	-0.62348	0.01262	0.228073
Zfp445	1096	857	1040	1235	-0.50254	0.012689	0.228947
Arhgef4	163	113	191	208	-0.81749	0.012983	0.232635
Ttyh3	317	244	317	413	-0.65962	0.013007	0.232635
Ankzf1	859	637	878	898	-0.53495	0.013037	0.232635
Gm6934	25	18	60	43	-1.55758	0.013143	0.234092
AW549877	1616	1487	1560	1922	-0.44614	0.013185	0.234461
Kdm6a	681	698	984	706	-0.58924	0.013241	0.235071
Slc12a9	167	86	173	219	-0.91512	0.013401	0.237537
Casz1	145	95	201	161	-0.88826	0.013523	0.238548
Fpgs	252	227	326	296	-0.66617	0.013605	0.238854
Sugp2	895	714	914	958	-0.5045	0.013636	0.239028
Trp53inp1	7556	6840	6985	8727	-0.40568	0.013694	0.23959
Zfp644	370	279	360	471	-0.63636	0.013711	0.23959
Fnbp4	3073	2416	2902	3222	-0.4424	0.013858	0.241772
Atp6v0a1	109	112	220	126	-0.94844	0.013993	0.243492
Adcy7	1551	1230	1487	1669	-0.46652	0.014003	0.243492
Prpf38b	2264	2189	2721	2293	-0.46274	0.014058	0.243492
Rptor	425	302	446	469	-0.6186	0.0141	0.243492
Plekha7	237	153	235	308	-0.7583	0.014145	0.243492
Cbx7	1757	1182	1562	1910	-0.52308	0.014187	0.243492
Dvl1	1215	739	1209	1181	-0.58113	0.014241	0.243492
Dopey1	743	591	695	894	-0.53214	0.014247	0.243492
Plcxd2	810	550	708	973	-0.58466	0.014251	0.243492
Pitpnm1	2079	1300	1994	2031	-0.54147	0.014274	0.243492

Ebf1	268	212	346	287	-0.6922	0.014286	0.243492
Marf1	1519	1268	1479	1652	-0.45167	0.014403	0.243998
Zfp780b	196	180	244	256	-0.69602	0.014561	0.245178
Nxf1	3131	2579	3123	3177	-0.42875	0.014594	0.24535
1700049G17Rik	73	50	95	115	-1.05439	0.014637	0.245701
Xist	298	9	59042	7	-7.95355	0.014728	0.246863
Prrt2	8	14	16	61	-2.05406	0.015033	NA
Gcfc2	366	227	361	418	-0.67847	0.015568	0.254401
Pan3	970	905	860	1407	-0.54493	0.015759	0.255528
Zbtb18	447	322	555	426	-0.64764	0.01578	0.255528
Tnfrsf14	211	189	248	277	-0.67549	0.015821	0.255528
P2rx7	127	116	213	146	-0.86039	0.015877	0.256067
Kcnn4	2959	2353	2848	3028	-0.43134	0.015973	0.256403
Ldb1	3416	2527	3166	3500	-0.45074	0.015991	0.256403
Rev1	791	650	852	821	-0.5038	0.01616	0.256403
Abca7	2096	1400	1931	2132	-0.5028	0.016191	0.256403
Zfp568	68	41	101	91	-1.10893	0.016274	0.256403
Filip1l	251	292	367	326	-0.63995	0.016294	0.256403
Map3k12	252	195	331	263	-0.70466	0.016337	0.256403
Lrrcc1	492	453	516	604	-0.52667	0.01643	0.257098
Maml1	278	234	272	397	-0.66157	0.016439	0.257098
Vmn2r96	15	33	68	45	-1.52776	0.016526	0.257182
Ypel3	690	818	827	917	-0.49095	0.017004	0.262848
Prickle3	565	387	505	680	-0.5954	0.017269	0.265773
Fam134c	596	526	586	733	-0.51323	0.017342	0.26652
Ncln	820	513	760	870	-0.57545	0.017398	0.266648
Zfp839	163	104	191	193	-0.81287	0.017462	0.267255
Sbf2	235	229	314	281	-0.64787	0.017714	0.269993
Il2	568	422	762	499	-0.65022	0.017744	0.270079
Ankrd16	490	287	471	527	-0.64753	0.017832	0.270678
Fam214a	370	309	386	445	-0.5742	0.01819	0.274227
Gm42793	1	1	21	4	-3.96971	0.018207	NA
Tiam1	1008	746	1215	897	-0.56569	0.018272	0.274227
Slc41a1	859	647	916	844	-0.51541	0.018731	0.278602
N4bp1	572	474	594	632	-0.51434	0.018854	0.280058
Pcsk7	425	322	464	450	-0.57983	0.018939	0.280184
Tug1	2625	1989	2515	2615	-0.43981	0.018987	0.280184
Zfp607	138	117	187	171	-0.77887	0.018994	0.280184
Krba1	618	351	570	659	-0.62857	0.019195	0.282473
Zc3h11a	651	472	577	783	-0.55512	0.019386	0.283591
Atg2a	742	454	774	701	-0.59525	0.019398	0.283591
Pura	621	524	616	715	-0.49964	0.019644	0.285052
Ephb6	170	99	166	228	-0.83075	0.019665	0.285052
Zfp664	502	434	500	607	-0.52302	0.019678	0.285052
3110002H16Rik	784	693	834	843	-0.46958	0.01976	0.285821
Spen	733	575	754	763	-0.50113	0.019869	0.28588

Ubn2	1531	1391	1523	1674	-0.41334	0.02003	0.287168
Tial1	1368	1247	1316	1572	-0.42421	0.020145	0.288429
Gfod1	369	316	490	367	-0.61883	0.020212	0.289011
Morc3	871	828	914	987	-0.44583	0.020314	0.289346
Rrnad1	309	215	349	323	-0.64965	0.020394	0.28965
Dnah7a	1	0	19	3	-4.79081	0.020467	NA
Ccdc50	2766	2636	3000	2837	-0.39974	0.020473	0.28965
Arid4b	1194	1059	1217	1276	-0.43128	0.020518	0.28965
Prdm2	1033	825	1006	1095	-0.46224	0.020631	0.290879
Plxna3	274	154	267	316	-0.73078	0.02075	0.291714
H2-Ob	107	79	117	165	-0.87816	0.020769	0.291714
Hmha1	9564	6706	8334	9781	-0.43861	0.020904	0.292676
Taok2	1155	826	1063	1219	-0.48824	0.020948	0.292676
Gpr174	304	595	585	602	-0.68058	0.021012	0.292676
Hjurp	2085	2273	2067	2767	-0.42537	0.021074	0.292676
Fam53b	709	528	736	714	-0.5182	0.021083	0.292676
Sh3glb2	628	548	650	701	-0.4846	0.021174	0.292676
Gcc2	363	377	421	462	-0.53746	0.02122	0.292676
Twsg1	1454	1544	1725	1584	-0.43038	0.021325	0.292722
Nt5e	169	129	177	233	-0.73984	0.021368	0.292722
Ankrd52	1344	966	1237	1400	-0.47556	0.021417	0.292722
Ndst3	0	2	13	11	-3.86793	0.02147	NA
Gas2l1	81	71	143	97	-0.95676	0.021599	0.293006
Mical3	151	130	174	210	-0.73173	0.02163	0.293006
Ago3	502	353	508	527	-0.56295	0.021892	0.29516
Fam160a2	533	438	496	660	-0.53014	0.021978	0.295256
Thoc2	2348	1968	2225	2463	-0.4033	0.022002	0.295256
Arap1	867	625	816	917	-0.50075	0.022059	0.295261
Tbc1d23	619	526	626	692	-0.48692	0.022314	0.29768
4632428N05Rik	2348	1248	1876	2540	-0.57759	0.022321	0.29768
Kif1b	694	546	677	756	-0.49291	0.022435	0.298407
Zfp687	300	238	329	340	-0.60081	0.022477	0.298407
Ypel2	110	84	118	172	-0.85659	0.022483	0.298407
Ezh1	796	784	817	955	-0.44649	0.02263	0.299403
Dpp4	1937	1637	1641	2422	-0.45989	0.022639	0.299403
Lcor	180	128	198	220	-0.72558	0.022929	0.3018
Zfp871	833	608	758	919	-0.50108	0.023059	0.302441
Fyb	3073	2711	2636	3806	-0.43059	0.023416	0.305209
Plekha1	249	137	317	224	-0.78776	0.023435	0.305209
Dzip1	255	176	252	310	-0.66513	0.023568	0.305508
Gm37101	0	0	10	3	-5.46093	0.0236	NA
Hivep2	1043	1200	1026	1594	-0.49499	0.023747	0.306488
Htt	2727	1934	2446	2758	-0.44379	0.023808	0.306825
Myo1c	1216	880	1217	1171	-0.47777	0.024047	0.308628
Zfp729a	444	427	495	519	-0.50385	0.024086	0.308628
Brat1	258	172	247	318	-0.67506	0.024128	0.308672

Ppp1cc	395	286	345	513	-0.60952	0.024146	0.308672
Gm37639	15	1	29	32	-2.22478	0.024221	NA
Cmip	710	639	842	709	-0.49317	0.024392	0.310745
Nlgn3	20	5	73	11	-2.08737	0.024584	0.312466
Midn	1170	1048	1225	1213	-0.42339	0.024775	0.31356
Sipa1	1155	800	1099	1144	-0.48573	0.024809	0.31356
Prr14l	1041	825	999	1093	-0.44975	0.024905	0.31356
Extl3	543	403	551	566	-0.52692	0.024965	0.31356
Akt3	691	610	703	767	-0.46021	0.025009	0.31356
Jak2	6946	6173	6827	7002	-0.36206	0.025061	0.313862
Plcb2	1097	813	1111	1060	-0.47444	0.025096	0.313938
Fbxw7	291	268	335	345	-0.56806	0.025231	0.314868
Lrfn1	83	68	129	103	-0.9131	0.025255	0.314868
Rarg	253	225	310	286	-0.60719	0.025326	0.315394
Fbxl20	1695	1255	1498	1806	-0.44583	0.025462	0.31602
Gstt2	970	1048	1143	1092	-0.43389	0.025624	0.31722
Akap10	453	418	533	486	-0.51549	0.025664	0.31722
Nsmf	180	124	249	174	-0.77567	0.025725	0.317516
Abtb1	534	406	532	572	-0.51762	0.025791	0.317974
Tnrc6a	3232	2916	3163	3356	-0.36931	0.026028	0.319649
Usp33	809	817	849	955	-0.43189	0.026175	0.319649
Gm42878	175	233	405	184	-0.83637	0.026175	0.319649
Sema4f	692	528	657	750	-0.48952	0.026206	0.319649
Notch2	1547	842	1379	1493	-0.55383	0.02621	0.319649
Hipk2	301	269	350	342	-0.56698	0.026213	0.319649
Carf	75	85	73	199	-1.02194	0.026401	0.320953
Crebrf	798	791	756	1057	-0.46573	0.026409	0.320953
Gm43511	25	26	54	50	-1.31554	0.02661	0.3227
Lemd2	566	416	563	590	-0.51827	0.026747	0.323293
Esrra	311	241	361	322	-0.59824	0.026819	0.323811
Map4k3	193	158	229	226	-0.66186	0.027028	0.324567
Mast4	74	41	125	73	-1.08931	0.027141	0.325574
Ralgapa2	443	326	472	450	-0.55129	0.027328	0.326879
Tecpr1	2492	1745	2087	2686	-0.45255	0.027364	0.326879
Rhno1	551	469	535	641	-0.48678	0.027426	0.326879
4632404H12Rik	59	44	94	78	-1.03278	0.027482	0.327195
Scg2	0	0	12	1	-5.47939	0.027644	NA
Mast3	1499	930	1425	1395	-0.50569	0.027724	0.328592
Gm11455	8	5	25	21	-2.11652	0.027816	NA
Marchf7	2231	2119	2039	2675	-0.39355	0.028002	0.330542
Plekhg2	1067	639	901	1134	-0.53705	0.028108	0.331089
Zfp653	306	176	351	284	-0.69409	0.028302	0.33234
Pde3b	1526	1519	1528	1745	-0.38581	0.028342	0.33234
Polk	1399	1385	1423	1574	-0.38904	0.028374	0.33234
Npas4	17	10	31	39	-1.65666	0.028392	NA
Rsrc2	1410	1446	1621	1486	-0.40983	0.028433	0.33234

Arid4a	971	1083	983	1322	-0.44197	0.028575	0.333313
Ap1b1	928	799	951	960	-0.43269	0.028703	0.334194
9830147E19Rik	48	64	114	73	-1.03596	0.028914	0.335951
Coro6	8	3	18	25	-2.24821	0.029326	NA
B3gntl1	173	145	207	208	-0.67083	0.029352	0.33856
Enthd2	316	246	406	299	-0.62387	0.029456	0.339397
Axdnd1	0	0	2	11	-5.38642	0.029464	NA
Rgl2	739	458	642	792	-0.54362	0.029617	0.3402
Itpr3	1084	690	952	1113	-0.50362	0.029808	0.342037
Susd6	869	845	927	954	-0.41919	0.029904	0.34247
Zfp526	107	84	118	158	-0.80996	0.03018	0.344173
Gm37864	0	0	6	6	-5.31335	0.03033	NA
Smcr8	992	847	969	1053	-0.42128	0.030377	0.344807
Zc3h7a	3543	2915	3124	3799	-0.38149	0.030391	0.344807
Arfgap1	528	276	567	460	-0.65049	0.030516	0.345495
Mgll	71	73	112	105	-0.87885	0.030598	0.345733
Git1	584	420	644	543	-0.53514	0.030764	0.346761
Gm26532	113	91	127	162	-0.78258	0.030813	0.346761
Sec24a	855	663	734	1009	-0.47733	0.030941	0.347204
Mapk6	1991	1826	1971	2084	-0.372	0.030969	0.347204
Slc4a2	644	455	646	633	-0.50791	0.031201	0.349002
Clstn1	221	168	250	245	-0.63592	0.03124	0.34908
Nfat5	2786	2087	2883	8488	-1.47817	0.031367	0.349792
Hemk1	1644	1158	1515	1606	-0.44235	0.031452	0.350393
Spef1	13	5	17	41	-1.95468	0.031455	NA
Zkscan8	415	373	425	490	-0.49778	0.031537	0.350984
Ythdc1	1413	1274	1367	1522	-0.38781	0.031635	0.351721
Zbtb40	219	148	250	228	-0.67254	0.031745	0.352474
Cbx6	827	690	765	931	-0.44203	0.031805	0.352554
Ash1l	1411	1089	1316	1429	-0.42016	0.031881	0.35305
Zfp459	7	8	19	30	-1.97952	0.032018	NA
Swt1	352	390	399	471	-0.50949	0.032051	0.354224
Plod3	337	305	377	381	-0.5257	0.032226	0.355447
Ppp1r37	508	371	510	520	-0.51625	0.03228	0.355691
Ralgds	305	234	333	324	-0.57408	0.032557	0.357412
Med20	490	500	552	570	-0.46511	0.032565	0.357412
Ube3b	743	665	762	797	-0.43218	0.032608	0.357508
Wdfy1	608	579	740	613	-0.48071	0.032791	0.357508
Srsf5	4385	5166	5140	5017	-0.37401	0.032826	0.357508
Itga5	215	135	241	221	-0.69224	0.032878	0.357508
Szt2	909	549	890	831	-0.53121	0.032893	0.357508
Zbtb7b	1705	1584	1830	1699	-0.39035	0.032896	0.357508
Clk2	1022	747	1041	956	-0.46585	0.032968	0.357598
Cdk17	2269	2178	2250	2434	-0.35855	0.032969	0.357598
Zfp202	60	54	104	78	-0.9695	0.033055	0.357832
Ammecr1l	578	555	588	684	-0.44836	0.033166	0.358295

9230112E08Rik	123	66	131	149	-0.85331	0.033286	0.358295
A430093F15Rik	907	634	915	847	-0.48438	0.033325	0.358295
Tmem259	1677	1216	1610	1586	-0.4326	0.033347	0.358295
Zfp369	247	187	273	269	-0.60875	0.033415	0.358464
Trim26	419	320	369	522	-0.54715	0.033436	0.358464
Zdhhc18	1989	1479	1738	2065	-0.41579	0.03349	0.358696
Gltscr1	398	231	356	435	-0.61437	0.033577	0.359281
Dsp	13	9	52	13	-1.88651	0.033594	NA
Gtf3c1	1422	957	1312	1367	-0.45904	0.03364	0.359334
A930002I21Rik	15	14	37	33	-1.56062	0.033657	NA
Chst2	133	94	196	128	-0.81401	0.033796	0.359334
Ipcef1	744	643	705	840	-0.43713	0.033863	0.359334
Cd28	7999	9244	8693	9399	-0.35144	0.033874	0.359334
Ampd3	44	32	55	81	-1.11618	0.034218	0.361953
Rab43	730	589	687	793	-0.44916	0.034266	0.362113
Pakap	0	0	3	9	-5.28296	0.034328	NA
Spx	5	2	15	18	-2.52203	0.034429	NA
2610005L07Rik	212	244	283	278	-0.58405	0.034535	0.363223
Taz	750	455	643	791	-0.53426	0.034647	0.363794
Ago1	567	490	546	651	-0.46094	0.03471	0.364024
Sft2d1	232	272	354	272	-0.60505	0.035184	0.367128
Zfp748	240	264	302	307	-0.55743	0.035481	0.367128
Spp1	267	457	1620	508	-1.87063	0.035553	0.367314
Bcl6	94	81	150	105	-0.84016	0.035597	0.367423
Csf1	60	43	88	79	-0.98821	0.035698	0.367462
Dlg2	205	248	372	221	-0.68826	0.035766	0.367462
Kdm5a	1854	1670	1742	1995	-0.36703	0.036115	0.369076
Cyp4f13	340	322	403	373	-0.51759	0.036119	0.369076
Zfp317	386	369	440	431	-0.49281	0.036129	0.369076
Srcap	752	406	816	614	-0.60396	0.036169	0.369076
Dgkz	2606	1800	2316	2522	-0.42108	0.036208	0.369076
Gm26740	60	40	61	109	-1.03733	0.036222	0.369076
Zfp54	64	70	111	92	-0.88959	0.036442	0.37089
Zbtb10	75	33	83	99	-1.03896	0.036708	0.372654
Dennd2d	4337	4005	4312	4357	-0.34164	0.036846	0.373377
Eea1	2462	2091	2444	2383	-0.37227	0.036929	0.373868
Utrn	3118	2027	2711	2995	-0.43549	0.037005	0.374163
Rnf111	857	725	767	993	-0.43292	0.03708	0.374163
Macf1	8253	5234	7069	7825	-0.42952	0.037211	0.374531
Arvcf	19	14	38	37	-1.47275	0.037212	0.374531
Arhgef1	7327	4501	5834	7465	-0.45102	0.037291	0.374803
Lmbrd2	220	261	327	267	-0.59481	0.03738	0.375356
Zfp235	76	38	117	73	-1.04125	0.037549	0.376108
Tmem55b	255	243	282	316	-0.54662	0.037748	0.376668
Ccdc64	1406	1228	1236	1629	-0.3995	0.037984	0.378677
Zfp595	187	180	238	221	-0.61076	0.038167	0.378982

Arhgef3	1794	1758	1771	1969	-0.35701	0.038185	0.378982
Sik2	263	253	319	299	-0.54806	0.03823	0.379093
Gm43430	29	27	79	33	-1.31027	0.038497	0.380372
Arhgef19	5	2	16	16	-2.483	0.038911	NA
Aldh4a1	261	209	305	270	-0.58183	0.038944	0.382068
Gm43138	7	8	16	32	-1.94331	0.039104	NA
Luc7l	959	1286	1439	1117	-0.47856	0.03915	0.382739
Ltbp4	5	8	17	27	-2.02854	0.039231	NA
Prkag2	195	149	228	210	-0.63855	0.039288	0.383414
Pofut1	825	715	786	900	-0.41336	0.039373	0.383803
Scrib	1062	669	1001	981	-0.48548	0.039492	0.384053
Rufy2	1057	842	952	1130	-0.41497	0.039492	0.384053
Meis3	293	171	289	300	-0.63276	0.039602	0.384447
Bach2os	0	0	8	3	-5.21491	0.039637	NA
Zfp60	100	153	123	245	-0.80285	0.039689	0.384616
Kmt2e	2093	1533	1866	2065	-0.40165	0.039781	0.385158
Tm9sf1	292	352	368	390	-0.51762	0.039849	0.385158
Vat1l	2	3	15	12	-2.72302	0.039971	NA
Zfp142	484	357	510	468	-0.50841	0.040146	0.385935
4933406I18Rik	6	6	24	17	-2.06809	0.040152	NA
Calcrl	548	695	936	564	-0.57058	0.040271	0.385935
Fam179a	0	0	5	6	-5.18235	0.040381	NA
Fmr1os	0	0	5	6	-5.18235	0.040381	NA
Erlin2	396	376	433	448	-0.47556	0.040517	0.386384
Ttc39b	3487	3226	3446	3516	-0.33837	0.040618	0.386384
Atg9a	389	287	390	406	-0.52253	0.040632	0.386384
Hs3st3b1	106	98	162	121	-0.76686	0.040654	0.386384
Tyk2	973	705	894	973	-0.43967	0.040672	0.386384
Dnajc27	147	75	134	186	-0.80828	0.040822	0.387144
Abi3bp	12	1	17	32	-2.19226	0.040909	NA
Gm42559	29	14	45	45	-1.3559	0.041176	0.388329
RP24-430K15.4	21	18	42	40	-1.36013	0.041188	0.388329
Bahd1	430	369	470	445	-0.48406	0.041209	0.388329
Abcd3	378	416	467	443	-0.48323	0.041295	0.388329
N4bp2l2	1118	1052	1064	1262	-0.38109	0.041351	0.388508
Lancl3	18	14	52	23	-1.53867	0.041434	0.388784
Ogdhl	2	0	11	9	-3.62192	0.041449	NA
Klc1	1268	1029	1291	1200	-0.40673	0.041542	0.388784
Dyrk1b	83	72	110	113	-0.81057	0.04156	0.388784
Dip2c	262	188	260	294	-0.58443	0.04171	0.389039
Gm42702	0	0	4	7	-5.17135	0.041798	NA
Zfp827	170	72	224	136	-0.87994	0.041871	0.389386
Fbxw9	260	184	278	270	-0.59273	0.041935	0.389386
Ly75	1498	1194	1405	1484	-0.38779	0.042257	0.391064
Psd2	47	18	60	63	-1.21064	0.042558	0.392172
Rps6ka3	1328	1098	975	1899	-0.51115	0.042624	0.392172

Insr	607	455	567	635	-0.46315	0.042856	0.39281
Zfp217	788	546	712	797	-0.46299	0.04287	0.39281
Sfi1	538	374	516	538	-0.49603	0.042871	0.39281
Hltf	769	771	899	790	-0.42293	0.042907	0.392815
Arid3b	437	281	402	455	-0.54064	0.042973	0.392866
Itsn2	1424	1341	1380	1538	-0.36056	0.043237	0.39404
Gm13139	105	136	156	166	-0.69992	0.043256	0.39404
Limk2	896	806	911	926	-0.39618	0.043289	0.39404
A230046K03Rik	905	779	794	1063	-0.419	0.043473	0.394171
Gpbp1l1	1207	1108	1237	1227	-0.37668	0.043648	0.394171
Atf7	703	669	704	791	-0.4064	0.043715	0.394171
Ano8	364	272	365	383	-0.52045	0.043717	0.394171
Atrx	4989	4038	4184	5370	-0.36143	0.04373	0.394171
Prkdc	786	689	792	813	-0.40781	0.043854	0.394646
Stard13	98	48	140	88	-0.94747	0.04391	0.394651
Dennd1a	299	219	297	326	-0.5515	0.044267	0.396306
Vav2	1195	829	1076	1165	-0.43316	0.044268	0.396306
Sidt2	2899	1939	2515	2765	-0.41223	0.044319	0.396306
Pdlim5	191	202	229	252	-0.57388	0.044339	0.396306
Zswim8	1735	1139	1503	1682	-0.43389	0.044343	0.396306
Trrap	1764	1264	1496	1812	-0.41004	0.044431	0.396624
Parp3	1655	1221	1868	1361	-0.46536	0.044566	0.397127
Luzp1	112	94	127	153	-0.72423	0.044674	0.397127
Eif4a2	8055	7262	7697	7990	-0.31995	0.044689	0.397127
Nprl2	324	335	369	390	-0.48755	0.044787	0.397127
Pcnxl3	1103	678	948	1084	-0.47567	0.044801	0.397127
Gm37370	22	6	28	43	-1.62316	0.044885	NA
Adarb1	2077	1341	1905	1878	-0.43624	0.044886	0.397127
Zfyve26	467	368	473	479	-0.47632	0.044928	0.397127
Acox3	545	445	513	600	-0.45147	0.044939	0.397127
Bend4	891	790	934	886	-0.40294	0.045171	0.398427
Gna11	321	245	331	339	-0.53034	0.045259	0.398456
Ivns1abp	3217	2878	3114	3196	-0.33593	0.045309	0.398456
Dync2h1	391	282	400	390	-0.52019	0.045404	0.398475
Tmem127	1291	1158	1412	1223	-0.39677	0.045442	0.398475
Zfp788	151	137	196	173	-0.64749	0.045472	0.398475
Sfswap	894	651	828	884	-0.43432	0.0455	0.398475
Rtn4	805	407	759	704	-0.56507	0.045762	0.399827
Kif13b	159	61	153	172	-0.8519	0.046005	0.401522
Gm37607	37	25	71	43	-1.18128	0.04605	0.401522
Prkd2	917	676	791	980	-0.43435	0.046187	0.401608
Rictor	1885	1620	1584	2195	-0.38533	0.046251	0.401608
Gmip	1216	941	1148	1184	-0.39941	0.046276	0.401608
Vcan	3	2	14	12	-2.67071	0.046304	NA
Gucy1a3	15	17	39	32	-1.44018	0.046319	0.401608
Cdk16	721	623	757	716	-0.4208	0.046355	0.401608

Zfp882	62	41	76	87	-0.94677	0.046364	0.401608
Abcc10	190	118	192	209	-0.6673	0.046579	0.402941
Gm340	155	98	161	177	-0.70405	0.046591	0.402941
Gm42742	141	106	290	89	-0.93836	0.046935	0.404292
Chka	178	130	197	196	-0.63967	0.046957	0.404292
Gm42979	34	24	55	51	-1.16003	0.047121	0.404733
Slc29a2	58	66	80	107	-0.869	0.047126	0.404733
Nbeal1	548	512	555	614	-0.42436	0.047296	0.405115
Pou2af1	118	77	124	147	-0.75852	0.047386	0.405115
Rnf169	1220	849	1144	1136	-0.42903	0.04739	0.405115
Adora2a	408	288	443	378	-0.5315	0.04758	0.405801
Gm13251	110	103	136	148	-0.69863	0.048041	0.408222
Pank4	699	483	622	716	-0.46315	0.048042	0.408222
Znrf1	338	342	396	382	-0.48085	0.048406	0.409686
Camk2d	755	601	696	794	-0.41943	0.048423	0.409686
D430042O09Rik	308	206	315	307	-0.56463	0.048621	0.409686
Pou2f1	551	426	502	600	-0.45597	0.048728	0.409686
Tspyl2	652	487	574	707	-0.45112	0.048732	0.409686
Pidd1	612	409	667	523	-0.51743	0.048898	0.410418
Ccng2	1109	1124	997	1445	-0.40317	0.049096	0.411301
Zbtb38	804	790	790	925	-0.38657	0.049114	0.411301
2610021A01Rik	383	346	387	441	-0.46623	0.049241	0.41205
Zfp398	387	284	352	433	-0.50819	0.049498	0.413312
Map3k11	593	465	585	594	-0.44336	0.049535	0.413312
Apaf1	784	788	748	960	-0.39772	0.049708	0.413312
Ralgps1	186	144	161	269	-0.65437	0.049717	0.413312

Supplemental Table 5. Up-regulated genes in miR-21 cKO Tfh cells.

Gene.Name	miR21_cKO1	miR21_cKO2	WT1	WT2	log2FC	pvalue	padj
Plac8	1787	1951	233	384	2.328262	2.63E-24	2.92E-20
Nkg7	1985	3584	596	723	1.803081	1.43E-12	3.97E-09
Klrk1	290	635	57	77	2.515905	3.01E-11	4.73E-08
Tmsb10	18476	18375	6105	8061	1.10235	3.88E-11	4.78E-08
Prdm1	166	271	25	31	2.687741	5.55E-10	3.42E-07
Prf1	261	394	54	80	2.017166	1.17E-08	4.80E-06
Havcr2	126	341	17	39	2.801716	5.65E-08	2.16E-05
Klrc1	212	646	60	82	2.326676	7.13E-08	2.64E-05
Ltb4r1	102	249	17	25	2.793441	1.13E-07	3.79E-05
Vim	14096	20362	5214	7797	1.135309	1.61E-07	5.09E-05
Gpr141	150	476	50	51	2.355539	4.32E-07	0.000123
AA467197	862	1775	395	398	1.453813	6.60E-07	0.000183
EntPD-1	103	252	26	25	2.520021	7.71E-07	0.000209
Ccl4	971	1832	478	412	1.369939	1.10E-06	0.00029
Klrd1	187	395	54	72	1.935676	1.65E-06	0.000399
Ctla2a	924	1799	358	513	1.375144	1.91E-06	0.000451
Serpinb9b	175	426	70	48	2.059939	2.32E-06	0.000524

Il24	978	1775	499	415	1.304193	2.54E-06	0.000552
Rnf220	962	779	348	347	1.037493	3.47E-06	0.000727
Cndp2	2504	3056	1121	1386	0.871655	4.93E-06	0.000995
2810474O19Rik	1238	1012	462	503	0.936597	6.64E-06	0.00127
Smim3	628	1043	292	296	1.225697	6.76E-06	0.001271
Nfkbia	4168	4813	1927	2346	0.793277	7.10E-06	0.001293
Ccdc184	365	623	168	130	1.44067	8.25E-06	0.001453
Irgm1	1381	1851	640	740	0.948915	8.25E-06	0.001453
Txndc5	746	604	270	256	1.07111	8.40E-06	0.001456
Ubc	2139	2530	962	1191	0.839042	1.06E-05	0.001784
Nusap1	2191	2912	845	1322	0.966187	1.86E-05	0.002946
Gm9844	1342	1498	583	730	0.835042	2.70E-05	0.003836
Cks1b	1004	1126	426	531	0.8761	3.02E-05	0.004187
Tnfrsf8	1428	1597	688	740	0.800398	3.12E-05	0.00427
Gpr18	1308	1794	644	725	0.900603	3.16E-05	0.00427
Gzmb	17888	29941	3754	6238	1.9938	3.44E-05	0.004545
Ccr5	1729	2053	869	929	0.79052	3.68E-05	0.004809
Fam71b	689	850	346	244	1.089118	4.01E-05	0.005081
Sv2c	131	299	54	45	1.835009	4.60E-05	0.005704
Ifngr1	1868	2571	761	1165	0.934753	4.63E-05	0.005704
Ccl22	171	329	67	70	1.588646	5.74E-05	0.007002
Ube2c	1551	2179	682	959	0.912221	5.81E-05	0.007005
Ifng	218	780	35	35	3.557462	6.13E-05	0.007316
Irf1	1594	1701	803	801	0.753385	6.36E-05	0.007434
Moxd1	82	158	16	28	2.180315	6.61E-05	0.007563
Serpinb6b	4591	5273	2385	2688	0.678501	7.61E-05	0.00862
Cysltr1	564	984	215	350	1.187386	7.95E-05	0.008908
Il12rb2	8283	10522	4510	4972	0.707176	8.12E-05	0.008926
Ccnb2	980	1206	349	578	0.968764	8.29E-05	0.00902
Il13	438	808	251	139	1.377888	8.39E-05	0.009035
2810417H13Rik	1879	2313	785	1157	0.838085	8.51E-05	0.009078
Klrc2	66	140	8	23	2.475454	8.70E-05	0.009196
Anxa2	6993	8272	3480	4340	0.687667	9.43E-05	0.009712
Tgm2	6876	7509	2762	4261	0.763144	9.74E-05	0.00988
Ly6e	5323	7785	2995	3234	0.793189	0.000102	0.010159
Ccr1	62	223	23	26	2.267155	0.000116	0.011449
Serpinb9	2418	3110	1405	1200	0.796833	0.000124	0.012025
Zfp260	166	455	72	93	1.641501	0.000125	0.012028
Sub1	3319	4212	1625	2109	0.736578	0.000149	0.014107
Frmd4b	335	475	153	155	1.112663	0.000157	0.014558
Ly6a	8235	12081	4480	5355	0.769756	0.000157	0.014558
Il1r2	1972	2872	1186	1003	0.857992	0.00016	0.014646
Tgtp2	901	1213	415	541	0.869629	0.000175	0.015765
Il10	38	108	7	11	2.752087	0.000201	0.017434
Hmgb2	3686	4487	1752	2369	0.713286	0.000203	0.017434
Taf7	425	549	186	220	0.983726	0.000224	0.018562

Pgk1	19338	25133	9903	12887	0.689369	0.000236	0.019374
Rnf146	565	677	296	222	0.969229	0.000257	0.020841
Tgtp1	1204	1489	497	757	0.831509	0.000271	0.021767
Stmn1	2387	2919	1051	1552	0.755547	0.000286	0.022692
Cbx3	1415	1722	827	522	0.919323	0.000305	0.023856
Msmo1	1526	2105	816	947	0.763872	0.000329	0.025337
Rrm2	2608	3168	1237	1689	0.706769	0.000338	0.025853
Podnl1	488	546	195	259	0.910872	0.000342	0.025895
Cdc20	1207	1147	449	682	0.78397	0.000375	0.02755
Igtp	5719	6497	2884	3667	0.621923	0.000379	0.027658
Gimap1os	346	454	152	176	1.007079	0.000389	0.02822
Tubb5	16577	20330	8283	11211	0.646538	0.000448	0.032071
Arl6ip1	3163	4579	1712	2126	0.736527	0.000454	0.032291
Capn2	2084	2476	1114	1273	0.653675	0.000466	0.032953
Ier5	1265	1321	653	680	0.67197	0.000487	0.033861
Raly	2152	1602	904	996	0.697274	0.000499	0.033966
Sgk1	171	197	46	73	1.356312	0.000505	0.033966
Ckap2l	844	880	314	492	0.824851	0.000515	0.034436
Filip1	135	307	68	60	1.505337	0.00052	0.034518
Bub3	1920	2328	1049	1164	0.65987	0.000523	0.034518
Gm10184	411	497	148	232	0.985344	0.000528	0.034663
Irf8	1354	1037	410	695	0.841609	0.000637	0.040178
Tbx21	356	324	148	135	0.976092	0.000702	0.043758
Lgals1	14581	18995	9504	7319	0.704926	0.000723	0.04481
Ifitm1	184	543	102	124	1.413903	0.000741	0.045657
Chchd10	1501	2358	930	909	0.786531	0.000756	0.046331
Ly6c1	2125	3108	408	858	1.787901	0.000776	0.047074
Nek2	566	602	250	305	0.794123	0.00079	0.047395
Ifi47	1366	1280	608	775	0.657139	0.000822	0.048756
Tcea1	1491	1922	873	871	0.684936	0.000844	0.049532
Adam8	477	518	217	246	0.822388	0.000885	0.050857
RP23-281H4.10	28	10	0	0	6.398617	0.000885	NA
Cenpw	184	239	65	87	1.20095	0.000913	0.050976
Gzma	378	1658	70	103	3.293984	0.000948	0.05205
Arl4c	1636	1869	582	1084	0.807416	0.00097	0.052705
Thy1	6810	9512	4287	4264	0.649434	0.000974	0.052705
Dut	1187	1264	592	698	0.645812	0.000982	0.052705
Gyg	747	912	381	439	0.736808	0.000983	0.052705
Spg20	471	538	242	220	0.839815	0.001008	0.053256
Ran	6143	7905	3720	3911	0.598317	0.001097	0.056862
Zbtb32	3068	3020	1639	1787	0.546262	0.001148	0.058414
Rnf6	975	1077	415	611	0.726722	0.001178	0.058931
Acadl	1710	2654	937	1210	0.749221	0.001201	0.059514
Gm17767	830	1110	290	572	0.90725	0.001236	0.059711
Tigit	439	550	222	234	0.834718	0.001238	0.059711
Hsd17b12	1209	1456	445	818	0.811553	0.001294	0.062134

AW112010	3221	4457	1851	2200	0.64486	0.001324	0.063325
Mad2l1	1497	1766	749	975	0.644189	0.001341	0.063855
Scin	28	189	18	18	2.318433	0.00138	0.064794
A630072M18Rik	133	162	32	60	1.413006	0.001383	0.064794
H2afx	684	636	266	377	0.761583	0.001384	0.064794
Ecm1	2135	1920	1010	1223	0.580031	0.001418	0.065832
Atp5f1	3023	3770	1768	1944	0.590911	0.001432	0.066205
Tuba1a	1977	2528	800	1409	0.761968	0.00144	0.066251
Hnrnpab	6565	8051	4017	4108	0.563711	0.001452	0.066251
Eif2s2	3789	4892	1811	2719	0.667583	0.001457	0.066251
Tagln2	4023	5368	2498	2549	0.613092	0.001469	0.066534
Fam162a	2320	2947	1228	1583	0.630096	0.001486	0.067026
Cdk1	1316	1255	426	806	0.794456	0.00153	0.068468
Glipr2	5403	7021	3084	3713	0.592494	0.001572	0.06959
Lilrb4a	2084	4069	1498	1317	0.843651	0.001574	0.06959
H3f3b	5744	7169	3127	3986	0.584121	0.00161	0.070912
Tubb4b	3291	3751	1708	2203	0.571799	0.001682	0.073759
Sephs2	759	1023	436	450	0.725841	0.001756	0.076246
Rfc3	965	1288	495	646	0.706306	0.001831	0.077896
Gpr183	998	1240	524	639	0.666456	0.001839	0.077896
Rpl39-ps	5416	5776	2920	3520	0.518033	0.001839	0.077896
9330175E14Rik	270	358	108	155	0.982436	0.001853	0.07817
Tuba1c	5786	6993	3331	3881	0.545978	0.001896	0.079678
Sco1	1392	1483	802	743	0.608459	0.00193	0.080816
Cdca3	1185	1363	504	793	0.703397	0.001978	0.082202
Pcna-ps2	1560	1889	771	1060	0.639223	0.002017	0.083183
Higd1a	866	1041	455	537	0.663697	0.002058	0.083945
Ywhaq	1072	1299	678	409	0.825711	0.002065	0.083945
Cdca8	1135	1214	587	684	0.605576	0.002098	0.084633
Arhgap19	521	534	185	307	0.829633	0.002154	0.086036
B2m	26714	33737	15352	18783	0.547193	0.002154	0.086036
Crabp2	659	944	410	357	0.776585	0.002203	0.086706
Gm10053	3260	3979	1990	2048	0.55893	0.002209	0.086706
Eif4ebp1	1401	2034	866	905	0.674435	0.002257	0.087269
Anp32e	2513	3068	1492	1610	0.565608	0.002284	0.08747
Bst1	24	79	8	6	2.596651	0.002286	0.08747
Lilr4b	1782	3230	1264	1134	0.779149	0.002354	0.088849
Fgr	28	59	7	4	2.693627	0.002368	NA
Seh1l	1369	1460	647	881	0.612881	0.002372	0.088933
Csrp1	1355	1572	706	882	0.604536	0.002372	0.088933
Aurka	601	739	252	394	0.781778	0.002427	0.090352
Gm43305	1076	1111	561	628	0.597203	0.002453	0.091022
Gm38326	11	19	0	0	6.069378	0.002461	NA
Spc25	567	776	252	386	0.80331	0.00254	0.093649
Gbp3	566	653	240	356	0.759316	0.002617	0.09575
Mthfd2	2692	3570	1699	1715	0.592012	0.002623	0.09575

Nrm	516	576	194	322	0.811416	0.002719	0.098083
Melk	563	695	267	357	0.736087	0.002805	0.099442
Srgn	12426	18519	8408	8205	0.614076	0.002838	0.099752
Birc5	1151	1296	510	771	0.661551	0.002841	0.099752
Hc	373	431	101	232	1.010486	0.002932	0.101739
Npm1	17121	20694	10707	11198	0.504898	0.003025	0.103929
Mid1ip1	255	570	184	128	1.112839	0.003066	0.104558
Asb2	316	468	161	191	0.877733	0.003095	0.104891
Kif4	893	967	431	560	0.631143	0.003101	0.104891
Cst3	1289	1696	779	821	0.617812	0.003167	0.106352
Ubb	4707	6216	2773	3329	0.562385	0.003169	0.106352
Icos	5031	6786	2974	3593	0.570313	0.003195	0.106546
Psmb8	4761	5956	2828	3270	0.534205	0.003237	0.107219
Tipin	1006	1230	516	674	0.634004	0.003347	0.109568
Padi2	1446	1579	673	969	0.607653	0.003378	0.110227
Top2a	10620	11461	4468	7554	0.608693	0.003405	0.110782
Ugt1a10	17	59	6	0	3.349469	0.003434	NA
Cmas	1245	1305	696	723	0.561495	0.003482	0.112301
Il22	79	350	8	25	3.456593	0.003594	0.114921
Asf1a	301	352	135	165	0.843443	0.00362	0.115101
Rps27l	1369	1651	828	835	0.576849	0.003754	0.117996
Fgl2	126	163	54	48	1.216204	0.003784	0.118262
Fkbp2	325	357	146	174	0.81184	0.003829	0.119003
Tesc	590	742	303	377	0.692642	0.00389	0.120255
Eya3	777	887	465	399	0.656452	0.004024	0.12272
Mir142hg	162	358	83	111	1.151288	0.004043	0.122912
Micu3	256	210	93	106	0.944168	0.004105	0.123763
Gm7030	33	20	2	2	3.439781	0.004143	NA
Eif1	4649	5686	2772	3202	0.511257	0.004213	0.126339
Tnfrsf9	11708	14833	6726	8530	0.522688	0.004319	0.128502
Eif5a	6931	9197	4507	4652	0.53386	0.004396	0.129399
F2rl2	26	101	14	8	2.240581	0.004446	0.130159
Tuba1b	6749	8534	3957	4840	0.519473	0.004508	0.131637
Ube2d3	4716	5539	2829	3178	0.490866	0.004543	0.132296
Chd7	10415	9041	5815	5692	0.46997	0.004577	0.132942
Batf3	535	771	335	308	0.737086	0.00464	0.133825
Epsti1	701	865	334	476	0.677671	0.004649	0.133825
Banf1	1193	1248	739	482	0.701071	0.004654	0.133825
Pcna	3633	4332	1847	2641	0.554618	0.004655	0.133825
Spc24	431	488	202	256	0.726909	0.004676	0.134069
Bub1b	1153	1254	562	763	0.585503	0.004708	0.134069
Sec61b	556	736	289	367	0.701514	0.004731	0.134069
Sdc4	278	367	132	163	0.851176	0.004736	0.134069
Acat1	1136	1428	670	738	0.583879	0.004837	0.136217
Ly6i	54	96	21	16	1.732148	0.004881	0.137107
Mctp2	1168	1424	646	785	0.578916	0.004903	0.137373

Ak6	1193	1493	726	753	0.578093	0.004978	0.138791
Rrm1	3055	3472	1685	2109	0.505073	0.005006	0.139222
Nhp2	1245	1505	815	633	0.633449	0.005051	0.139772
Hint1	2721	3074	1650	1737	0.491661	0.005275	0.14487
Capsl	103	172	47	48	1.252615	0.005292	0.144988
Cxcr6	31	142	18	19	1.951627	0.005379	0.14702
Trib3	912	1157	592	490	0.645759	0.005396	0.147121
Gadd45b	316	349	150	168	0.7828	0.005424	0.147521
Bhlhe40	11444	15113	6697	8538	0.526063	0.005462	0.147777
Commd2	314	342	158	153	0.791133	0.005463	0.147777
Gimap7	2225	2933	1121	1687	0.606626	0.005474	0.147777
Mycn	22	58	8	0	3.003019	0.005615	NA
Pank2	844	999	409	577	0.628413	0.005665	0.151098
Zfp263	423	612	249	259	0.745245	0.005821	0.153427
Tg	4003	5186	2549	2743	0.514791	0.005927	0.155487
Ccnb1	689	781	273	471	0.713908	0.005947	0.155636
Racgap1	1439	1560	679	990	0.572001	0.006068	0.157688
Sec61g	575	573	274	334	0.637103	0.006264	0.160917
Btf3	2902	3912	1723	2134	0.544513	0.006425	0.164277
Gm10282	261	280	101	146	0.856275	0.006525	0.166363
Cd34	3	23	0	0	5.870983	0.006538	NA
Gm4876	11	13	0	0	5.74454	0.00654	NA
Rps25	9910	11062	6243	6406	0.445356	0.006564	0.166363
2010107E04Rik	796	858	426	488	0.574704	0.006567	0.166363
Rnf19b	1958	2661	1330	1220	0.571125	0.006597	0.166743
Asf1b	491	544	233	303	0.67213	0.006625	0.167071
Gmnn	698	776	306	468	0.657092	0.006646	0.167208
Rras2	1133	1485	620	814	0.593279	0.006721	0.168206
Ptma	3595	4699	2082	2666	0.529064	0.006747	0.168206
Hmgb3	310	455	115	224	0.910612	0.006761	0.168206
Zdhhc2	308	340	144	169	0.769586	0.006793	0.168321
Acot7	1787	2210	1135	1145	0.526164	0.006881	0.169676
Fut7	123	134	55	34	1.23307	0.006938	0.170087
SnrPD-1	1292	1661	743	907	0.562207	0.006954	0.170087
Sh2d1a	513	541	281	269	0.652019	0.006959	0.170087
Dnajc9	1380	1799	777	994	0.568082	0.007074	0.172137
Degs1	727	928	420	474	0.608432	0.00719	0.172829
Kpna2	461	555	259	265	0.67186	0.007191	0.172829
Txndc12	724	885	430	442	0.600582	0.007191	0.172829
Nuf2	806	935	339	569	0.670157	0.007206	0.172829
Rpa2	751	846	392	485	0.586354	0.007399	0.175428
Prr11	690	773	290	472	0.67073	0.007486	0.176729
Ndc80	961	1103	403	687	0.652622	0.007625	0.178126
Hspa9	6970	8403	4652	4410	0.476507	0.007764	0.180589
Tpi1	6092	7438	3808	4250	0.467049	0.00778	0.180589
Gipc2	11	12	0	0	5.682507	0.007816	NA

Nsmce1	934	889	461	565	0.549303	0.007881	0.18177
Uqcr10	1141	1184	634	704	0.514954	0.007905	0.18177
Agpat4	624	701	264	424	0.674731	0.007925	0.18177
Vrk1	1040	1202	535	718	0.563804	0.007929	0.18177
Siglecf	36	63	12	8	2.017733	0.007975	0.182449
Glrx	325	592	162	255	0.869339	0.008047	0.182764
Chmp4b	4100	4579	2647	2509	0.464686	0.008183	0.185314
Myo1f	83	114	35	29	1.334586	0.008246	0.185979
Psma2	2355	3054	1542	1570	0.514463	0.008334	0.187585
Gar1	441	428	231	214	0.677663	0.008363	0.187838
Pttg1	708	877	307	515	0.678759	0.00847	0.189869
F2r	185	368	83	143	1.026185	0.008505	0.190264
Adamts14	149	118	19	67	1.377873	0.008543	0.190265
Tbca	969	976	487	615	0.54103	0.008547	0.190265
Tmsb4x	48579	63604	24044	39118	0.560824	0.008556	0.190265
Gcnt1	110	212	28	80	1.322839	0.008623	0.191371
Gnal	242	280	110	134	0.818275	0.00871	0.191778
Nfil3	1086	1492	645	779	0.578995	0.008742	0.191934
Pfn1	9574	11687	5783	6961	0.460226	0.008753	0.191934
Rps4x	28129	34584	18374	19402	0.44895	0.008782	0.1922
Angptl4	121	186	48	70	1.107393	0.008848	0.192874
Snrpf	1248	1578	798	799	0.539646	0.008936	0.194021
Cenpn	230	277	85	142	0.889317	0.008958	0.194021
Ldha	20800	25856	13552	14511	0.451595	0.009094	0.195926
Amn1	385	380	204	180	0.705262	0.009321	0.198897
2310031A07Rik	9	13	0	0	5.620569	0.009452	NA
Clic4	3342	3570	2104	2049	0.448873	0.009472	0.200574
Fam49b	3179	3941	1967	2247	0.476912	0.009522	0.200865
H1f0	1002	1283	648	623	0.561357	0.009592	0.201293
Slc7a5	3363	3599	2205	1840	0.492393	0.009611	0.201293
Rpl31	2932	3267	1747	1989	0.449757	0.009624	0.201293
Fasl	297	436	178	174	0.77509	0.009633	0.201293
Coq7	179	161	65	79	0.95845	0.009696	0.202236
Klf10	228	230	78	127	0.887511	0.009739	0.202755
Bre	680	867	426	412	0.599786	0.009766	0.202846
Tpx2	1734	1786	795	1212	0.538056	0.00987	0.203092
Mat2a	2714	2616	1651	1509	0.465312	0.00987	0.203092
Calm2	4448	5454	2858	3063	0.459987	0.009982	0.203972
Exosc3	545	746	349	327	0.648368	0.010065	0.205139
Gzmc	201	656	82	83	2.100396	0.010262	0.207417
Stra13	254	325	125	152	0.785574	0.010439	0.209616
Memo1	717	886	434	452	0.572687	0.010575	0.210289
Gm6793	2446	2731	1355	1732	0.46876	0.010669	0.211344
Actb	23279	40032	18784	15078	0.614276	0.01075	0.211878
Lysmd2	84	119	26	43	1.286881	0.010814	0.212381
Fam64a	510	537	237	322	0.62901	0.010853	0.212381

Fkbp5	722	871	448	431	0.57255	0.010891	0.212381
Ccna2	1980	2318	936	1494	0.552816	0.01108	0.215315
Gm5541	45	67	14	14	1.717996	0.01114	0.215887
Gm7729	1065	1419	664	737	0.546047	0.011236	0.216083
Fth1	4428	6123	3038	3100	0.499066	0.011261	0.21618
Ctsw	1018	1309	440	793	0.650544	0.011345	0.217413
Gm6485	431	454	221	248	0.634266	0.011395	0.217748
Lrrc40	520	647	282	345	0.618316	0.011402	0.217748
Gzmd	26	75	13	7	2.044775	0.011554	0.219351
Haus1	259	308	123	152	0.765666	0.011584	0.219351
Gm20634	1	85	4	4	3.157199	0.011769	NA
Sar1a	1698	1905	1059	1079	0.468737	0.01177	0.222113
Mapkapk2	4061	4304	2538	2593	0.420493	0.011833	0.22273
Tmem147	536	637	336	285	0.628524	0.011863	0.22273
Yeats4	684	881	404	460	0.577339	0.011945	0.222953
Snrpd3	1318	1537	821	848	0.491056	0.012027	0.223158
Ndufb5	839	937	454	558	0.532828	0.012067	0.223533
Scp2	945	981	526	590	0.505369	0.012117	0.22409
Klra7	25	49	7	6	2.22591	0.012119	NA
Cox7a2	1196	1468	680	855	0.518346	0.012274	0.226354
C1d	543	666	367	228	0.724864	0.012281	0.226354
Gadd45g	1190	1324	707	768	0.486977	0.012329	0.226735
Clic1	5550	6722	3361	4073	0.445114	0.012381	0.226735
Kcnk7	96	113	29	46	1.206147	0.012413	0.226735
Dars	1932	2395	1116	1423	0.492731	0.012434	0.226735
Oip5	315	292	135	168	0.722377	0.012462	0.226735
Gm43738	385	174	137	88	1.008546	0.012475	0.226735
D930048N14Rik	120	119	47	45	1.090885	0.012485	0.226735
Plk1	561	668	253	398	0.645829	0.012531	0.227201
Ska1	270	443	140	198	0.805206	0.012712	0.228988
Nasp	1753	1697	878	1170	0.475238	0.012784	0.229899
Krr1	869	1037	307	664	0.711742	0.012885	0.231348
Rpl39	3115	3809	1802	2327	0.469809	0.013041	0.232635
Gm5152	33	70	11	13	1.825998	0.013425	0.237582
Fam96a	839	1179	521	600	0.569517	0.01353	0.238548
H2-Q10	1144	1145	581	757	0.496966	0.013547	0.238548
Nutf2	699	802	447	375	0.578983	0.013566	0.238548
Dusp5	2087	2208	1237	1380	0.432873	0.014069	0.243492
Mrpl30	1278	1595	790	887	0.496343	0.0141	0.243492
Ndufs6	489	567	260	315	0.598139	0.014186	0.243492
Gm8773	6	14	0	0	5.486526	0.014266	NA
Pde4b	1402	2213	854	1141	0.584682	0.014274	0.243492
Lsm2	294	349	147	178	0.705591	0.014314	0.243597
Naca	6051	7672	3969	4346	0.441891	0.014351	0.243863
2700029M09Rik	776	894	422	529	0.534568	0.014401	0.243998
Casp6	988	1444	670	683	0.563946	0.014469	0.244477

Pcbp2	4189	4611	2635	2808	0.410126	0.014476	0.244477
Rfc4	491	622	248	347	0.629602	0.0145	0.244517
Gm21781	196	227	88	109	0.823984	0.014782	0.247388
Sytl2	103	155	40	60	1.095787	0.014904	0.248685
Rtn3	2422	2947	1071	1931	0.572975	0.014942	0.248685
Csf2	179	391	149	76	1.042426	0.014946	0.248685
Psmc3ip	177	199	73	97	0.868321	0.014949	0.248685
Vdac3-ps1	621	835	365	437	0.582454	0.01507	0.25005
Gm9803	261	339	144	153	0.732774	0.015076	0.25005
Cbx3-ps6	732	978	400	546	0.580137	0.015199	0.251714
Dnttip2	1160	1487	732	804	0.504385	0.015258	0.252306
Gm6169	669	923	431	447	0.576587	0.01534	0.253022
Id2	2010	4224	1036	1084	1.277734	0.015346	0.253022
Kit	18	48	2	8	2.470666	0.015388	NA
Il4i1	1446	1784	914	994	0.478069	0.015409	0.253053
Fcmr	77	123	38	32	1.22848	0.01541	0.253053
Rpl32	16676	21938	10593	12785	0.446543	0.015417	0.253053
Gmfg	2357	2820	1259	1797	0.487368	0.015474	0.253621
Lsm3	662	789	376	443	0.545758	0.015528	0.254135
Plekhg3	41	49	8	13	1.826752	0.015551	NA
Slc16a1	1332	1505	826	863	0.46484	0.015635	0.254853
Tars	2382	2748	1548	1559	0.439077	0.015641	0.254853
Pkm	29542	31112	16829	21297	0.392062	0.015703	0.255159
Hnrnpf	5753	6638	3488	4176	0.41428	0.015706	0.255159
2410006H16Rik	3493	4026	2000	2580	0.438668	0.015802	0.255528
Phyh	539	645	268	378	0.599963	0.016013	0.256403
Ywhae	3811	4480	2435	2660	0.420828	0.016059	0.256403
Cdkn3	165	251	72	112	0.906805	0.016081	0.256403
Ly6c2	65	93	19	32	1.361718	0.016122	0.256403
Sf3a3	1131	1426	734	752	0.499952	0.016144	0.256403
Rrp15	461	507	257	272	0.588593	0.016165	0.256403
Pfdn1	1032	1202	634	676	0.487576	0.016183	0.256403
Ppa1	982	1142	619	617	0.49657	0.016223	0.256403
Emb	6124	8488	4049	4664	0.467374	0.016236	0.256403
Prph	224	204	97	106	0.792426	0.01628	0.256403
Prnp	454	672	303	288	0.645988	0.016293	0.256403
Slc18a2	3	35	1	1	3.977095	0.01632	NA
Atp6v1g1	994	1392	676	671	0.541647	0.016327	0.256403
Ostf1	2536	3144	1529	1887	0.456234	0.016451	0.257098
Cdca5	481	564	271	303	0.583319	0.016481	0.257182
Ccl5	60	157	23	47	1.372725	0.016523	0.257182
Hprt	2339	3101	1522	1711	0.470992	0.016678	0.259182
Cdkn2d	1454	1741	794	1083	0.492731	0.016715	0.259396
Cetn3	779	953	486	505	0.522618	0.016891	0.261768
Rbm3	4767	6359	3147	3549	0.453013	0.017008	0.262848
Tpt1-ps3	38179	49214	24295	29256	0.428944	0.017122	0.264234

Eny2	477	628	261	337	0.609915	0.01721	0.265231
Cks2	377	505	215	251	0.64168	0.017397	0.266648
Cox17	764	883	469	479	0.513053	0.017555	0.268309
Fbxo5	674	675	271	462	0.610081	0.017579	0.268309
Selm	5	14	0	0	5.413697	0.01758	NA
Eif3c	7486	9318	5232	4995	0.430919	0.017815	0.270678
Nampt	1903	2167	1130	1347	0.437233	0.018054	0.273619
Nars	4124	4972	2902	2555	0.448797	0.018077	0.273619
Treml2	2914	3281	1596	2169	0.443341	0.0181	0.273619
Gm9531	1874	3102	1411	1365	0.559256	0.018143	0.273905
Tfb1m	206	208	88	109	0.791884	0.018238	0.274227
RP23-4F16.12	31	130	23	20	1.625459	0.018277	0.274227
5430427O19Rik	65	121	28	36	1.265058	0.018288	0.274227
Mcm7	2217	2301	1249	1533	0.420509	0.018321	0.274342
Pdlim1	842	989	511	555	0.498558	0.018349	0.274399
Sdr39u1	392	457	224	233	0.610391	0.018405	0.274857
Polr2g	886	1135	565	601	0.511711	0.018457	0.275266
Cd24a	398	812	287	312	0.73686	0.018587	0.276837
Eno1	6505	7977	3976	4941	0.42251	0.018903	0.280182
Anapc15	497	585	257	340	0.581749	0.018913	0.280182
Mzt1	958	1075	529	667	0.487646	0.019014	0.280184
Actg1	18034	14578	8896	11358	0.407462	0.019259	0.283046
Slc25a5	5460	6609	3512	3968	0.409896	0.019312	0.283451
Tubb4b-ps1	518	684	285	375	0.589604	0.019368	0.283591
H3f3a	1619	1827	923	1162	0.447262	0.019452	0.283928
Gm43223	31	77	15	12	1.716137	0.019473	0.283928
Ybx1	5587	5767	3692	3321	0.406142	0.019525	0.284311
Nup107	972	927	507	618	0.475208	0.019593	0.284932
Iars	2950	3752	2038	2008	0.443825	0.019783	0.285821
Gm6472	14813	18250	9362	11197	0.407168	0.019844	0.28588
Atp5e	1260	1523	762	898	0.466345	0.019878	0.28588
Sars	4458	5346	3055	2964	0.418578	0.01989	0.28588
Cenpb	513	577	350	164	0.777887	0.020031	0.287168
Aven	249	291	140	130	0.713732	0.020264	0.289011
Cbx3-ps7	401	523	233	266	0.609233	0.020264	0.289011
Leprotl1	1599	2231	1017	1229	0.492908	0.020435	0.28965
Ubl3	690	787	382	470	0.515316	0.020438	0.28965
Coro2a	1124	861	522	631	0.501655	0.020452	0.28965
Rpl41	16148	20529	10881	11842	0.409641	0.020492	0.28965
Psmb10	831	852	482	510	0.478887	0.020681	0.29122
Cetn4	164	253	94	98	0.83801	0.020985	0.292676
Ftl1	3496	4089	1725	2769	0.48562	0.021097	0.292676
Gm11226	122	161	52	69	0.950053	0.021104	0.292676
Poc1a	168	172	68	88	0.845659	0.021144	0.292676
Emilin2	85	85	21	39	1.23255	0.021166	0.292676
Cdh17	102	195	57	64	1.018144	0.0212	0.292676

Gdi2	4564	5314	2636	3467	0.418917	0.021217	0.292676
Enpp1	401	539	246	261	0.609176	0.021247	0.292676
Akr1c12	70	169	33	53	1.208522	0.02126	0.292676
Eps8	183	329	114	124	0.826609	0.0213	0.292722
Cul3	1582	1840	855	1190	0.468486	0.021393	0.292722
Trib2	2126	2307	973	1621	0.503904	0.021421	0.292722
Cd47	5606	6796	3402	4277	0.414725	0.021457	0.292801
Rab19	215	233	99	121	0.746662	0.021485	0.292801
Adora3	37	54	11	12	1.704098	0.021506	0.292801
Cox5a	1218	1642	815	862	0.488586	0.021578	0.293006
Calu	1282	1591	885	802	0.481103	0.021647	0.293006
Tpm4	5461	6818	3484	4111	0.414427	0.021653	0.293006
Etfb	943	1172	631	600	0.495382	0.021751	0.2938
Alg6	162	191	90	68	0.868125	0.021765	0.2938
Mgl2	4	14	0	0	5.336997	0.021829	NA
Hmgn2-ps1	116	119	47	50	0.993126	0.021922	0.295203
Anxa6	5793	6219	3332	4233	0.389507	0.022006	0.295256
Dpy30	431	517	239	284	0.578939	0.022046	0.295261
Phgdh	2132	2159	1377	1237	0.426059	0.02218	0.296521
Tex30	271	391	147	190	0.698748	0.022358	0.297824
Snx6	956	1314	647	665	0.508651	0.022564	0.299129
Ghitm	5529	7207	3996	3724	0.43635	0.022709	0.299981
Tardbp	2766	2849	1663	1870	0.386557	0.022749	0.30015
Serinc3	5425	7124	3446	4234	0.431476	0.022861	0.301263
Gpnmb	47	92	13	29	1.466004	0.022984	0.302175
Atp11a	1257	737	472	620	0.587058	0.02305	0.302441
1190002F15Rik	323	345	137	211	0.667888	0.02315	0.303279
Srp9	1197	1587	757	888	0.480589	0.023223	0.303867
Ndufv2	962	1215	619	664	0.481172	0.023395	0.305209
Uqcrb	823	962	491	562	0.481081	0.023412	0.305209
Slc43a3	951	995	586	573	0.461683	0.02351	0.305434
Hsp90ab1	26331	30411	17905	18034	0.374452	0.023525	0.305434
Rpl38	2151	2231	1356	1369	0.400187	0.023535	0.305434
Rala	633	688	384	378	0.508389	0.023642	0.306103
Commd1	221	315	141	121	0.745659	0.023751	0.306488
Dhfr	547	609	293	366	0.532543	0.023754	0.306488
Rbm8a	1156	1452	792	747	0.475304	0.023978	0.308628
Cdc45	764	931	456	535	0.495065	0.024009	0.308628
Ncaph	1223	1318	597	902	0.488897	0.024087	0.308628
Ino80c	443	486	266	248	0.567104	0.024264	0.309822
Snrpb2	1001	1369	611	769	0.50413	0.024368	0.310745
Osgin2	531	468	261	303	0.542544	0.024488	0.3116
Eif2s1	2321	3061	1608	1648	0.442327	0.024664	0.313026
Dtx3l	1708	1818	937	1227	0.427454	0.024684	0.313026
Plin2	766	1084	561	472	0.55254	0.024843	0.31356
Pex7	244	260	110	146	0.700102	0.024861	0.31356

Ccl3	564	1661	313	254	1.688179	0.024876	0.31356
Ckap2	665	709	355	446	0.500125	0.024988	0.31356
Bcl2a1b	628	807	411	408	0.525284	0.025005	0.31356
Eif3e	5373	6709	3355	4149	0.40999	0.025196	0.314832
Cdc42se1	1558	1597	630	1173	0.540752	0.025438	0.31602
Nme2	2919	3653	1860	2195	0.417963	0.025459	0.31602
Sfr1	1692	2398	1128	1301	0.473334	0.025661	0.31722
Selenof	1884	2087	1199	1267	0.40415	0.025673	0.31722
Ehd1	817	885	549	411	0.532411	0.02592	0.319206
Tmem176a	49	61	10	22	1.51627	0.025986	0.319649
Ccdc142os	18	26	2	4	2.605405	0.026109	NA
Cxcr4	360	522	185	280	0.652982	0.026182	0.319649
Itgax	4	13	0	0	5.254116	0.026197	NA
Mrpl35	457	637	290	315	0.574088	0.026215	0.319649
Rpl35	6721	8201	4490	4896	0.387487	0.026382	0.320953
Asnsd1	988	1177	652	637	0.463154	0.026479	0.321451
Myadm	21	35	5	5	2.204463	0.026496	NA
Nucks1	1927	1994	1096	1350	0.40181	0.026653	0.32284
Ptrh2	319	380	181	195	0.612545	0.02668	0.32284
Tmem258	812	897	484	536	0.462798	0.026908	0.32446
Etv6	2680	2741	1590	1843	0.378146	0.026931	0.32446
Plcg2	147	163	54	86	0.874585	0.026982	0.324567
Emc7	901	1097	554	636	0.467697	0.027014	0.324567
Actr3	7734	6255	4721	3654	0.444269	0.027311	0.326879
Fkbp3	1295	1669	751	1014	0.473519	0.027391	0.326879
Mrpl19	672	750	403	433	0.483744	0.027399	0.326879
Crem	1098	1688	808	790	0.518984	0.02759	0.328106
Mrpl54	411	532	273	241	0.588345	0.027618	0.328106
Susd1	269	305	149	152	0.647373	0.02772	0.328592
Ywhah	2807	3685	1953	2022	0.425275	0.027748	0.328592
Sae1	1299	1703	843	960	0.456043	0.027809	0.328962
Ramp2	21	0	0	0	5.5342	0.027884	NA
Nup37	316	382	164	211	0.619443	0.027983	0.330542
Cyp11a1	1254	1425	614	966	0.490986	0.028085	0.331089
S100a11	1008	1574	725	754	0.522973	0.028253	0.33234
Sdhd	1369	1700	805	1048	0.452115	0.028347	0.33234
Mrps18c	455	554	266	304	0.543765	0.028446	0.33234
Aimp1	1175	1309	713	808	0.426649	0.028454	0.33234
Gbp7	916	1158	499	714	0.500949	0.028597	0.333313
Ube2s	535	582	263	373	0.537787	0.028852	0.335583
Hemgn	38	42	7	13	1.729629	0.028919	NA
Txnip	4091	4787	2286	3216	0.416658	0.02895	0.336014
Aldoc	942	1074	470	713	0.497032	0.029098	0.336927
Pycr1	303	401	203	163	0.654138	0.029119	0.336927
Gm10076	2633	3428	1694	2033	0.423731	0.02912	0.336927
Ncoa4	283	275	173	84	0.812681	0.029241	0.337974

Rhoa	4594	6121	3101	3526	0.413907	0.029339	0.33856
Vimp	707	868	453	472	0.485072	0.029488	0.339421
Hmox1	136	220	71	92	0.852391	0.029586	0.340193
Ppp3cb	1421	961	603	784	0.499386	0.029907	0.34247
Wtap	1017	1151	708	550	0.492853	0.029975	0.342892
Hn1	1680	2450	1188	1269	0.468655	0.030036	0.343228
Oaz2	323	134	74	125	0.922137	0.030127	0.343923
Gm20489	0	20	0	0	5.496356	0.03014	NA
Snhg20	276	273	153	132	0.656229	0.030256	0.344511
Rps17	3421	3903	2256	2385	0.375266	0.030272	0.344511
Cdkn2aipnl	809	980	491	575	0.467589	0.030373	0.344807
Ttk	467	522	227	329	0.556932	0.030532	0.345495
Ahcyl2	526	1005	371	447	0.62948	0.030545	0.345495
Vdac1	2558	3118	1683	1863	0.397757	0.030683	0.346345
Arf1	4049	5307	2946	2805	0.41694	0.030813	0.346761
Cox7a2l	3140	4343	2122	2456	0.430461	0.030931	0.347204
C1qtnf6	37	37	7	11	1.764659	0.030958	NA
Hif1a	22778	31844	15349	18430	0.416272	0.030978	0.347204
Nudt4	562	581	287	376	0.508283	0.031027	0.347398
BC004004	865	974	545	563	0.447254	0.031351	0.349792
Eif4g2	8733	9836	5699	6273	0.351493	0.031766	0.352474
Serpina3f	28	28	5	6	2.066935	0.032021	NA
Ptpn13	178	219	101	93	0.74704	0.032031	0.354224
Sdf4	10501	12137	6837	7730	0.355393	0.032174	0.355222
Rbbp8	694	784	385	491	0.477207	0.03241	0.356686
Rpl23a-ps3	12037	15395	8395	8917	0.382264	0.032435	0.356686
H2-Aa	24	64	8	14	1.735254	0.032503	NA
Asns	1987	2672	1528	1214	0.474199	0.032701	0.357508
Klri2	18	25	1	5	2.585637	0.032742	NA
Tcp1	7193	8710	4821	5309	0.369548	0.032749	0.357508
Fabp5	1024	1074	621	667	0.420767	0.032795	0.357508
Sgol1	642	724	312	476	0.521524	0.032847	0.357508
Mir17hg	647	704	346	451	0.484245	0.033018	0.357785
Rps9	18762	24876	13357	14025	0.39046	0.033129	0.358287
Apobr	161	153	56	90	0.831611	0.033242	0.358295
H2-Q7	6954	5624	3029	4695	0.429857	0.033278	0.358295
Alcam	398	575	257	281	0.574783	0.033356	0.358295
Mif	4237	5362	2627	3372	0.402308	0.033663	0.359334
Iigp1	439	344	162	260	0.617814	0.033692	0.359334
Uqcrfs1	1622	1918	1042	1162	0.402654	0.033744	0.359334
Stk32c	196	191	88	105	0.722912	0.033777	0.359334
Mrpl50	400	475	249	242	0.548564	0.033846	0.359334
Eif1ax	1517	2087	1079	1095	0.446572	0.034026	0.360608
Cdc25a	856	847	530	492	0.448751	0.03408	0.360831
Smc2	2218	2664	1222	1773	0.432352	0.034331	0.36245
Rad21	4157	4946	2470	3263	0.391584	0.03437	0.362522

Med21	332	405	176	229	0.587272	0.034433	0.362836
Nupr1	93	174	57	56	0.959397	0.034481	0.362995
Rpl27-ps3	8283	11275	5560	6636	0.403823	0.034655	0.363794
Cops4	1316	1508	832	921	0.406443	0.034756	0.364164
Cacybp	1431	1686	991	917	0.421313	0.034956	0.365919
Rab38	2	14	0	0	5.170184	0.035063	NA
1810037I17Rik	1470	1910	967	1104	0.427232	0.035248	0.367128
Gars	4408	5720	3225	3064	0.402152	0.035347	0.367128
Magohb	201	307	120	137	0.704597	0.03535	0.367128
Psma6	1861	2002	1177	1268	0.377067	0.035351	0.367128
Cyb5a	1951	1826	1020	1349	0.395347	0.035377	0.367128
Mlkl	285	332	167	166	0.605303	0.0354	0.367128
Apoo-ps	102	112	52	36	0.987956	0.035402	0.367128
Oxsm	231	287	116	154	0.664089	0.035436	0.367128
Serpina3g	120	178	77	54	0.893831	0.035494	0.367128
Gm2000	10680	13490	7229	8145	0.372712	0.0355	0.367128
Rps12-ps3	893	1101	552	652	0.448899	0.035502	0.367128
Lin7c	1447	1622	900	1020	0.395738	0.035715	0.367462
Ppib	3448	4385	2378	2540	0.389794	0.035717	0.367462
Txn1	2307	2880	1604	1628	0.399001	0.035758	0.367462
Ccr4	669	1152	486	535	0.556365	0.035933	0.368837
Lamtor2	399	462	230	259	0.535206	0.035981	0.368991
Txnl1	1501	1940	1065	1033	0.429222	0.036085	0.369076
Hmgb1-ps2	560	702	323	412	0.503282	0.036467	0.37089
Coa6	165	167	79	82	0.759859	0.036615	0.372051
Abracl	644	815	316	517	0.53954	0.036761	0.372848
Tagap	3676	3489	2433	2030	0.390957	0.037074	0.374163
Xkr5	115	89	35	50	0.984981	0.037093	0.374163
Atf5	706	947	543	374	0.555507	0.03723	0.374531
Gpsm2	321	383	142	236	0.627478	0.037551	0.376108
Cops2	1980	2397	1333	1412	0.390761	0.037557	0.376108
Tceb1	968	1189	598	715	0.437583	0.037645	0.376653
Fads2	291	322	160	175	0.589554	0.037701	0.376668
Lamtor5	527	635	301	377	0.499745	0.03772	0.376668
Gm28229	62	82	6	38	1.466881	0.03805	0.378982
Eef1a1	160708	230005	117482	126004	0.401703	0.038094	0.378982
Slc35a2	193	314	124	131	0.711321	0.03814	0.378982
C3ar1	4	11	0	0	5.072541	0.03824	NA
Kif11	2801	3051	1503	2165	0.400364	0.038266	0.379103
Gm8730	17305	22683	12827	12317	0.384446	0.038353	0.379456
Tmem256	442	602	263	330	0.539546	0.03837	0.379456
Vps37b	1638	1884	884	1283	0.427666	0.038553	0.380591
Plp2	1813	2453	1119	1486	0.437248	0.038652	0.381097
Park7	1502	2019	884	1244	0.453834	0.038673	0.381097
Gm14005	16	18	1	3	2.821878	0.038747	NA
Pyhin1	648	620	269	457	0.534122	0.038781	0.381617

Cdc123	814	977	481	601	0.449583	0.038821	0.381617
Golt1b	730	981	499	513	0.475186	0.038829	0.381617
Rbbp4	2692	2948	1627	1984	0.364526	0.038929	0.382068
Brca2	472	523	272	308	0.497495	0.03909	0.382739
Tmpo	3844	4023	2107	2905	0.375202	0.03913	0.382739
Nudc	1017	991	618	621	0.410873	0.03914	0.382739
Rbx1	743	894	407	567	0.47484	0.039253	0.383405
Sssca1	199	313	147	103	0.74216	0.039397	0.383803
Nmi	397	432	252	211	0.550406	0.039589	0.384447
Nap1l1	2779	3403	1871	2046	0.377054	0.039639	0.384466
Prep	963	1068	648	584	0.433212	0.039843	0.385158
Dnajc19	264	318	145	170	0.606094	0.03994	0.385705
Cenpm	281	293	132	179	0.60746	0.040061	0.385935
Tmem30a	3185	3843	2033	2439	0.373751	0.040081	0.385935
Ywhab	1955	2362	1367	1332	0.39259	0.040112	0.385935
Prim2	485	552	274	332	0.496055	0.040122	0.385935
Ptpn14	288	337	163	180	0.584181	0.040196	0.385935
Snrnp27	816	955	535	539	0.437417	0.040232	0.385935
H2afv	725	857	392	550	0.473777	0.040277	0.385935
Cep19	314	386	188	200	0.569169	0.040328	0.386091
Ero1l	5969	7153	3508	4811	0.383337	0.04038	0.386254
Slmo2	995	1286	682	702	0.438096	0.04044	0.386384
Mcm5	1885	1795	1096	1255	0.364495	0.040517	0.386384
Dlgap5	1013	1024	527	722	0.429583	0.040572	0.386384
Pla2g12a	1059	1037	713	509	0.482265	0.040821	0.387144
Rpl26	10903	12787	7524	7819	0.343437	0.0409	0.387348
Cs	3270	3828	2242	2293	0.36254	0.040914	0.387348
Knop1	291	589	207	249	0.67388	0.040948	0.387348
Btbd10	461	485	283	263	0.505693	0.04112	0.388329
Tmem219	444	541	286	281	0.512267	0.041252	0.388329
Rps20	12223	12689	7583	8830	0.321399	0.041297	0.388329
Atf4	6392	7995	4611	4531	0.369657	0.041516	0.388784
Entpd5	317	239	127	169	0.629803	0.041607	0.388784
Gemin6	171	201	99	85	0.727312	0.041623	0.388784
Ptpn3	767	941	518	509	0.449343	0.041625	0.388784
Gab2	199	205	85	121	0.696108	0.041743	0.389039
Prpf38a	1032	1301	689	740	0.425555	0.041758	0.389039
Bloc1s1	424	466	193	308	0.557641	0.04189	0.389386
Il16	1700	1580	855	1195	0.402072	0.041915	0.389386
Mrpl18	670	922	409	528	0.489337	0.04198	0.389471
Mis18a	524	597	289	371	0.48699	0.042047	0.389772
Tbc1d31	847	898	508	567	0.417009	0.042272	0.391064
Rpl14	5795	7285	4085	4261	0.36565	0.042292	0.391064
Rad23b	2096	2318	1394	1421	0.36463	0.042412	0.391842
Calm3	1742	2083	1160	1252	0.383337	0.042466	0.392013
Cdc6	469	494	273	291	0.488553	0.042556	0.392172

Ninj1	172	204	100	87	0.71983	0.042605	0.392172
Rpl21	6519	8064	4385	4985	0.358255	0.04278	0.39281
Cstb	557	805	363	427	0.507982	0.04283	0.39281
Mrps33	382	482	204	284	0.549987	0.042983	0.392866
Ly6g	54	97	24	32	1.157584	0.043243	0.39404
Rtcb	2288	2742	1550	1646	0.371911	0.043271	0.39404
Scd1	840	1167	601	595	0.463484	0.043339	0.394171
Magoh	646	844	389	495	0.476918	0.043489	0.394171
Lsm4	954	1105	604	669	0.412327	0.0435	0.394171
Hmgb1-ps1	68	80	27	30	1.09524	0.043619	0.394171
Strap	2346	2658	1565	1639	0.359927	0.043629	0.394171
Cfl1	11774	14924	8239	8907	0.357534	0.04366	0.394171
AI480526	342	271	152	184	0.585498	0.043706	0.394171
Anapc16	541	628	309	384	0.476308	0.043814	0.394613
Smim20	206	282	131	122	0.662729	0.043925	0.394651
Eno1b	1514	1863	936	1173	0.402164	0.044102	0.395919
Smg8	349	390	174	242	0.553709	0.044359	0.396306
Gm5611	178	235	96	115	0.69058	0.044631	0.397127
Il6st	590	434	261	329	0.514181	0.044735	0.397127
Vbp1	1568	1689	871	1178	0.392831	0.044874	0.397127
Snrpe	836	1258	508	719	0.499414	0.044949	0.397127
Mrpl20	831	973	495	610	0.429053	0.044952	0.397127
1110059E24Rik	408	522	243	292	0.519409	0.045042	0.397601
Cct7	3618	4256	2660	2277	0.384046	0.045268	0.398456
Gm9493	4968	6214	3319	3852	0.361768	0.045318	0.398456
Rp9	832	900	457	604	0.430345	0.045493	0.398475
Gm8897	5	9	0	0	4.970132	0.04569	NA
Sdhb	1422	1601	864	1047	0.382825	0.045713	0.399827
Atp5b	10813	13345	8052	7300	0.366811	0.045739	0.399827
Gm5424	743	634	351	475	0.459368	0.046065	0.401522
Uqcrq	1641	2095	1233	1048	0.422986	0.046184	0.401608
Ndufc1	245	315	100	190	0.683836	0.046187	0.401608
Egr1	2714	2785	1639	1936	0.340907	0.046731	0.403837
Cox6b1	1649	2119	1075	1284	0.397493	0.046944	0.404292
Ddias	498	610	330	320	0.484573	0.046964	0.404292
Gm15500	34905	42362	24520	25793	0.336342	0.046966	0.404292
Mrpl39	521	590	327	333	0.46727	0.047057	0.404733
Trdv1	16	6	0	1	4.177879	0.047063	NA
Ranbp1	1608	1823	1068	1113	0.37022	0.047259	0.405115
Rplp0	37252	49054	27208	28061	0.36045	0.047272	0.405115
Eif6	1264	1448	890	784	0.407551	0.047353	0.405115
Ap3s1-ps1	254	360	154	179	0.60421	0.047441	0.405235
Ccni	3323	3816	1982	2601	0.363416	0.047511	0.405521
Gm43720	10	4	0	0	4.958781	0.047715	NA
Actr2	5008	5825	3271	3780	0.339618	0.047855	0.407836
Cd83	178	110	69	64	0.823534	0.047999	0.408222

Gm5428	3337	3983	2217	2505	0.352009	0.048048	0.408222
Tmem14c	556	791	327	456	0.509817	0.048147	0.40875
Tmem167	772	985	497	572	0.437401	0.048401	0.409686
Snrpb	1926	2173	1259	1372	0.357591	0.048539	0.409686
Ccdc181	177	219	96	108	0.67638	0.048548	0.409686
Anapc5	2140	2208	1309	1508	0.345117	0.048601	0.409686
Zmat2	1045	1265	680	758	0.403008	0.048645	0.409686
Eif4e	1634	2153	1096	1271	0.399151	0.048705	0.409686
Gm867	47	70	25	14	1.288595	0.048732	0.409686
Wdr1	4834	5695	3210	3636	0.340585	0.048737	0.409686
Gtf2f2	611	755	387	435	0.452293	0.048877	0.410418
Msra	127	118	45	68	0.841374	0.04903	0.411212
Ndufb3	632	633	363	407	0.433987	0.049318	0.412381
Hnrnpa1	4710	5123	3012	3447	0.325524	0.049545	0.413312
Polr2b	1345	1430	772	986	0.380845	0.049593	0.413312
Mmp12	1	23	1	0	4.299328	0.04966	NA
Mcm3	1926	1849	952	1419	0.397286	0.049693	0.413312
Hspb11	357	447	192	265	0.540494	0.049786	0.413312
ThumPD-1	1131	1446	777	821	0.407414	0.049788	0.413312
Pwp1	840	990	526	605	0.414128	0.049802	0.413312
